# A case-only study to identify genetic modifiers of breast cancer risk for *BRCA1/BRCA2* mutation carriers

**DOI:** 10.1038/s41467-020-20496-3

**Published:** 2021-02-17

**Authors:** Juliette Coignard, Michael Lush, Jonathan Beesley, Tracy A. O’Mara, Joe Dennis, Jonathan P. Tyrer, Daniel R. Barnes, Lesley McGuffog, Goska Leslie, Manjeet K. Bolla, Muriel A. Adank, Simona Agata, Thomas Ahearn, Kristiina Aittomäki, Irene L. Andrulis, Hoda Anton-Culver, Volker Arndt, Norbert Arnold, Kristan J. Aronson, Banu K. Arun, Annelie Augustinsson, Jacopo Azzollini, Daniel Barrowdale, Caroline Baynes, Heiko Becher, Marina Bermisheva, Leslie Bernstein, Katarzyna Białkowska, Carl Blomqvist, Stig E. Bojesen, Bernardo Bonanni, Ake Borg, Hiltrud Brauch, Hermann Brenner, Barbara Burwinkel, Saundra S. Buys, Trinidad Caldés, Maria A. Caligo, Daniele Campa, Brian D. Carter, Jose E. Castelao, Jenny Chang-Claude, Stephen J. Chanock, Wendy K. Chung, Kathleen B. M. Claes, Christine L. Clarke, GEMO Study Collaborators, Ophélie Bertrand, Sandrine Caputo, Anaïs Dupré, Marine Le Mentec, Muriel Belotti, Anne-Marie Birot, Bruno Buecher, Emmanuelle Fourme, Marion Gauthier-Villars, Lisa Golmard, Claude Houdayer, Virginie Moncoutier, Antoine de Pauw, Claire Saule, Olga Sinilnikova, Sylvie Mazoyer, Francesca Damiola, Laure Barjhoux, Carole Verny-Pierre, Mélanie Léone, Nadia Boutry-Kryza, Alain Calender, Sophie Giraud, Olivier Caron, Marine Guillaud-Bataille, Brigitte Bressac-de-Paillerets, Yves- Jean Bignon, Nancy Uhrhammer, Christine Lasset, Valérie Bonadona, Pascaline Berthet, Dominique Vaur, Laurent Castera, Tetsuro Noguchi, Cornel Popovici, Hagay Sobol, Violaine Bourdon, Tetsuro Noguchi, Audrey Remenieras, Catherine Noguès, Isabelle Coupier, Pascal Pujol, Aurélie Dumont, Françoise Révillion, Claude Adenis, Danièle Muller, Emmanuelle Barouk-Simonet, Françoise Bonnet, Virginie Bubien, Nicolas Sevenet, Michel Longy, Christine Toulas, Rosine Guimbaud, Laurence Gladieff, Viviane Feillel, Dominique Leroux, Hélène Dreyfus, Christine Rebischung, Magalie Peysselon, Fanny Coron, Laurence Faivre, Amandine Baurand, Caroline Jacquot, Geoffrey Bertolone, Sarab Lizard, Fabienne Prieur, Marine Lebrun, Caroline Kientz, Sandra Fert Ferrer, Véronique Mari, Laurence Vénat-Bouvet, Capucine Delnatte, Stéphane Bézieau, Isabelle Mortemousque, Florence Coulet, Chrystelle Colas, Florent Soubrier, Mathilde Warcoin, Johanna Sokolowska, Myriam Bronner, Marie-Agnès Collonge-Rame, Alexandre Damette, Paul Gesta, Hakima Lallaoui, Jean Chiesa, Denise Molina-Gomes, Olivier Ingster, EMBRACE Collaborators, Helen Gregory, Zosia Miedzybrodzka, Patrick J. Morrison, Kai-ren Ong, Alan Donaldson, Mark T. Rogers, M. John Kennedy, Mary E. Porteous, Carole Brewer, Rosemarie Davidson, Louise Izatt, Angela Brady, Julian Barwell, Julian Adlard, Claire Foo, Fiona Lalloo, Lucy E. Side, Jacqueline Eason, Alex Henderson, Lisa Walker, Rosalind A. Eeles, Jackie Cook, Katie Snape, Diana Eccles, Alex Murray, Emma McCann, J. Margriet Collée, Don M. Conroy, Kamila Czene, Mary B. Daly, Peter Devilee, Orland Diez, Yuan Chun Ding, Susan M. Domchek, Thilo Dörk, Isabel dos-Santos-Silva, Alison M. Dunning, Miriam Dwek, Diana M. Eccles, A. Heather Eliassen, Christoph Engel, Mikael Eriksson, D. Gareth Evans, Peter A. Fasching, Henrik Flyger, Florentia Fostira, Eitan Friedman, Lin Fritschi, Debra Frost, Manuela Gago-Dominguez, Susan M. Gapstur, Judy Garber, Vanesa Garcia-Barberan, Montserrat García-Closas, José A. García-Sáenz, Mia M. Gaudet, Simon A. Gayther, Andrea Gehrig, Vassilios Georgoulias, Graham G. Giles, Andrew K. Godwin, Mark S. Goldberg, David E. Goldgar, Anna González-Neira, Mark H. Greene, Pascal Guénel, Lothar Haeberle, Eric Hahnen, Christopher A. Haiman, Niclas Håkansson, Per Hall, Ute Hamann, Patricia A. Harrington, Steven N. Hart, Wei He, Frans B. L. Hogervorst, Antoinette Hollestelle, John L. Hopper, Darling J. Horcasitas, Peter J. Hulick, David J. Hunter, Evgeny N. Imyanitov, KConFab Investigators, Stephen Fox, Ian Campbell, Amanda Spurdle, Penny Webb, Anna de Fazio, Margaret Tassell, Judy Kirk, Geoff Lindeman, Melanie Price, Melissa Southey, Roger Milne, Sid Deb, David Bowtell, HEBON Investigators, Annemieke H. van der Hout, Ans M. W. van den Ouweland, Arjen R. Mensenkamp, Carolien H. M. van Deurzen, Carolien M. Kets, Caroline Seynaeve, Christi J. van Asperen, Cora M. Aalfs, Encarna B. Gómez Garcia, Flora E. van Leeuwen, G. H. de Bock, Hanne E. J. Meijers-Heijboer, Inge M. Obdeijn, J. Margriet Collée, J. J. P. Gille, Jan C. Oosterwijk, Juul T. Wijnen, Lizet E. van der Kolk, Maartje J. Hooning, Margreet G. E. M. Ausems, Marian J. E. Mourits, Marinus J. Blok, Matti A. Rookus, Muriel A. Adank, Rob B. van der Luijt, T. C. T. E. F. van Cronenburg, Carmen C. van der Pol, Nicola S. Russell, Sabine Siesling, Lucy Overbeek, R. Wijnands, Judith L. de Lange, ABCTB Investigators, Christine Clarke, Dinny Graham, Mythily Sachchithananthan, Deborah Marsh, Rodney Scott, Robert Baxter, Desmond Yip, Jane Carpenter, Alison Davis, Nirmala Pathmanathan, Peter Simpson, Agnes Jager, Anna Jakubowska, Paul A. James, Uffe Birk Jensen, Esther M. John, Michael E. Jones, Rudolf Kaaks, Pooja Middha Kapoor, Beth Y. Karlan, Renske Keeman, Elza Khusnutdinova, Johanna I. Kiiski, Yon-Dschun Ko, Veli-Matti Kosma, Peter Kraft, Allison W. Kurian, Yael Laitman, Diether Lambrechts, Loic Le Marchand, Jenny Lester, Fabienne Lesueur, Tricia Lindstrom, Adria Lopez-Fernández, Jennifer T. Loud, Craig Luccarini, Arto Mannermaa, Siranoush Manoukian, Sara Margolin, John W. M. Martens, Noura Mebirouk, Alfons Meindl, Austin Miller, Roger L. Milne, Marco Montagna, Katherine L. Nathanson, Susan L. Neuhausen, Heli Nevanlinna, Finn C. Nielsen, Katie M. O’Brien, Olufunmilayo I. Olopade, Janet E. Olson, Håkan Olsson, Ana Osorio, Laura Ottini, Tjoung-Won Park-Simon, Michael T. Parsons, Inge Sokilde Pedersen, Beth Peshkin, Paolo Peterlongo, Julian Peto, Paul D. P. Pharoah, Kelly-Anne Phillips, Eric C. Polley, Bruce Poppe, Nadege Presneau, Miquel Angel Pujana, Kevin Punie, Paolo Radice, Johanna Rantala, Muhammad U. Rashid, Gad Rennert, Hedy S. Rennert, Mark Robson, Atocha Romero, Maria Rossing, Emmanouil Saloustros, Dale P. Sandler, Regina Santella, Maren T. Scheuner, Marjanka K. Schmidt, Gunnar Schmidt, Christopher Scott, Priyanka Sharma, Penny Soucy, Melissa C. Southey, John J. Spinelli, Zoe Steinsnyder, Jennifer Stone, Dominique Stoppa-Lyonnet, Anthony Swerdlow, Rulla M. Tamimi, William J. Tapper, Jack A. Taylor, Mary Beth Terry, Alex Teulé, Darcy L. Thull, Marc Tischkowitz, Amanda E. Toland, Diana Torres, Alison H. Trainer, Thérèse Truong, Nadine Tung, Celine M. Vachon, Ana Vega, Joseph Vijai, Qin Wang, Barbara Wappenschmidt, Clarice R. Weinberg, Jeffrey N. Weitzel, Camilla Wendt, Alicja Wolk, Siddhartha Yadav, Xiaohong R. Yang, Drakoulis Yannoukakos, Wei Zheng, Argyrios Ziogas, Kristin K. Zorn, Sue K. Park, Mads Thomassen, Kenneth Offit, Rita K. Schmutzler, Fergus J. Couch, Jacques Simard, Georgia Chenevix-Trench, Douglas F. Easton, Nadine Andrieu, Antonis C. Antoniou

**Affiliations:** 1 Genetic Epidemiology of Cancer team, Inserm, U900, Paris, France; 2 Institut Curie Paris, Paris, France; 3 Mines ParisTech Fontainebleau, Paris, France; 4 Centre for Cancer Genetic Epidemiology, Department of Public Health and Primary Care, University of Cambridge, Cambridge, UK; 5 PSL University Paris, Paris, France; 6 Paris Sud University, Orsay, France; 7 Department of Genetics and Computational Biology QIMR Berghofer Medical Research Institute, Brisbane, QLD Australia; 8 Centre for Cancer Genetic Epidemiology, Department of Oncology University of Cambridge, Cambridge, UK; 9 Family Cancer Clinic, The Netherlands Cancer Institute, Antoni van Leeuwenhoek hospital, Amsterdam, The Netherlands; 10 Immunology and Molecular Oncology, Unit Veneto Institute of Oncology IOV – IRCCS, Padua, Italy; 11 Division of Cancer Epidemiology and Genetics National Cancer Institute, National Institutes of Health, Department of Health and Human Services, Bethesda, MD USA; 12 Department of Clinical Genetics, Helsinki University Hospital University of Helsinki, Helsinki, Finland; 13 Fred A. Litwin Center for Cancer Genetics Lunenfeld-Tanenbaum Research Institute of Mount Sinai Hospital, Toronto, ON Canada; 14 Department of Molecular Genetics University of Toronto, Toronto, ON Canada; 15 Department of Epidemiology, Genetic Epidemiology Research Institute University of California Irvine, Irvine, CA USA; 16 Division of Clinical Epidemiology and Aging Research German Cancer Research Center (DKFZ), Heidelberg, Germany; 17 Department of Gynaecology and Obstetrics University Hospital of Schleswig-Holstein, Campus Kiel, Christian-Albrechts University Kiel, Kiel, Germany; 18 Institute of Clinical Molecular Biology University Hospital of Schleswig-Holstein, Campus Kiel, Christian-Albrechts University Kiel, Kiel, Germany; 19 Department of Public Health Sciences, and Cancer Research Institute Queen’s University, Kingston, ON Canada; 20 Department of Breast Medical Oncology University of Texas MD Anderson Cancer Center, Houston, TX USA; 21 Department of Cancer Epidemiology, Clinical Sciences Lund University, Lund, 22242 Sweden; 22 Unit of Medical Genetics, Department of Medical Oncology and Hematology Fondazione IRCCS Istituto Nazionale dei Tumori di Milano, Milan, Italy; 23 Institute for Medical Biometrics and Epidemiology University Medical Center Hamburg-Eppendorf, Hamburg, Germany; 24 Institute of Biochemistry and Genetics Ufa Federal Research Centre of the Russian Academy of Sciences, Ufa, Russia; 25 Department of Population Sciences Beckman Research Institute of City of Hope, Duarte, CA USA; 26 Department of Genetics and Pathology Pomeranian Medical University Szczecin, Szczecin, Poland; 27 Department of Oncology, Helsinki University Hospital University of Helsinki, Helsinki, Finland; 28 Department of Oncology Örebro University Hospital, Örebro, Sweden; 29 Copenhagen General Population Study, Herlev and Gentofte Hospital Copenhagen University Hospital, Herlev, Denmark; 30 Department of Clinical Biochemistry, Herlev and Gentofte Hospital Copenhagen University Hospital, Herlev, Denmark; 31 Faculty of Health and Medical Sciences University of Copenhagen, Copenhagen, Denmark; 32 Division of Cancer Prevention and Genetics IEO, European Institute of Oncology IRCCS, Milan, Italy; 33 Department of Oncology Lund University and Skåne University Hospital, Lund, Sweden; 34 Dr. Margarete Fischer-Bosch-Institute of Clinical Pharmacology, Stuttgart, Germany; 35 iFIT-Cluster of Excellence University of Tübingen, Tübingen, Germany; 36 Division of Preventive Oncology, German Cancer Research Center (DKFZ) and National Center for Tumor Diseases (NCT), Heidelberg, Germany; 37 German Cancer Consortium (DKTK), German Cancer Research Center (DKFZ), Heidelberg, Germany; 39 Molecular Epidemiology Group, C080 German Cancer Research Center (DKFZ), Heidelberg, Germany; 40 Molecular Biology of Breast Cancer, University Womens Clinic Heidelberg University of Heidelberg, Heidelberg, Germany; 41 Department of Medicine Huntsman Cancer Institute, Salt Lake City, UT USA; 42 Molecular Oncology Laboratory CIBERONC, Hospital Clinico San Carlos, IdISSC (Instituto de Investigación Sanitaria del Hospital Clínico San Carlos), Madrid, Spain; 43 SOD Genetica Molecolare University Hospital, Pisa, Italy; 44 Department of Biology University of Pisa, Pisa, Italy; 45 Division of Cancer Epidemiology German Cancer Research Center (DKFZ), Heidelberg, Germany; 46 Behavioral and Epidemiology Research Group American Cancer Society Atlanta, Atlanta, GA USA; 47 Oncology and Genetics Unit Instituto de Investigacion Sanitaria Galicia Sur (IISGS), Xerencia de Xestion Integrada de Vigo-SERGAS, Vigo, Spain; 48 Cancer Epidemiology Group, University Cancer Center Hamburg (UCCH) University Medical Center Hamburg-Eppendorf, Hamburg, Germany; 49 Departments of Pediatrics and Medicine, Columbia University, New York, NY USA; 50 Centre for Medical Genetics Ghent University, Gent, Belgium; 51 Westmead Institute for Medical Research University of Sydney, Sydney, NSW Australia; 52 Department of Clinical Genetics Erasmus University Medical Center, Rotterdam, The Netherlands; 53 Department of Medical Epidemiology and Biostatistics, Karolinska Institutet, Stockholm, Sweden; 54 Department of Clinical Genetics Fox Chase Cancer Center Philadelphia, Philadelphia, PA USA; 55 Department of Pathology Leiden University Medical Center, Leiden, The Netherlands; 56 Department of Human Genetics Leiden University Medical Center, Leiden, The Netherlands; 57 Oncogenetics Group Vall d’Hebron Institute of Oncology (VHIO), Barcelona, Spain; 58 Clinical and Molecular Genetics Area University Hospital Vall d’Hebron, Barcelona, Spain; 59 Basser Center for BRCA, Abramson Cancer Center University of Pennsylvania, Philadelphia, PA USA; 60 Gynaecology Research Unit, Hannover Medical School, Hannover, Germany; 61 Department of Non-Communicable Disease Epidemiology London School of Hygiene and Tropical Medicine, London, UK; 62 School of Life Sciences University of Westminster, London, UK; 63 Faculty of Medicine University of Southampton, Southampton, UK; 64 Channing Division of Network Medicine, Department of Medicine, Brigham and Women’s Hospital, Harvard Medical School, Boston, MA USA; 65 Department of Epidemiology Harvard TH Chan School of Public Health, Boston, MA USA; 66 Institute for Medical Informatics, Statistics and Epidemiology University of Leipzig, Leipzig, Germany; 67 Genomic Medicine, Division of Evolution and Genomic Sciences The University of Manchester, Manchester Academic Health Science Centre, Manchester Universities Foundation Trust, St Mary’s Hospital, Manchester, UK; 68 Genomic Medicine, North West Genomics hub Manchester Academic Health Science Centre, Manchester Universities Foundation Trust, St Mary’s Hospital, Manchester, UK; 69 David Geffen School of Medicine, Department of Medicine Division of Hematology and Oncology University of California at Los Angeles, Los Angeles, CA USA; 70 Department of Gynecology and Obstetrics, Comprehensive Cancer Center ER-EMN University Hospital Erlangen, Friedrich-Alexander-University, Erlangen-Nuremberg, Erlangen, Germany; 71 Department of Breast Surgery, Herlev and Gentofte Hospital Copenhagen University Hospital, Herlev, Denmark; 72 Molecular Diagnostics Laboratory, INRASTES National Centre for Scientific Research íDemokritosí, Athens, Greece; 73 The Susanne Levy Gertner Oncogenetics Unit Chaim Sheba Medical Center, Ramat Gan, Israel; 74 Sackler Faculty of Medicine Tel Aviv University, Ramat Aviv, Israel; 75 School of Public Health Curtin University, Perth, Western Australia Australia; 76 Genomic Medicine Group, Galician Foundation of Genomic Medicine Instituto de Investigación Sanitaria de Santiago de Compostela (IDIS), Complejo Hospitalario Universitario de Santiago, SERGAS, Santiago de Compostela, Spain; 77 Moores Cancer Center University of California, San Diego La Jolla, CA USA; 78 Division of Cancer Genetics and Prevention, Dana-Farber Cancer Institute, Boston, MA USA; 79 Medical Oncology Department, Hospital Clínico San Carlos Instituto de Investigación Sanitaria San Carlos (IdISSC), Centro Investigación Biomédica en Red de Cáncer (CIBERONC), Madrid, Spain; 80 Center for Bioinformatics and Functional Genomics and the Cedars Sinai Genomics Core Cedars-Sinai Medical Center, Los Angeles, CA USA; 81 Department of Human Genetics University Würzburg, Würzburg, Germany; 82 Department of Medical Oncology University Hospital of Heraklion, Heraklion, Greece; 83 Cancer Epidemiology Division Cancer Council Victoria, Melbourne, VIC Australia; 84 Centre for Epidemiology and Biostatistics, Melbourne School of Population and Global Health, The University of Melbourne, Melbourne, VIC Australia; 85 Precision Medicine, School of Clinical Sciences at Monash Health Monash University, Clayton, VIC Australia; 86 Department of Pathology and Laboratory Medicine, University of Kansas Medical Center, Kansas City, KS USA; 87 Department of Medicine, McGill University, Montréal, QC Canada; 88 Division of Clinical Epidemiology, Royal Victoria Hospital McGill University Montréal, Montréal, QC Canada; 89 Huntsman Cancer Institute and Department of Dermatology, University of Utah School of Medicine, Salt Lake City, UT USA; 90 Human Cancer Genetics Programme Spanish National Cancer Research Centre (CNIO), Madrid, Spain; 91 Clinical Genetics Branch, Division of Cancer Epidemiology and Genetics National Cancer Institute, Bethesda, MD USA; 92 Cancer & Environment Group, Center for Research in Epidemiology and Population Health (CESP) INSERM, University Paris-Sud, University Paris-Saclay, Villejuif, France; 93 Department of Gynaecology and Obstetrics, University Hospital Erlangen Friedrich-Alexander University Erlangen-Nuremberg, Comprehensive Cancer Center Erlangen-EMN, Erlangen, Germany; 94 Center for Hereditary Breast and Ovarian Cancer Faculty of Medicine and University Hospital Cologne, University of Cologne, Cologne, Germany; 95 Center for Integrated Oncology (CIO) Faculty of Medicine and University Hospital Cologne, University of Cologne, Cologne, Germany; 96 Department of Preventive Medicine, Keck School of Medicine University of Southern California, Los Angeles, CA USA; 97 Institute of Environmental Medicine Karolinska Institutet, Stockholm, Sweden; 98 Department of Oncology, Södersjukhuset, Stockholm, Sweden; 99 Molecular Genetics of Breast Cancer German Cancer Research Center (DKFZ), Heidelberg, Germany; 100 Department of Health Sciences Research, Mayo Clinic, Rochester, MN USA; 101 Department of Medical Oncology, Family Cancer Clinic Erasmus MC Cancer Institute, Rotterdam, The Netherlands; 102 New Mexico Oncology Hematology Consultants, University of New Mexico, Albuquerque, NM USA; 103 Center for Medical Genetics NorthShore University HealthSystem, Evanston, IL USA; 104 The University of Chicago Pritzker School of Medicine Chicago, Chicago, IL USA; 105 Program in Genetic Epidemiology and Statistical Genetics Harvard TH Chan School of Public Health Boston, Boston, MA USA; 106 Nuffield Department of Population Health University of Oxford, Oxford, UK; 107 NN Petrov Institute of Oncology, St. Petersburg, Russia; 108 Independent Laboratory of Molecular Biology and Genetic Diagnostics Pomeranian Medical University, Szczecin, Poland; 109 Sir Peter MacCallum Department of Oncology The University of Melbourne, Melbourne, VIC Australia; 110 Parkville Familial Cancer Centre Peter MacCallum Cancer Center, Melbourne, VIC Australia; 111 Department of Clinical Genetics Aarhus, University Hospital, Aarhus, Denmark; 112 Department of Medicine, Division of Oncology, Stanford University School of Medicine, Stanford, CA USA; 114 Department of Epidemiology and Population Health, Stanford University School of Medicine, Stanford, CA USA; 115 Division of Genetics and Epidemiology The Institute of Cancer Research, London, UK; 116 Faculty of Medicine University of Heidelberg, Heidelberg, Germany; 117 David Geffen School of Medicine, Department of Obstetrics and Gynecology University of California at Los Angeles, Los Angeles, CA USA; 118 Womenís Cancer Program at the Samuel Oschin Comprehensive Cancer Institute Cedars-Sinai Medical Center, Los Angeles, CA USA; 119 Division of Molecular Pathology The Netherlands Cancer Institute - Antoni van Leeuwenhoek Hospital, Amsterdam, The Netherlands; 120 Department of Genetics and Fundamental Medicine Bashkir State Medical University, Ufa, Russia; 121 Department of Obstetrics and Gynecology, Helsinki University Hospital University of Helsinki, Helsinki, Finland; 122 Department of Internal Medicine, Evangelische Kliniken Bonn gGmbH Johanniter Krankenhaus, Bonn, Germany; 123 Translational Cancer Research Area University of Eastern Finland, Kuopio, Finland; 124 Institute of Clinical Medicine, Pathology and Forensic Medicine University of Eastern Finland, Kuopio, Finland; 125 VIB Center for Cancer Biology, Leuven, Belgium; 126 Laboratory for Translational Genetics, Department of Human Genetics University of Leuven, Leuven, Belgium; 127 Epidemiology Program University of Hawaii Cancer Center, Honolulu, HI USA; 128 High Risk and Cancer Prevention Group Vall d’Hebron Institute of Oncology, Barcelona, Spain; 129 Department of Clinical Science and Education, Södersjukhuset Karolinska Institutet, Stockholm, Sweden; 130 Department of Gynecology and Obstetrics University of Munich, Campus Grosshadern, Munich, Germany; 131 NRG Oncology, Statistics and Data Management Center Roswell Park Cancer Institute, Buffalo, NY USA; 132 Center for Genomic Medicine Rigshospitalet, Copenhagen University Hospital, Copenhagen, Denmark; 133 Epidemiology Branch National Institute of Environmental Health Sciences, NIH Research Triangle Park, Durham, NC USA; 134 Center for Clinical Cancer Genetics The University of Chicago, Chicago, IL USA; 135 Centro de Investigación en Red de Enfermedades Raras (CIBERER), Madrid, Spain; 136 Department of Molecular Medicine University La Sapienza, Rome, Italy; 137 Molecular Diagnostics Aalborg University Hospital, Aalborg, Denmark; 138 Clinical Cancer Research Center Aalborg University Hospital, Aalborg, Denmark; 139 Department of Clinical Medicine Aalborg University, Aalborg, Denmark; 140 Lombardi Comprehensive Cancer Center, Georgetown University, Washington, DC USA; 141 Genome Diagnostics Program IFOM - the FIRC (Italian Foundation for Cancer Research) Institute of Molecular Oncology, Milan, Italy; 142 Translational Research Laboratory IDIBELL (Bellvitge Biomedical Research Institute), Catalan Institute of Oncology, CIBERONC, Barcelona, Spain; 143 Leuven Multidisciplinary Breast Center, Department of Oncology Leuven Cancer Institute, University Hospitals Leuven, Leuven, Belgium; 144 Unit of Molecular Bases of Genetic Risk and Genetic Testing, Department of Research Fondazione IRCCS Istituto Nazionale dei Tumori (INT), Milan, Italy; 145 Clinical Genetics Karolinska Institutet, Stockholm, Sweden; 146 Department of Basic Sciences Shaukat Khanum Memorial Cancer Hospital and Research Centre (SKMCH & RC), Lahore, Pakistan; 147 Clalit National Cancer Control Center Carmel Medical Center and Technion Faculty of Medicine, Haifa, Israel; 148 Clinical Genetics Service, Department of Medicine Memorial Sloan-Kettering Cancer Center, New York, NY USA; 149 Medical Oncology Department Hospital Universitario Puerta de Hierro, Madrid, Spain; 150 Department of Oncology University Hospital of Larissa, Larissa, Greece; 151 Department of Epidemiology, Mailman School of Public Health Columbia University, New York, NY USA; 152 Cancer Genetics and Prevention Program University of California San Francisco, San Francisco, CA USA; 153 Division of Psychosocial Research and Epidemiology The Netherlands Cancer Institute - Antoni van Leeuwenhoek hospital, Amsterdam, The Netherlands; 154 Institute of Human Genetics Hannover Medical School, Hannover, Germany; 155 Department of Internal Medicine, Division of Medical Oncology University of Kansas Medical Center, Westwood, KS USA; 156 Genomics Center, Centre Hospitalier Universitaire de Québec - Université Laval Research Center, Québec City, QC Canada; 157 Department of Clinical Pathology The University of Melbourne, Melbourne, VIC Australia; 158 Population Oncology BC Cancer, Vancouver, BC Canada; 159 School of Population and Public Health University of British Columbia, Vancouver, BC Canada; 160 Clinical Genetics Research Lab, Department of Cancer Biology and Genetics Memorial Sloan-Kettering Cancer Center, New York, NY USA; 161 The Curtin UWA Centre for Genetic Origins of Health and Disease Curtin University and University of Western Australia, Perth, Western Australia Australia; 162 Service de Génétique Institut Curie, Paris, France; 163 Department of Tumour Biology INSERM U830, Paris, France; 164 Université Paris Descartes, Paris, France; 165 Division of Breast Cancer Research Institute of Cancer Research, London, UK; 167 Epigenetic and Stem Cell Biology Laboratory National Institute of Environmental Health Sciences, NIH Research Triangle Park, Triangle Park, NC USA; 168 Hereditary Cancer Program ONCOBELL-IDIBELL-IDIBGI-IGTP, Catalan Institute of Oncology, CIBERONC, Barcelona, Spain; 169 Department of Medicine Magee-Womens Hospital, University of Pittsburgh School of Medicine, Pittsburgh, PA USA; 170 Program in Cancer Genetics, Departments of Human Genetics and Oncology McGill University, Montréal, QC Canada; 171 Department of Medical Genetics, National Institute for Health Research Cambridge Biomedical Research Center, University of Cambridge, Cambridge, UK; 172 Department of Cancer Biology and Genetics The Ohio State University, Columbus, OH USA; 173 Institute of Human Genetics Pontificia Universidad Javeriana, Bogota, Colombia; 174 Department of medicine University Of Melbourne, Melbourne, VIC Australia; 175 Department of Medical Oncology Beth Israel Deaconess Medical Center, Boston, MA USA; 176 Department of Health Science Research, Division of Epidemiology Mayo Clinic, Rochester, MN USA; 177 Fundación Pública Galega Medicina Xenómica-SERGAS, Instituto de Investigación Sanitaria Santiago de Compostela (IDIS); CIBERER, Santiago de Compostela, Spain; 178 Biostatistics and Computational Biology Branch National Institute of Environmental Health Sciences, NIH Research Triangle Park, Triangle Park, NC USA; 179 Clinical Cancer Genomics City of Hope, Duarte, CA USA; 180 Department of Surgical Sciences Uppsala University, Uppsala, Sweden; 181 Department of Oncology Mayo Clinic, Rochester, MN USA; 182 Division of Epidemiology, Department of Medicine, Vanderbilt Epidemiology Center, Vanderbilt-Ingram Cancer Center Vanderbilt University School of Medicine, Nashville, TN USA; 183 Magee-Womens Hospital, University of Pittsburgh School of Medicine, Pittsburgh, PA USA; 184 Department of Preventive Medicine Seoul National University College of Medicine, Seoul, Korea; 185 Department of Biomedical Sciences Seoul National University Graduate School, Seoul, Korea; 186 Cancer Research Institute Seoul National University, Seoul, Korea; 187 Department of Clinical Genetics Odense University Hospital, Odence C, Denmark; 188 Department of Laboratory Medicine and Pathology Mayo Clinic, Rochester, MN USA; 189 Unité Mixte de Génétique Constitutionnelle des Cancers Fréquents, Hospices Civils de Lyon - Centre Léon Bérard, Lyon, France; 190 Institut Gustave Roussy, Villejuif, France; 191 Centre Jean Perrin, Clermont–Ferrand, France; 192 Centre Léon Bérard, Lyon, France; 193 Centre François Baclesse, Caen, France; 194 Institut Paoli Calmettes, Marseille, France; 195 CHU Arnaud-de-Villeneuve, Montpellier, France; 196 Centre Oscar Lambret, Lille, France; 197 Centre Paul Strauss, Strasbourg, France; 198 Institut Bergonié, Bordeaux, France; 199 Institut Claudius Regaud, Toulouse, France; 200 CHU, Grenoble, France; 201 CHU, Dijon, France; 202 CHU, St-Etienne, France; 203 Hôtel Dieu Centre Hospitalier, Chambéry, France; 204 Centre Antoine Lacassagne, Nice, France; 205 CHU, Limoges, France; 206 CHU, Nantes, France; 207 CHU Bretonneau, Tours, France; 208 Centre Hospitalier de, Bourges, France; 209 Groupe Hospitalier Pitié- Salpétrière, Paris, France; 210 CHU Vandoeuvre-, les-Nancy, France; 211 CHU, Besançon, France; 212 CHU Poitiers, Centre Hospitalier d’Angoulême, Poitiers, France; 213 Centre Hospitalier de Niort, Niort, France; 214 Centre Hospitalier de La Rochelle, La Rochelle, France; 215 CHU Nîmes Carémeau, Nîmes, France; 216 CHU, Poissy, France; 217 CHU, Angers, France; 218 North of Scotland Regional Genetics Service, NHS Grampian & University of Aberdeen, Foresterhill, Aberdeen, UK; 219 Northern Ireland Regional Genetics Centre, Belfast Health and Social Care Trust, and Department of Medical Genetics, Queens University Belfast, Belfast, UK; 220 West Midlands Regional Genetics Service, Birmingham Women’s Hospital Healthcare NHS Trust, Edgbaston, Birmingham, UK; 221 Clinical Genetics Department, St Michael’s Hospital, Bristol, UK; 222 All Wales Medical Genetics Services, University Hospital of Wales, Cardiff, UK; 223 Academic Unit of Clinical and Molecular Oncology, Trinity College Dublin and St James’s Hospital, Dublin, Eire; 224 South East of Scotland Regional Genetics Service, Western General Hospital, Edinburgh, UK; 225 Department of Clinical Genetics, Royal Devon & Exeter Hospital, Exeter, UK; 226 Clinical Genetics, Southern General Hospital, Glasgow, UK; 227 Clinical Genetics, Guy’s and St. Thomas’ NHS Foundation Trust, London, UK; 228 North West Thames Regional Genetics Service, Kennedy-Galton Centre, Harrow, UK; 229 Leicestershire Clinical Genetics Service, University Hospitals of Leicester NHS Trust, Leicester, UK; 230 Yorkshire Regional Genetics Service, Leeds, UK; 231 Department of Clinical Genetics, Alder Hey Hospital, Eaton Road, Liverpool, UK; 232 Genetic Medicine, Manchester Academic Health Sciences Centre, Central Manchester University Hospitals NHS Foundation Trust, Manchester, UK; 233 North East Thames Regional Genetics Service, Great Ormond Street Hospital for Children NHS Trust, London, UK; 234 Nottingham Clinical Genetics Service, Nottingham University Hospitals NHS Trust, Nottingham, UK; 235 Institute of Genetic Medicine, Centre for Life, Newcastle Upon Tyne Hospitals NHS Trust, Newcastle upon Tyne, UK; 236 Oxford Regional Genetics Service, Churchill Hospital, Oxford, UK; 237 Oncogenetics Team, The Institute of Cancer Research and Royal Marsden NHS Foundation Trust, London, UK; 238 Sheffield Clinical Genetics Service, Sheffield Children’s Hospital, Sheffield, UK; 239 South West Thames Regional Genetics Service, St.Georges Hospital, Cranmer Terrace, Tooting, London, UK; 240 All Wales Medical Genetics Services, Singleton Hospital, Swansea, UK; 241 All Wales Medical Genetics Service, Glan Clwyd Hospital, Rhyl, UK; 242 Peter MacCallum Cancer Centre, Melbourne, Australia; 243 Westmead Millenium Institute, Sydney, Australia; 244 BCNA delegate, Community Representative, Melbourne, Australia; 245 Westmead Hospital, Sydney, Australia; 246 Walter and Eliza Hall Institute, Melbourne, Australia; 247 University of Sydney, Sydney, Australia; 248 Melbourne Health, Melbourne, Australia; 249 Garvan Institute of Medical Research, Sydney, Australia; 250 Department of Genetics, University Medical Center Groningen, Groningen, The Netherlands; 251 Department of Clinical Genetics, Family Cancer Clinic, Erasmus University Medical Center, Rotterdam, The Netherlands; 252 Department of Human Genetics, Radboud University Medical Center, Nijmegen, The Netherlands; 253 Department of Pathology, Family Cancer Clinic, Erasmus University Medical Center, Rotterdam, The Netherlands; 254 Department of Clinical Genetics, Leiden University Medical Center, Leiden, The Netherlands; 255 Department of Clinical Genetics, Academic Medical Center, Amsterdam, The Netherlands; 256 Department of Clinical Genetics, MUMC, Maastricht, The Netherlands; 257 Department of Epidemiology, Netherlands Cancer Institute, Amsterdam, The Netherlands; 258 Department of Oncological Epidemiology, University Medical Center, Groningen University, Groningen, The Netherlands; 259 Department of Clinical Genetics, VU University Medical Centre, Amsterdam, The Netherlands; 260 Department of Radiology, Family Cancer Clinic, Erasmus University Medical Center, Rotterdam, The Netherlands; 261 Department of Medical Genetics, University Medical Center Utrecht, Utrecht, The Netherlands; 262 Department of Gynaecological Oncology, University Medical Center, Groningen University, Groningen, The Netherlands; 263 Department of Clinical Genetics, Maastricht University Medical Center, Maastricht, The Netherlands; 264 Department of Oncological and Endocrine Surgery, University Medical Center Utrecht, Utrecht, The Netherlands; 265 Department of Radiotherapy, Netherlands Cancer Institute, Amsterdam, The Netherlands; 266 The Netherlands Comprehensive Cancer Organization (IKNL), Utrecht, The Netherlands; 267 Foundation PALGA (The Nationwide Network and Registry of Histo- and Cytopathology in the Netherlands), Houten, The Netherlands; 268 University of Technology Sydney, Translational Oncology Group, School of Life Sciences, Faculty of Science, Ultimo, NSW Australia; 269 School of Biomedical Sciences, University of Newcastle, Newcastle; Hunter Medical Research Institute and NSW Health Pathology North, Newcastle, Australia; 270 Kolling Institute of Medical Research, University of Sydney, St Leonards, NSW Australia; 271 Department of Medical Oncology, The Canberra Hospital, Canberra, ACT Australia; 272 Scientific Platforms, The Westmead Institute for Medical Research, The University of Sydney, Sydney, NSW Australia; 273 The Canberra Hospital, Garran, ACT; The Australian National University, Canberra, ACT Australia; 274 Westmead Breast Cancer Institute, Western Sydney Local Health District, Westmead, New South Wales, Australia; 275 University of Sydney, Western Clinical School, Westmead, New South Wales, Australia; 276 UQ Centre for Clinical Research, Faculty of Medicine, The University of Queensland, Herston, QLD Australia

**Keywords:** Breast cancer, Cancer epidemiology, Cancer genetics, Risk factors

## Abstract

Breast cancer (BC) risk for *BRCA1* and *BRCA2* mutation carriers varies by genetic and familial factors. About 50 common variants have been shown to modify BC risk for mutation carriers. All but three, were identified in general population studies. Other mutation carrier-specific susceptibility variants may exist but studies of mutation carriers have so far been underpowered. We conduct a novel case-only genome-wide association study comparing genotype frequencies between 60,212 general population BC cases and 13,007 cases with *BRCA1* or *BRCA2* mutations. We identify robust novel associations for 2 variants with BC for *BRCA1* and 3 for *BRCA2* mutation carriers, *P* < 10^−8^, at 5 loci, which are not associated with risk in the general population. They include rs60882887 at 11p11.2 where *MADD*, *SP11* and *EIF1*, genes previously implicated in BC biology, are predicted as potential targets. These findings will contribute towards customising BC polygenic risk scores for *BRCA1* and *BRCA2* mutation carriers.

## Abstract (web summary)

Breast cancer risk for *BRCA1/BRCA2* mutation carriers varies depending on other genetic factors. Here, the authors perform a case-only genome-wide association study and highlight novel loci associated with breast cancer risk for *BRCA1/BRCA2* mutation carriers.

## Introduction

Breast cancer (BC) is the most common cancer in women worldwide^1^ and BC family history is one of the most important risk factors for the disease. Women with a history of BC in a first-degree relative are about two times more likely to develop BC than women without a family history^2^. Around 15–20% of the familial risk of BC can be explained by rare mutations in the *BRCA1* or *BRCA2* genes^3^. A recent prospective cohort study estimated the cumulative risk of BC by 80 years to be 72% for *BRCA1* mutation carriers and 69% for *BRCA2* mutation carriers^4^. This study also demonstrated that BC risk for mutation carriers varies by family history of BC in first and second degree relatives, suggesting the existence of other genetic factors that modify BC risks^4^.

A total of 179 common BC susceptibility single nucleotide polymorphisms (SNPs) or small insertions or deletions (INDELs) have been identified through genome-wide association studies (GWAS) in the general population^1,5–35^. Although risk alleles at individual SNPs (hereafter used as a generic term to refer to common variants, which also includes the small INDELs) are associated with modest increases in BC risk, it has been shown that they combine multiplicatively on risk, resulting in substantial levels of BC risk stratification in the population^36–38^. Similarly, more than 50 of the common genetic BC susceptibility variants have also been shown to be associated with BC for *BRCA1* and *BRCA2* mutation carriers^5,6,15,18,20,39–48^ and their joint effects, summarised as polygenic risk scores (PRS), result in large differences in the absolute risks of developing BC for mutation carriers at the extremes of the PRS distribution^49^. BC GWAS for *BRCA1* and *BRCA2* mutation carriers have been carried out through the Consortium of Investigators of Modifiers of *BRCA1/2* (CIMBA)^50^. However, despite the large number of *BRCA1* and *BRCA2* mutation carriers included, the power to detect genetic modifiers of risk remains limited in comparison to that available in the general population^7^. To date, no variants specifically associated with BC risk for *BRCA1* and *BRCA2* carriers have been identified.

Here, we apply a novel strategy using a case-only GWAS design^51,52^, in which SNP genotype frequencies in 7,257 *BRCA1* and 5,097 *BRCA2* mutation carrier BC cases are compared to those in 60,212 BC cases from the Breast Cancer Association Consortium (BCAC), unselected for mutation status. We aim (1) to identify novel SNPs that modify BC risk for *BRCA1* or *BRCA2* mutation carriers but are not associated with risk in the general population and (2) for the known 179 BC susceptibility SNPs, assess whether there is evidence of an interaction between the SNPs and *BRCA1* or *BRCA2* mutations and therefore evaluate whether the SNP effect size estimates applicable to mutation carriers are different.

We identify robust novel associations for 2 variants with BC for *BRCA1* and 3 for *BRCA2* mutation carriers, *P* < 10^−8^, at 5 loci, which are not associated with risk in the general population. They include rs60882887 in 11p11.2 where *MADD*, *SP11* and *EIF1*, genes previously implicated in BC biology, are predicted as potential targets. These findings will contribute towards customising BC PRS for *BRCA1* and *BRCA2* mutation carriers.

## Results

### Sample characteristics

**Fig. 1:**
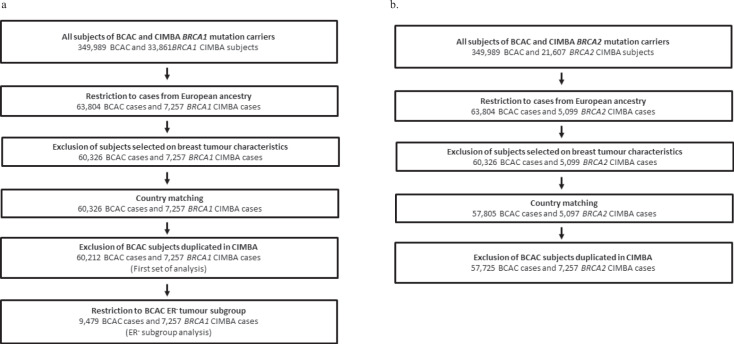
Case-only sample selection. Sample selection for **a** *BRCA1* and **b** *BRCA2* case-only analysis. *Four studies were excluded because they were included in clinical trials based on breast tumour characteristics as HER-2 receptor status (see Supplementary Table 2).

**Fig. 2:**
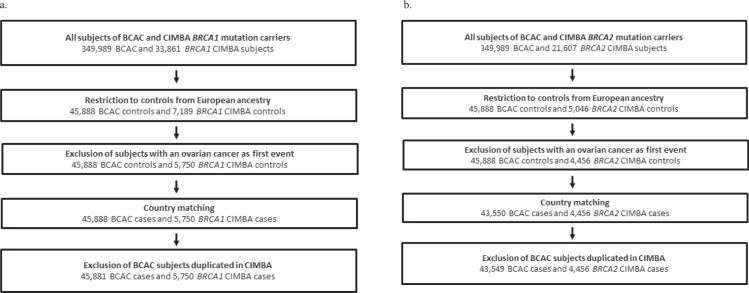
Control-only sample selection. Sample selection for **a** BRCA1 and **b** BRCA2 control-only analysis.

A total of 60,212 BCAC cases and 7,257 *BRCA1* mutation carrier cases were available for the BRCA1 case-only analyses and 57,725 BCAC cases and 5,097 *BRCA2* mutation carrier cases were available for the BRCA2 case-only analyses (Fig. 1). A total of 45,881 BCAC controls and 5,750 unaffected *BRCA1* mutation carriers were available for the BRCA1 control-only analyses and 43,549 BCAC controls and 4,456 unaffected *BRCA2* mutation carriers for the BRCA2 control-only analyses (see Fig. 2). Only women of European ancestry were included with 60.9% samples from European countries, 31.1% from the USA, 6.1% from Australia and 1.7% from Israel (Supplementary Tables 1–4). The mean age at BC diagnosis for mutation carrier cases in CIMBA was 42.5 years (40.9 for *BRCA1* mutation carriers; 44.1 for *BRCA2* mutation carriers) and 58.4 years for cases in BCAC.

**Fig. 3:**
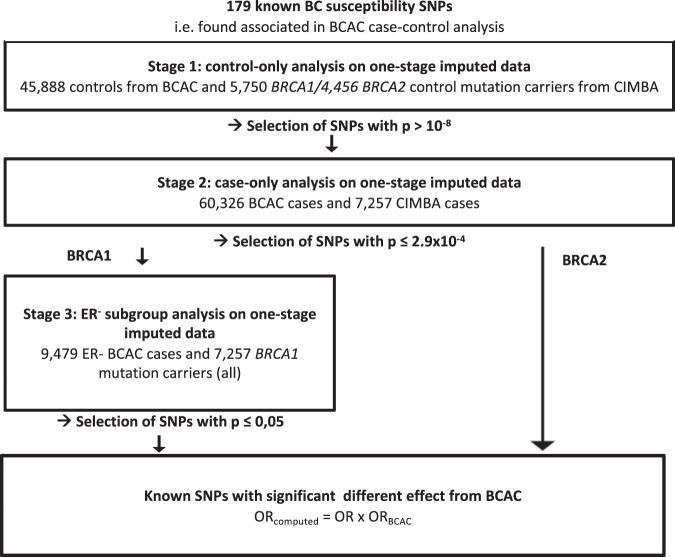
Analytical process for known BC susceptibility SNPs. Strategy followed for analysing the associations for the 179 known BC susceptibility SNPs.

**Fig. 4:**
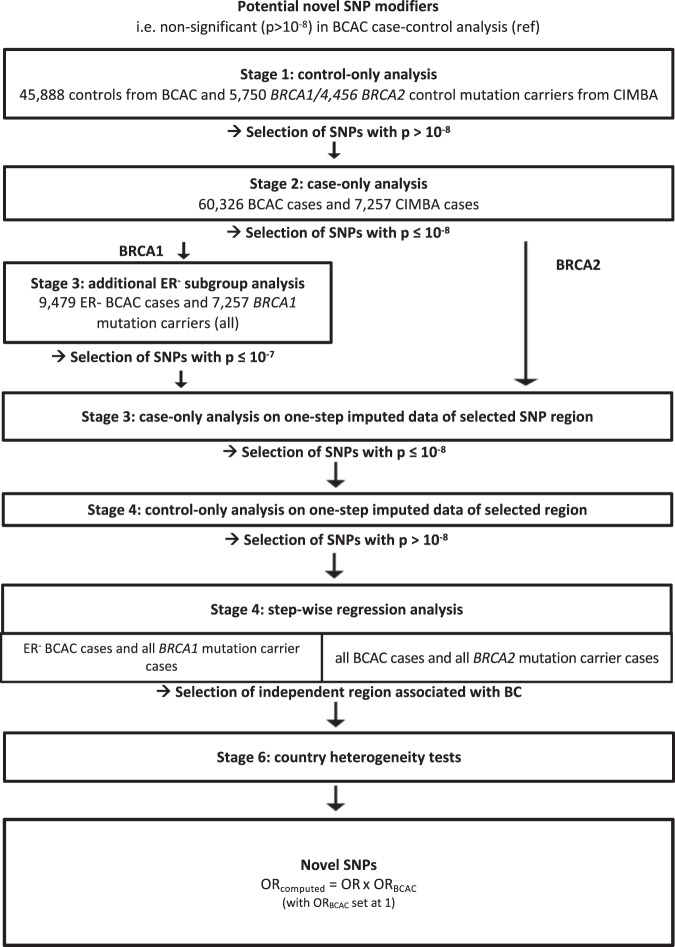
Analytical process for identifying novel modifiers. Strategy followed for identifying potentially novel SNP modifier.

The analytical process for assessing interactions with known BC susceptibility SNP is summarised in Fig. 3 and for the detection of novel modifiers in Fig. 4.

### Independence of SNP frequency with mutation carrier status

Under a case-only study design, it is important to establish independence between the SNPs and *BRCA1* or *BRCA2* mutation carrier status^53^. This was assessed a genome-wide level using a control-only analysis which included controls from BCAC and unaffected mutation carriers from CIMBA with SNP data imputed based on the 1,000 genomes project. Genotypes had been imputed separately by each consortium^7,50^. In the analysis of *BRCA1* mutation carriers, 2,164 SNPs were excluded because they were located in or within 500 kb of *BRCA1*. 2,070 SNPs were excluded from further analyses because they showed associations at *p* < 10^−8^ with *BRCA1* mutation carrier status in the control-only analysis (2,012 SNPs located on chromosome 17 and 58 on other chromosomes). In the analysis of *BRCA2* mutation carriers, 2,947 SNPs were excluded because they were located in or within 500 kb of *BRCA2*. A further 626 SNPs were excluded from further analyses because they were found to be associated with *BRCA2* mutation carrier status in the control-only analysis (566 SNPs on chromosome 13, and 60 on other chromosomes). A total of 9,068,301 SNPs remained for the *BRCA1*case-only association analysis and 9,043,830 SNPs for the *BRCA2*case-only analysis.

### Interactions with known BC susceptibility SNPs

Based on published data, 179 SNPs were considered as established BC susceptibility SNPs (Fig. 3); 158 SNPs were associated with overall BC risk^35^ and 21 additional SNPs were found to be associated through studies in ER-negative breast cancer^48^ (see Supplementary Table 11 in Milne et al.^48^). One of the 158 SNPs, rs11571833 located within *BRCA2* was excluded from the BRCA2 analysis. The detailed results are shown in Supplementary Data 1–3.

For *BRCA1* mutation carriers, previous studies have demonstrated heterogeneity in the associations of the SNPs with ER-positive and ER-negative breast cancer^35^. Since *BRCA1* mutation carriers develop primarily ER-negative BC, to comprehensively assess the evidence of interaction with *BRCA1* mutation status, we followed a two-step process; we first assessed the associations using all BC cases from BCAC and then we restricted the comparison to BCAC ER-negative BC cases. Of the 158 SNPs^35^, 59 were associated with *BRCA1* mutation carrier status when compared to all BC cases (*P* < 0.05, Supplementary Data 1). However, after adjusting for multiple testing, only four of these SNPs were associated (*P* < 2.7 × 10^−4^) and also showed evidence of association (*P* < 0.05) when compared with ER-negative BC cases (Table 1). Two additional SNPs on chromosome 1 and 6 (chr1_10566215_A_G and rs17529111) were associated at *P* < 2.7 × 10^−4^ with *BRCA1* mutation status only when compared with ER-negative BCAC cases. The OR estimates for association with *BRCA1* mutation status for these six SNPs were similar under both case-only analyses (all BC and ER-negative BC cases analyses) and varied from 0.85 to 1.07, suggesting that the magnitude of their associations with BC risk for *BRCA1* mutation carriers differs from that observed in the general population. For the other 152 SNPs, there was no evidence of association with *BRCA1* mutation status when compared against the ER-negative BC cases from BCAC (Supplementary Data 1), suggesting that the OR estimated using case-control data from BCAC are also applicable to *BRCA1* mutation carriers.

**Table 1:** Known BC susceptibility SNPs demonstrating associations in the BRCA1 case-only analysis.

Location	SNP name^a^	Chr^b^	Position^c^	Nearest gene	Estimated effect allele	Referent allele	Frequency^d^	*r*^2^	OR^e^	*P*^f^	OR_ER¯_^g^	*P*_ER-_^h^	OR_BCAC_^i^	*P*_BCAC_^j^	OR_computed_^k^	Variation in risk^l^
All BC SNPs																
1p22.3	rs17426269	1	88156923	*–*	A	G	0.16	1	0.90	2.70e^−04^	0.92	4.22e^−02^	1.05	1.70e^−04^	0.95	IOD
8q24.21	rs13281615	8	128355618	*–*	G	A	0.43	1	0.91	1.20e^−05^	0.94	4.14e^−02^	1.11	5.00e^−28^	1.01	TT1
10q22.3	chr10_80841148_C_T	10	80841148	*ZMZ1*	T	C	0.40	1	0.91	2.20e^−06^	0.91	1.01e^−03^	0.93	1.10e^−14^	0.84	ISD
16q12.1	chr16_52599188_C_T	16	52599188	*TOX3*	T	C	0.29	1	0.85	1.80e^−13^	0.91	2.80e^−03^	1.23	7.00e^−88^	1.04	TT1
ER^-^ BC SNPs																
1p36.22	chr1_10566215_A_G	1	10566215	*PEX14*	G	A	0.32	1	1.07	1.30e^−03^	1.12	1.10e^−04^	0.94	1.80e^−09^	1.05	TT1
6q14.1	rs17529111	6	82128386	*–*	C	T	0.23	0.96	0.92	7.70e^−04^	0.86	1.96e^−05^	1.02	4.20e^−02^	0.88	IOD
8p23.3	rs66823261	8	170692	*RPL23AP53*	C	T	0.23	0.92	–	–	0.88	2.37e^−04^	1.09	5.09e^−09^	0.96	TT1

Considering SNPs with known BC (Michailidou et al.)^35^ and ER-negative-specific BC (Milne et al.)^48^ associations in the general population.

All BC SNPs: SNPs associated with all BC in the general population.

*N* = 67,469 breast cancer cases (60,212 BCAC cases and 7,257 *BRCA1* mutation carrier cases).

ER^-^ BC SNPs: SNPs associated with ER-negative BC in the general population.

*N* = 16,736 breast cancer cases (9,479 BCAC ER^-^ cases and 7,257 *BRCA1* mutation carrier cases).

*TT1* tends to 1, *ISD* increase in same direction, *IOD* increase in opposite direction.

^a^After allowing for multiple testing, α* = 2.7 × 10^−4^.

^b^Chromosome.

^c^Build 37 positions.

^d^Frequency of the allele for which effect is estimated in BCAC cases (OncoArray dataset).

^e^Per allele odds ratio estimated in the case-only analysis.

^f^*p*-value in the case-only analysis (after allowing for multiple testing, *p* = 2.7 × 10^−4^).

^g^Per allele odds-ratio estimated in the case-only ER-negative subgroup analysis. OR values were computed from a two sided logistic regression using a 1 degree freedom likelihood ratio test (1 df lrtest) adjusted for age at BC diagnosis, country and the first four principal components.

^h^*p*-value in the case-only ER-negative subgroup analysis.

^i^Per allele odds-ratio estimated in BCAC (Michailidou et al.)^35^, except for * (Milne et al.)^48^.

^j^*p*-value in BCAC (Michailidou et al.)^35^, except for * (Milne et al.)^48^. For SNPs with *P*_BCAC_ > 10^−8^, significance was attained in merging data of Oncoarray, iCOGS and 11 different breast cancer GWAS in Michailidou et al.^35^ or Milne et al.^48^.

^k^Per allele computed odds-ratio (OR × OR_BCAC_).

^l^Compared with Michailidou et al’s OR estimates^35^.

Among the 21 ER-negative SNPs reported in Milne et al.^48^, only one (rs66823261) demonstrated significant evidence of association in the ER-negative case-only analysis (OR = 0.88, *p* < 2.7 × 10^−4^) (Table 1 and Supplementary Data 2). For the 20 other showing no association, the ORs estimated in Milne et al.^48^ would be applicable to *BRCA1* mutation carriers.

To estimate the association of the seven significant SNPs with BC for *BRCA1* mutation carriers (OR_computed_), the OR estimated using case-control data from BCAC (OR_BCAC_) was multiplied by the OR estimated using the case-only analysis (OR). For three SNPs, rs17426269, chr10_80841148_C_T and rs17529111, the magnitude of the association with BC for *BRCA1* mutation carriers was greater than that in the general population (OR_BCAC_) and for two of these three, the OR_computed_ was in the opposite direction than the OR_BCAC_ (Table 1). For the four other SNPs (rs13281615, chr16_52599188_C_T, chr1_10566215_A_G and rs66823261), the estimated interaction OR resulted in the OR for associations with *BRCA1* BC risk being closer to 1 (Table 1).

Among the remaining 172 SNPs (152 + 20) that showed no associations with *BRCA1* mutation status, the estimated OR_computed_ was smaller (i.e., closer to 1) than those estimated in the general population (OR_BCAC_) for 146 SNPs  (85%, Supplementary Data 1 and 2). Based on the analysis of ER^− ^tumours, the proportion of SNPs for which the OR_computed_ was closer to 1 than the OR_BCAC_ estimates was 59% (Supplementary Data 1 and 2).

For *BRCA2* mutation carriers, among the 157 SNPs known to be associated with BC risk in the general population, 43 were associated with *BRCA2* mutation carrier status at *P* < 0.05 in the case-only analysis that included all BCAC BC cases (Supplementary Data 3). However, only three SNPs (rs62355902, rs10759243 and chr22_40876234_C_T) showed associations after adjusting for multiple testing (*P* < 2.7 × 10^−4^) with OR estimates in the range of 0.88 to 0.89 (Table 2).

**Table 2:** Known BC susceptibility SNPs demonstrating associations in the BRCA2 case-only analysis.

Location	SNP name^a^	Chr^b^	Position^c^	Nearest gene	Estimated effect allele	Referent allele	Frequency^d^	*r*^2^	OR^e^	*P*^f^	OR_BCAC_^g^	*P*_BCAC_^h^	OR_computed_^i^	Variation in risk^j^
5q11.2	rs62355902	5	56053723	*MAP3K1*	T	A	0.18	0.98	0.89	1.10e^−04^	1.18	8.50e^−42^	1.05	TT1
9q31.2	rs10759243	9	110306115	*RP11-438P9.2*	A	C	0.31	1	0.89	4.60e^−06^	1.06	4.20e^−10^	0.95	TT1
22q13.1	chr22_40876234_C_T	22	40876234	*MKL1*	C	T	0.11	1	0.88	2.8e^−04^	1.12	5.70e^−16^	0.98	TT1

*N* = 62,822 breast cancer cases (57,725 BCAC cases and 5,097 *BRCA2* mutation carrier cases).

Considering SNPs with known BC (Michailidou et al.)^35^ associations in the general population.

*TT1* tends to 1, *ISD* increase in same direction, *IOD* increase in opposite direction.

^a^After allowing for multiple testing, α* = 2.7 × 10^−4^.

^b^Chromosome.

^c^Build 37 position.

^d^Frequency of the allele for which effect is estimated in BCAC cases (OncoArray dataset).

^e^Per allele odds ratio estimated in the case-only analysis. OR values were computed from a two sided logistic regression using a 1 df lrtest adjusted for age at BC diagnosis, country and the first four principal components.

^f^*p*-value in the case-only analysis (after allowing for multiple testing, p* = 2.7 × 10^−4^).

^g^Per allele odds-ratio estimated in BCAC (Michailidou et al.)^35^.

^h^*p*-value in BCAC (Michailidou et al.)^35^. For SNPs with *P*_BCAC_ > 10^−8^, significance was attained in merging data of Oncoarray, iCOGS and 11 different breast cancer GWAS in Michailidou et al.^35^.

^i^Per allele computed odds-ratio (OR × OR_BCAC_).

^j^Compared with Michailidou et al’s OR estimates^35^.

For these three SNPs, the observed interaction resulted in the magnitude of association with BC risk for *BRCA2* mutation carriers (OR_computed_) being closer to 1 (Table 2).

Of  the 154 SNPs that showed no significant associations with *BRCA2* mutation status, 79% had ORs of BC for *BRCA2* mutation carriers (OR_computed_) that were closer to 1 when compared to the ORs estimated using data in the general population (OR_BCAC_) (Supplementary Data 3).

### Novel SNP modifiers

To identify novel SNPs that modify BC risks for *BRCA1* and *BRCA2* mutation carriers, we used a case-only design to investigate the associations of SNPs that had not been previously shown to be associated with BC in the general population (Fig. 4).

For *BRCA1* mutation carriers, a total of 924 SNPs showed associations at *P* < 10^−8^ in all BC case-only analysis. To ensure that none of these associations are driven by differences in the distribution of ER-positive and ER-negative tumours in BCAC cases, an intermediate step was applied, in which we re-analysed the associations after restricting the BCAC data to only ER-negative cases. 220 of these SNPs remained significant at *P* < 10^−7^ located in 11 distinct genomic regions. SNPs were considered to belong to the same region if they were located within 500 kb of each other.

To ensure that none of these associations was driven by differences in the genotype imputation in the BCAC and CIMBA data (which had been carried out separately), all the SNPs in these 11 distinct genomic regions were re-imputed in the BCAC and CIMBA samples jointly and the associations for all SNPs in the regions were re-assessed in the control-only and case-only analyses. After the exclusion of 614 SNPs (613 on chromosome 17) that showed associations in the control-only analysis, 71 SNPs in two regions remained significant at *P* < 10^–8^ (Supplementary Data 4) in the case-only analyses including all BCAC cases. None of these SNPs had been previously reported in GWAS in the general population (*p*-values of association ranged from 0.51 to 5.9 × 10^−5^ with effect sizes in the range 0.96–1.04 in BCAC case-control analyses)^35,48^. A forward step-wise regression analysis within each of these two regions (restricted to the SNPs exhibiting associations at *p* < 10^−8^) starting with the most significant SNP and adding sequentially the other SNPs, identified a set of four conditionally independent SNPs (top SNPs) (Table 3): all SNPs were imputed, with *r*^2^ > 0.5, and had minor allele frequency (MAF) > 10%. Three of the top SNPs are located in 17q21.2. rs58117746 is an insertion of 16 bp within an exon of *KRTAP4-5* leading to a frameshift of the amino acid sequence. rs5820435 and rs11079012 are both intronic and located in *LEPREL4* (also named *P3H4*) and *JUP*, respectively, while rs80221606 is intronic and located in 11p11.2, within *CELF1*. The OR estimates of these four top SNPs ranged from 0.78 to 1.22. All showed evidence of heterogeneity in the OR by country (*P* < 0.05) (Table 3); however, in a leave-one-out analysis, in which each country was left out in turn, the overall associations remained similar (Supplementary Fig. 1 and 2) suggesting that no individual country had a big impact on the observed associations.

**Table 3:** List of potential novel SNP modifiers associated in the case-only analysis for *BRCA1* mutation carriers.

Location	SNP name^a^	Chr^b^	Position^c^	Nearest gene	Localisation	Estimated effect allele	Referent allele	*r*² ^d^	Frequency^e^	OR^f^	*P*^g^	OR_ER_^h^	*P*_ER¯_^i^	HR_CIMBA_^j^	*P*_CIMBA_^k^	OR_BCAC_^l^	*P*_BCAC_^m^	*P*_het_^n^	Target gene^o^
11p11.2	rs80221606	11	47560211	*CELF1*	Intronic	AT	A	0.76	0.10	0.78	1.12e^−10^	0.76	6.36e^−07^	0.98	7.60e^−01^	1.04	0.01	1.39e^−03^	Level 2
17q21.2	rs58117746	17	39305775	*KRTAP4-5*	Pepshift	TGGCAGCAGCTGGGGC	T	0.60	0.39	1.18	4.33e^−10^	1.15	7.71e^−05^	1.05	2.20e^−02^	1.02	0.26	4.60e^−04^	–
17q21.2	rs5820435	17	39961558	*LEPREL4*	Intronic	A	C	0.51	0.45	0.82	9.55e^−12^	0.85	7.71e^−05^	1.01	9.00e^−01^	1.02	0.07	1.06e^−08^	–
17q21.2	rs11079012	17	39912880	*JUP*	Intronic	G	C	0.66	0.31	1.17	7.06e^−09^	1.18	2.35e^−05^	0.98	3.10e^−01^	1.01	0.51	1.15e^−07^	Level 2

*N* = 67,469 breast cancer cases (60,212 BCAC cases and 7,257 *BRCA1* mutation carrier cases)

^a^The most significant SNP of each region after allowing for multiple testing, α* = 10^−8^

^b^Chromosome.

^c^Build 37 position.

^d^Imputation accuracy.

^e^Frequency of the allele for which effect is estimated in BCAC cases (OncoArray dataset).

^f^Per allele odds ratio estimated in the case-only analysis. OR values were computed from a two sided logistic regression using a 1 df lrtest adjusted for age at BC diagnosis, country and the first four principal components.

^g^*p*-value in the case-only analysis.

^h^Per allele odds-ratio estimated in the case-only ER-negative subgroup analysis.

^i^*p*-value in the case-only ER-negative subgroup analysis.

^j^Per allele hazard ratio estimated in CIMBA cohort analysis.

^k^*p*-value found in CIMBA cohort analysis.

^l^Per allele odds-ratio estimated in BCAC (Michailidou et al.)^35^.

^m^*p*-value in BCAC (Michailidou et al.)^35^. For SNPs with *P*_BCAC_ > 10^−8^, significance was attained in merging data of Oncoarray, iCOGS and 11 different breast cancer GWAS in Michailidou et al.^35^.

^n^*p*-value of the heterogeneity test by country.

^o^INQUISIT score level: 1 = most functional evidence supporting a potential link between CCVs and target gene.

For *BRCA2* mutation carriers, the case-only analysis identified 273 SNPs, located across 22 regions, with evidence of association at *P* < 10^−8^. After the joint re-imputation of the SNPs in these 22 regions, only 102 SNPs located in four regions (2p14, 13q13.1, and 13q13.2) remained associated at *P* < 10^–8^ (Supplementary Data 5). The step-wise regression analysis suggested that associations in each of the four regions were driven by a single variant (top SNPs) (Table 4). All four variants were imputed (with *r*^2^ > 0.5) and had MAF higher than 5%. At 2p14, rs12470785 (*r*^2^ = 0.98) is within an intron of *ETAA1*. At 13q13.1, rs79183898 (*r*^2^ = 0.84) is located between *B3GALTL* and *RXFP2* and rs736596 (*r*^2^ = 0.66) is within an intron of *STARD13*. At 13q13.2, rs4943263 (*r*^2^ = 0.99) is located between *RP11*-*266E6.3* and *RP11-307O13.1*. None of these SNPs had been previously reported to be associated with BC risk in BCAC studies in the general population (*p*-values from 0.01 to 0.90 in BCAC case-control analyses)^35,48^. The OR estimates of these four SNPs ranged from 0.85 to 1.37. All showed evidence of heterogeneity in the OR by country at *p* = 0.05 (Table 4). In the leave-one-country-out sensitivity analysis the two intergenic SNPs, rs79183898 and rs736596 were no longer significant at *P* < 10^−4^ when studies from the USA were excluded from the analysis and the OR estimates were substantially attenuated (Supplementary Figs. 3 and 4).

**Table 4:** List of potential novel SNP modifiers associated in the case-only analysis for *BRCA2* mutation carriers.

Location	SNP name^a^	Chr^b^	Position^c^	Nearest gene	Localisation	Estimated effect allele	Referent allele	*r*² ^d^	Frequency^e^	OR^f^	*P*^g^	HR_CIMBA_^h^	P_CIMBA_^i^	OR_BCAC_^j^	*P*_BCAC_^k^	*P*_HET_^l^	Target gene^m^
2p14	rs12470785	2	67634003	*ETAA1*	Intron	G	A	0.98	0.30	0.84	2.83e^−11^	0.89	1.69e^−05^	0.98	0.03	2.18e^−07^	Level 2
13q13.1	rs79183898	13	32221794	*B3GALTL - RXFP2*	Intergenic	A	T	0.84	0.07	1.33	2.88e^−10^	1.04	3.55e^−01^	1.01	0.54	1.12e^−08^	–
13q13.1	rs736596	13	33776506	*STARD13*	Intron	T	G	0.66	0.09	1.37	3.44e^−12^	0.94	2.54e^−01^	0.98	0.45	4.99e^−11^	Level 1
13q13.2	rs4943263	13	35376357	*RP11-266E6.3 - RP11-307O13.1*	Intergenic	T	C	0.99	0.27	1.17	8.33e^−11^	1.01	9.83e^−01^	1.00	0.47	6.94e^−03^	Level 2

*N* = 62,822 breast cancer cases (57,725 BCAC cases and 5,097 *BRCA2* mutation carrier cases).

^a^The most significant SNP of each region after allowing for multiple testing, α* = 10^−8^

^b^Chromosome.

^c^Build 37 position.

^d^Imputation accuracy.

^e^Frequency of the allele for which effect is estimated in BCAC cases (OncoArray dataset).

^f^Per allele odds ratio estimated in the case-only analysis. OR values were computed from a two sided logistic regression using a 1 df lrtest adjusted for age at BC diagnosis, country and the first four principal components.

^g^*p*-value in the case-only analysis.

^h^Per allele hazard ratio estimated in CIMBA cohort analysis.

^i^*p*-value found in CIMBA cohort analysis.

^j^Per allele odds-ratio estimated in BCAC (Michailidou et al.)^35^.

^k^*p*-value in BCAC (Michailidou et al. 2017). For SNPs with *P*_BCAC_ > 10^−8^, significance was attained in merging data of Oncoarray, iCOGS and 11 different breast cancer GWAS in Michailidou et al.^35^.

^l^*p*-value of the heterogeneity test by country.

^m^INQUISIT score level: 1 = most functional evidence supporting potential link between CCVs and target gene.

### In silico analyses on credible causal variants (CCV)

In order to determine the likely target genes of each region of the eight novel mutation carriers’ BC risk-associated SNPs, we first defined credible set of SNPs candidates to be causal (credible causal variants [CCVs]) (see “Methods”).

Sets of CCVs were sought for the two regions found in the previous step-wise analyses to be associated with risk in *BRCA1* mutation carriers. In the region located at 11p11.2, only one signal composed of 74 CCVs was found (Table 5). All these 74 CCVs were imputed with a *r*^2^ higher than 0.92 (Supplementary Data 6). In the region located in 17q21.2, we found nine signals which contained from one to 13 CCVs (Table 5). Two of these CCVs were genotyped and the others had an *r*^2^ between 0.50 and 0.98 (Supplementary Data 6).

**Table 5:** List of most significant SNPs in the CCV analysis for *BRCA1* mutation carriers.

Fine mapping region^a^	Signal^b^	#CCV^c^	Location	SNP name^d^	Chr^e^	Position^f^	Nearest gene	Localisation	Estimated effect allele	Referent Allele	Frequency ^g^	*r*² ^h^	*P*^i^	OR^j^	*P*_ER_^k^	OR^l^	P_CIMBA_^m^	HR_CIMBA_^n^
chr11:46773616-47773616	1	74	11p11.2	rs60882887	11	47475675	*RAPSN, CELF1*	Intergenic	A	G	0.14	0.95	2.20e^−10^	0.82	3.20e^−06^	0.82	7.00e^−01^	0.99
chr17:39141815-40141815	1	2	17q21.2	rs5820435	17	39961558	*LEPREL4*	Intronic	A	C	0.45	0.51	1.10e^−11^	0.82	2.80e^−05^	0.85	9.10e^−01^	1.00
	2	2	17q21.2	rs7222250	17	39938469	*JUP*	Intronic	C	T	0.44	0.66	5.50e^−14^	1.23	3.90e^−07^	1.20	8.70e^−01^	1.00
	3	6	17q21.2	rs9901834	17	39926811	*JUP*	Intronic	A	G	0.10	0.55	7.20e^−10^	0.72	3.90e^−06^	0.72	7.40e^−01^	1.02
	4	3	17q21.2	rs58117746	17	39305775	*KRTAP4-5*	Intronic	TGGCAGCAGCTGGGGC	T	0.39	0.60	5.50e^−09^	1.17	4.60e^−04^	1.13	2.20e^−02^	1.06
	5	13	17q21.2	rs2239711	17	39633317	*KRT35*	Intronic	A	G	0.29	0.93	4.90e^−11^	0.85	2.90e^−04^	0.88	5.00e^−01^	0.98
	6	4	17q21.2	rs10708222	17	40137437	*DNAJC7*	Intronic	T	TA	0.17	0.60	8.40e^−07^	1.18	6.10e^−04^	1.17	2.28e^−01^	0.95
	7	4	17q21.2	rs41283425	17	39925713	*JUP*	Intronic	T	C	0.06	0.54	4.30e^−07^	0.73	1.30e^−05^	0.69	4.82e^−01^	0.95
	8	15	17q21.2	rs56291217	17	39858199	*JUP*	Intronic	AT	A	0.44	0.76	6.70e^−08^	0.88	1.20e^−06^	0.85	4.06e^−01^	1.03
	9	1	17q21.2	rs111637825	17	40134782	*DNAJC7*	Intronic	A	G	0.06	0.89	3.60e^−07^	0.74	3.50e^−04^	0.75	4.47e^−01^	0.96

*N* = 67,469 breast cancer cases (60,212 BCAC cases and 7,257 BRCA1 mutation carrier cases).

^a^Significant region in the main analysis used to look for credible causal variants (CCV).

^b^Signal number (the first one corresponds to the CCV set without any adjustment and the following are those with adjustment on each most significant SNP of the previous signals).

^c^Number of credible causal variants at each signal (SNP with *p*-value at 2 order of magnitude of the most significant one).

^d^The most significant SNP after adjustment on the most significant SNPs of the previous signals (except for these of the signal 1).

^e^Chromosome.

^f^Build 37 position.

^g^Frequency of the allele for which effect is estimated in BCAC cases (OncoArray dataset).

^h^Imputation accuracy.

^i^*p*-value in the case-only analysis after adjustment on the most significant SNPs of the previous signals (except for these of the signal 1).

^j^Per allele odds ratio estimated in the case-only analysis. OR values were computed from a two sided logistic regression using a 1df lrtest adjusted for age at BC diagnosis, country, the first four principal components and the most significant SNPs of the previous signals (except for these of the signal 1).

^k^*P*-value in the case-only analysis restricted to ER-negative BCAC cases and after adjustment with the most significant SNP of the previous signals (except for these of the signal 1).

^l^Per allele odds ratio estimated in the case-only analysis restricted to ER-negative BCAC cases and after adjustment with the most significant SNP of the previous signals (except for these of the signal 1).

^m^*p*-value found in CIMBA cohort analysis.

^n^ Per allele hazard ratio estimated in CIMBA cohort analysis.

We used INQUISIT^35,54^ to prioritize target genes by intersecting each CCV with publicly available annotation data from breast cells and tissues (see “Methods”). The results for *BRCA1* mutation carriers are summarized in Supplementary Data 7. For *BRCA1* mutation carriers, we predicted 38 unique target genes for six of the 10 independent signals. Seven target genes in two regions (*MTCH2, MADD*, *PSMC3, RP11-750H9.5, SLC39A13, SPI1*, and *EIF1*) were predicted with high confidence (designated Level 1, scoring range between Level 1 [highest confidence] to Level 3 [lowest confidence]). All seven Level 1 genes were predicted to be distally regulated by CCVs.

Similarly, sets of CCVs were sought from the four regions found in the previous step-wise analyses to be associated with risk in *BRCA2* mutation carriers. A total of 17 signals were found. One signal composed of 78 CCVs was found in the region located at 2p14 (Table 6). One CCV was genotyped and the others were imputed with *r*^2^ between 0.95 and 0.99 (Supplementary Data 8). Twelve signals were found from the two regions previously found in 13q13.1 which contained from one to 46 CCVs. The analysis in the region of rs79183898 in 13q13.1 found three signals out of the 12, which are located in 13q12.3 (with top SNPs: rs71434801, rs77197167, rs114300732). Finally, four signals in the previously identified region located in 13q13.2 containing from three to 40 CCVs were found. Among all CCVs, 11 are genotyped and the imputed ones have an *r*^2^ higher than 0.58 (Table 6 and Supplementary Data 8).

**Table 6:** List of most significant SNPs in the CCV analysis for *BRCA2* mutation carriers.

Fine mapping region^a^	Signal^b^	#CCV^c^	Location	SNP name^d^	Chr^e^	Position^f^	Nearest gene	Localisation	Estimated effect allele	Referent allele	Frequency ^g^	*r*² ^h^	*P*^i^	OR^j^	*P*_CIMBA_^k^	HR_CIMBA_^l^
chr2:67099466-68099466	1	78	2p14	rs12470785	2	67634003	*ETAA1*	Intronic	G	A	0.30	0.98	4.20e^−11^	0.85	7.70e^−05^	0.89
chr13:31015494-32515494	1	8	13q13.1	rs79183898	13	32221794	*B3GALTL, RXFP2*	Intergenic	A	T	0.07	0.84	1.10e^−10^	1.33	3.60e^−01^	1.04
	2	23	13q12.3	rs71434801	13	31249461	*USPL1, ALOX5AP*	Intergenic	G	C	0.13	0.76	3.40e^−08^	1.22	8.40e^−01^	0.99
	3	35	13q12.3	rs77197167	13	31693513	*WDR95P, HSPH1*	Intergenic	C	T	0.09	0.76	1.80e^−07^	1.25	4.00e^−01^	1.04
	4	7	13q12.3	rs114300732	13	31662987	*WDR95P*	Intronic	T	C	0.07	0.90	1.70e^−08^	0.67	8.80e^−02^	1.09
	5	12	13q13.1	13:32231513:CAA:C	13	32231513	*B3GALTL, RXFP2*	Intergenic	CAA	C	0.25	0.92	8.40e^−07^	0.86	1.70e^−02^	1.08
	6	6	13q13.1	rs1623189	13	32232683	*B3GALTL, RXFP2*	Intergenic	G	T	0.26	0.95	1.30e^−31^	2.70	6.60e^−01^	1.01
chr13:33395975-34395975	1	1	13q13.1	rs736596	13	33776506	*STARD13*	Intronic	T	G	0.09	0.66	1.20e^−12^	1.37	2.50e^−01^	0.95
	2	1	13q13.1	rs77889880	13	33776161	*STARD13*	Intronic	T	A	0.10	0.80	3.00e^−21^	0.51	1.90e^−02^	1.12
	3	1	13q13.1	rs67776313	13	33934343	*RP11-141M1.3*	Intronic	A	AT	0.33	0.70	7.70e^−12^	0.81	4.60e^−01^	0.98
	4	42	13q13.1	rs71196514	13	33800572	*STARD13*	Intronic	C	CT	0.38	0.67	1.00e^−07^	0.86	6.20e^−01^	1.01
	5	52	13q13.1	rs2555605	13	33833810	*STARD13*	Intronic	C	T	0.36	1.00	4.60e^−08^	0.87	2.00e^−01^	1.03
	6	46	13q13.1	rs74796280	13	33700860	*STARD13*	Intronic	C	A	0.06	0.96	4.70e^−07^	0.77	3.10e^−02^	0.89
chr13:34793902-35793902	1	18	13q13.2	rs4943263	13	35376357	*RP11-266E6.3, RP11-307O13.1*	intergenic	T	C	0.27	0.99	6.30e^−11^	1.18	9.80e^−01^	1.00
	2	3	13q13.2	rs2202781	13	35292372	*RP11-266E6.3, RP11-307O13.1*	Intergenic	G	A	0.24	0.93	3.10e^−11^	1.20	6.00e^−01^	0.98
	3	40	13q13.2	rs55675572	13	35315594	*RP11-266E6.3, RP11-307O13.1*	intergenic	A	T	0.40	0.77	5.60e^−08^	0.86	7.50e^−01^	0.99
	4	21	13q13.2	rs17755120	13	35270340	*RP11-266E6.3, RP11-307O13.1*	intergenic	T	A	0.20	0.98	6.30e^−07^	0.76	4.80e^−01^	0.98

*N* = 62,822 breast cancer cases (57,725 BCAC cases and 5,097 *BRCA2* mutation carrier cases).

^a^Significant region in the main analysis used to look for credible causal variants (CCV).

^b^Signal number (the first one corresponds to the CCV set without any adjustment and the following are those with adjustment on each most significant SNP of the previous signals).

^c^Number of credible causal variants at each signal (SNP with *p*-value at 2 order of magnitude of the most significant one).

^d^The most significant SNP after adjustment on the most significant SNPs of the previous signals (except for these of the signal 1).

^e^Chromosome.

^f^Build 37 position.

^g^Frequency of the allele for which effect is estimated in BCAC cases (OncoArray dataset).

^h^Imputation accuracy.

^i^*p*-value in the case-only analysis after adjustment on the most significant SNPs of the previous signals (except for these of the signal 1).

^j^Per allele odds ratio estimated in the case-only analysis. OR values were computed from a two sided logistic regression using a 1df lrtest adjusted for age at BC diagnosis, country, the first four principal components and the most significant SNPs of the previous signals (except for these of the signal 1).

^k^*p*-value found in CIMBA cohort analysis.

^l^Per allele hazard ratio estimated in CIMBA cohort analysis.

For *BRCA2* mutation carriers, we predicted 24 unique target genes for 10 of the 17 independent signals, including one high confidence target gene, *STARD13* at chr13:33395975-34395975. *STARD13* was also predicted to be targeted by three independent signals. All results are presented in Supplementary Data 9.

## Discussion

To identify novel genetic modifiers of BC risk for *BRCA1* and *BRCA2* mutation carriers and to further clarify the effects of known BC susceptibility SNPs on BC risk for carriers, a novel case-only analysis strategy was used based on GWAS data from unselected BC cases in BCAC and mutation carriers with BC from CIMBA. This strategy provides increased statistical power for detecting new associations and for clarifying the risk associations of known BC susceptibility SNPs in mutation carriers^55^.

Of the 179 known BC susceptibility SNPs identified through GWAS in the general population^5–35^, only 10 showed evidence of interaction with *BRCA1* or *BRCA2* mutation carrier status after taking the tumour ER-status into account. None of these 10 SNPs was among the fifty SNPs previously shown to be associated with BC for mutation carriers^5,6,15,18,20,39–48^. However, 82% of all 179 known susceptibility SNPs showed a predicted OR point estimate for mutation carriers closer to 1 than that estimated in the general population. The effect sizes in the general population may be somewhat exaggerated as the BCAC dataset used here contributed to the discovery of most of the loci, although this effect is likely to be small as most loci are highly significant and the effects have been replicated in independent datasets^7^. Taken together, these results suggest that, while most SNPs associated with risk in the general population are associated with risk for mutation carriers, the average effect sizes for mutation carriers are smaller. These findings are in line with previous results by Kuchenbaecker et al.^49^ and suggest that a PRS built using data from the general population will have a smaller effect size for *BRCA1/2* mutation carriers.

For 10 SNPs, an interaction was observed with *BRCA1* or *BRCA2* mutation carrier status, suggesting that these SNPs have different effect sizes in *BRCA1* or *BRCA2* mutation carriers compared to the general population (seven for *BRCA1* mutation carriers and three for *BRCA2* mutation carriers). Specifically, for seven SNPs the confidence intervals were consistent with no effect on BC risk for mutation carriers, one SNP was associated with a larger OR for mutation carriers compared to the general population and two were associated in the opposite direction to that observed in the general population. However, distinguishing between a smaller effect size for mutation carriers compared to the general population OR estimates and no association for mutation carriers is very challenging since, even with the large sample size here, it is not possible to estimate precisely the effect sizes for individual variants. Larger sample sizes will be required for this purpose. Determining the precise effects of the SNPs in *BRCA1* and *BRCA2* mutation carriers will provide insights for understanding the biological basis of cancer development associated with *BRCA1* and *BRCA2* mutations.

We also identified eight novel conditionally independent common SNPs associated with BC risk (four for *BRCA1* mutation carriers, four for *BRCA2* mutation carriers). These have not been reported in previous association studies^5,6,15,18,20,39–47^. The case-only OR estimates for these SNPs varied from 0.85 to 1.37 for *BRCA2* mutation carriers and from 0.78 to 1.22 for *BRCA1* mutation carriers. For five of these SNPs the estimated ORs from the case-only analysis results were in the same direction as the estimated HRs from previously reported GWAS using cohort analyses restricted in *BRCA1* and *BRCA2* mutation carriers in CIMBA^56^. Two of these five SNPs also demonstrated some evidence of association in mutation carriers (*p* = 2.2 × 10^−2^ for rs58117746 for *BRCA1* mutation carriers; and *p* = 7.7 × 10^−5^ for rs12470785 in *ETAA1* for *BRCA2* mutation carriers; Tables 3 and 4). For the remaining three variants, rs5820435 and rs11079012 at 17q21.2 and rs736596 at 13q13.1, the associations in *BRCA1* or *BRCA2* mutation carriers in the CIMBA data were not consistent with the observed interactions and might be artefactual. One possibility is that the associations with SNPs on 17q and 13q in *BRCA1* and *BRCA2* carriers respectively, reflect confounding due to linkage disequilibrium (LD) with specific mutations. Although we excluded variants with evidence of association in the control only analyses, it is possible that residual confounding due to specific mutations was still present.

Seven genes at a locus at 11p11.2 marked by rs60882887, were predicted with high confidence as targets, including *MADD*, *SP11* and *EIF1* which have previously been reported to be associated with BC biology^57–59^. However, no likely target genes were predicted at the 17q21.2 region. The lack of target gene predictions may be due to reliance on breast cell line data which does not represent the in vivo tissue of interest or due to the fact that the target transcripts are not annotated.

Only one gene, *STARD13*, was predicted as a potential target of SNPs at 13q13.1. This tumour suppressor gene has been previously implicated in metastasis, cell proliferation and development of BC^60^. However, rs736596, localized at 13q13.1, showed no association in CIMBA analyses and the association observed in our case-only analysis showed heterogeneity by country.

At the 2p14 locus, INQUISIT-predicted target genes included *ETAA1* with lower confidence. The OR estimates obtained in the case-only analysis for the SNPs located in this gene were consistent with the HR estimated in previously reported CIMBA analyses^56^. Moreover, around one hundred correlated SNPs, were associated with *BRCA2* mutation carrier status at *p* < 10^−8^, including the genotyped SNP chr2_67654113_C_T.

The validity of the case-only analysis as evidence of interaction relies on the assumption of independence between the mutation status and the SNPs under investigation^61^. Therefore, based on the control-only analyses, we excluded ~2,000 SNPs which were associated with *BRCA1* or *BRCA2* mutation carrier status and also showed an association with risk in the case-only analyses (Supplementary Fig. 5). While most of these associations are probably spurious, due to (intra- or inter-chromosomal) LD with *BRCA1* or *BRCA2* mutations, it is possible that some may reflect true associations and that the higher frequency in unaffected *BRCA1/2* may be because they are relatives of BC cases. These associations may warrant further evaluation using other study designs. A recent publication using data from the Framingham Heart Study suggested that interchromosomal LD can be caused by bio-genetic mechanisms possibly associated with favourable or unfavourable epistatic evolution^62^. SNPs for which no association with mutation carrier status was found at the significance level of 10^−8^ were assumed to be independent of the mutation status. However, this does not necessarily rule out residual LD between the novel SNPs on chromosomes 13 and 17 and *BRCA1* or *BRCA2* mutations. Therefore, the OR estimates for these SNPs might be biased and may further explain the lack of evidence of association in the CIMBA only analyses.

Our findings highlight the importance of imputation in GWAS. The imputed genome-wide genotype data used in the main case-only association analyses were based on carrying out the imputation separately for the BC cases from BCAC and CIMBA. We found that 28 out of the 33 regions associated with *BRCA1* or *BRCA2* mutation carrier status were no longer associated with risk after re-imputing all samples together. By re-imputing all the data together we ensured that the associations observed for the remaining regions are robust to potential differences in the imputation accuracy between the BCAC and CIMBA samples.

Under our analytical strategy, only the regions for which evidence of associated with BC risk was observed were re-imputed using all BCAC and CIMBA samples combined. This re-imputation was not done at genome-wide level due to computational constraints and this may have led to false-negative associations being excluded for further evaluation as potential novel modifiers. Future analyses should aim to analyse the genome-wide associations after the genome-wide re-imputation across the combined BCAC and CIMBA dataset. However, our approach using joint one-step imputation should have ensured that associations we report (all of which are common SNPs with imputation scores > 0.5) are not driven by inaccuracies in imputation.

Due to the recruitment of participants in CIMBA studies primarily through genetic counselling, the mean age at diagnosis of mutation carriers was 16 years younger than the BC cases participating in BCAC. Although all analyses were adjusted for age, the observed associations might be related to the ageing process instead of interactions with mutation carrier status. Another source of bias could be related to the fact that there are 1.5 times more prevalent cases among CIMBA (68.1%) than BCAC (42.3%) with a delay between diagnosis and study recruitment of 6.83 years and 2.07 years respectively. An observed association might be due to a differential survival between CIMBA and BCAC cases. However, none of the identified SNPs has been found to be associated with BC survival^63^.

The majority (92.5%) of cases and controls in BCAC were not tested for *BRCA1/2* mutations at the time of enrolment, potentially leading to some attenuation in the interaction OR (as some BCAC cases will be carriers). However, most BCAC studies were population-based case-control studies and the proportion of cases and controls that carry pathogenic *BRCA1/2* mutations will be small (<5%), hence any attenuation is likely to be negligible.

Despite heterogeneity in the interaction ORs by country for some SNPs, results were generally robust to the exclusion of each country sequentially except, for two SNPs (rs79183898 and rs736596) found associated with *BRCA2* mutation carrier status; for these, the association seemed to be driven by data from the USA. For the other SNPs, the observed heterogeneity may be due to random error, given the relatively small sample sizes of each country. However, if these differences are real, future PRS for *BRCA1* and *BRCA2* carriers should consider the country-specific differences.

This is the first analysis of genetic modifiers of BC risk that investigated the differences in the association of common genetic variants with BC risk in the general population and in women with *BRCA1* or *BRCA2* mutations. The inclusion of unselected BC cases resulted in increased sample size and hence a gain in statistical power for identifying novel SNPs. These represent the largest currently available datasets, but it is important to replicate these observations in independent samples. This should be possible through the ongoing CONFLUENCE (https://dceg.cancer.gov/research/cancer-types/breast-cancer/confluence-project) large-scale genotyping experiment. More detailed fine mapping and functional analysis will be required to elucidate the role of the novel variants identified in BC development for *BRCA1* and *BRCA2* mutation carriers. Our findings should contribute to the improved performance of BC PRS for absolute risk prediction for *BRCA1* and *BRCA2* mutation carriers, which will help inform decisions on the best timing for risk-reducing surgery, risk reduction medication, or the start of surveillance.

## Methods

### Study sample

We used data from two international consortia, BCAC^64^ and CIMBA^56^. BCAC included data from 108 studies of BC from 33 countries in North America, Europe and Australia, the majority (88%) of which were case-control studies. The majority of BCAC cases/controls were not tested for *BRCA1/2* mutations at the time of enrolment. However, most studies were population-based, hence the proportion of cases and controls that carry pathogenic *BRCA1/2* mutations will be small. CIMBA participants were women with pathogenic mutations in *BRCA1* or *BRCA2*. All participants were at least 18 years old. The majority of mutation carriers were recruited through cancer genetics clinics and enroled into national or regional studies. Data were available on 30,500 *BRCA1* mutation carriers and 20,500 *BRCA2* mutation carriers from 77 studies in 32 countries. A total of 188,320 BC cases and 161,669 controls were available from both consortia. All studies provided information on disease status, age at diagnosis or at interview. Oestrogen receptor status was available for 72% of BCAC cases and 71% of CIMBA cases. All subjects provided written informed consent and participated in studies with protocols approved by ethics committees at each participating institution.

### Sample selection

BCAC cases were women diagnosed with BC^7^. To define disease status in CIMBA participants, women were censored at the first of the following events: age at BC diagnosis, age at ovarian cancer diagnosis, other cancer, bilateral prophylactic mastectomy or age at study recruitment. Subjects censored at a BC diagnosis were considered as cases.

A control-only analysis was carried out to test the independence between the SNPs and the *BRCA1* and *BRCA2* mutation carrier status. In BCAC, controls were defined as individuals unaffected by BC at study recruitment^35^. In CIMBA, participants were considered as controls if they were unaffected at recruitment.

Only women of European ancestry were included. To minimise the chance of observing spurious associations due to differences in the distribution of BC cases in the population by tumour characteristics (defined as unselected BC cases), 3,478 BCAC cases from four studies were excluded because they were included in clinical trials based on breast tumour characteristics as HER-2 receptor status (see Supplementary Table 2). Because all the analyses were adjusted for country, to ensure that the number of subjects in each country stratum was large enough, we excluded the CIMBA data from any country for which there were less than ten BC cases with *BRCA1* or *BRCA2* mutation. Consequently, data from Poland and Russia were excluded from the BRCA2 analyses (Supplementary Table 3). Finally, duplicate subjects between BCAC and CIMBA were excluded from the BCAC data (114 and 80 subjects from the BRCA1 and BRCA2 case-only analyses, respectively; eight subjects from control-only analyses).

A total of 60,212 BCAC cases and 7,257 *BRCA1* mutation carrier cases were available for the BRCA1 case-only analyses and 57,725 BCAC cases and 5097 *BRCA2* mutation carrier cases were available for the BRCA2 case-only analyses (Fig. 1). A total of 45,881 BCAC controls and 5,750 *BRCA1* mutation carrier controls were available for the BRCA1 control-only analyses and 43,549 BCAC controls and 4,456 *BRCA2* mutation carrier controls for the BRCA2 control-only analyses (Fig. 2).

### Genotype data

All the study samples were genotyped using the OncoArray Illumina beadchip^65^. The array includes a backbone of ~260,000 SNPs that provide genome-wide coverage of most common variants, together with markers of interest for breast and other cancers identified through GWAS, fine-mapping of known susceptibility regions, and other approaches^65^.

A standard genotype quality control process was followed for both the BCAC and CIMBA samples which have been described in detail elsewhere^35,48^. Briefly, this involved excluding SNPs located on chromosome Y; SNPs with call rates <95%; SNPs with MAF < 0.05 and call rate <98%; monomorphic SNPs; and SNPs for which evidence of departure from Hardy-Weinberg equilibrium was observed (*P* < 10^−7^ based on a country-stratified test).

Genotypes for ~21 million SNPs were imputed for all subjects using the 1000 Genomes Phase III data (released October 2014) as reference panel, as described previously^66^. Briefly, the number of reference haplotypes used as templates when imputing missing genotypes was fixed to 800 (-k_hap = 800). A two-stage imputation approach was used: phasing with SHAPEIT^67,68^ and imputation with IMPUTE2^69^ using 5 Mb non-overlapping intervals. Genotypes were imputed for all SNPs that were found polymorphic (MAF > 0.1%) in either European or Asian populations.

The genome-wide imputation process described above was carried out separately for the BCAC and CIMBA samples. However, this may potentially lead to spurious associations if there are differences in the quality of the imputation (measured using the imputation accuracy *r*² metric^70^) for a given SNP between the two datasets. To address this, a stringent approach was employed which involved including only SNPs for which the difference in *r*² between the BCAC and CIMBA SNP imputations (Δ*r*²) was minimal relative to their *r*² values. SNPs with *r*² > 0.9 in both BCAC and CIMBA were kept in the analyses only if Δ*r*² < 0.05; SNPs with 0.8 < *r*² ≤ 0.9 in both BCAC and CIMBA were kept if Δ*r*² < 0.02 and, SNPs with 0.5 < *r*² ≤ 0.8 in both BCAC and CIMBA were kept if Δ*r*² < 0.01. All SNPs with *r*² < 0.5 in either CIMBA or BCAC were excluded. Only SNPs with a MAF > 0.01 in BCAC cases were included.

Consequently, 9,072,535 SNPs were included in the BRCA1 analyses (402,336 genotyped and 8,670,199 imputed SNPs) and 9,047,403 SNPs in the BRCA2 analyses (402,397 genotyped and 8,645,006 imputed SNPs).

### Case-only and control-only analyses

The comparison of SNP frequency between CIMBA cases and BCAC cases (case-only analyses), or between unaffected CIMBA subjects and BCAC controls (control-only analyses), was performed using logistic regression adjusted for age at BC diagnosis in the case-only analyses and for age at interview for BCAC controls or at censure for CIMBA unaffected subjects in the control-only analyses, as well as for country and principal components (PCs) to account for population structure. Separate analyses were carried out for *BRCA1* and *BRCA2* mutation carriers. To define the number of PC for inclusion in the models, the principal component analysis was carried out using 35,858 uncorrelated genotyped SNPs on the OncoArray and purpose-written software (http://ccge.medschl.cam.ac.uk/software/pccalc/). The inflation statistic was calculated and converted to an equivalent statistic for a study of 1,000 subjects for each outcome (*λ*_1,000_) by adjusting for effective study size:

$$\lambda _{1,000} = \left( {\lambda - 1} \right)\left( {\frac{1}{n} + \frac{1}{m}} \right) \, * \, 500 + 1$$

where *n* and *m* are the numbers of BCAC and CIMBA subjects respectively. The models were adjusted with the first four PCs (*λ*_1,000_ with and without PCs in the model = 1.03 and 1.21, respectively) since additional PCs did not result in further reduction in the inflation of the test statistics.

### Strategy for determining significant associations

The analytical process is summarised in Figs. 3 and 4. A fundamental assumption when using a case-only design in this context is that the SNPs and mutation carrier status are independent^61^. To confirm independence, SNPs likely to be in linkage disequilibrium (LD) with *BRCA1* or *BRCA2* mutations, i.e., those located in or within 500 kb of either gene, were excluded. However, LD also exists between variants at long-distance on the same chromosome or even on a different chromosome (interchromosomal LD)^62,71^. Therefore, control-only analyses were performed to further exclude SNPs associated with mutation carrier status in unaffected women^72^, using a stringent statistical significance level of 10^−8^).

After excluding SNPs in LD or in interchromosomal LD with *BRCA1* or *BRCA2* mutations, case-only analyses were performed to assess the association between SNPs and *BRCA1* or *BRCA2* mutation carrier status. We considered two categories of SNPs depending on whether they had been previously found to be associated with BC in published BCAC studies^35,48^. For known BC susceptibility SNPs (Fig. 3) we used a significance threshold of 2.7 × 10^−4^ (applying Bonferroni correction to 179 tests) and for potential novel SNP modifier (Fig. 4) a stringent significance threshold of 10^−8^ was used.

Because *BRCA1*mutation-associated tumours are more often ER-negative than those in the general population^73^, a subsequent case-only analysis was performed restricting the BCAC cases to those with ER-negative disease. We used this strategy for two reasons. First, we wished to exclude associations driven by differences in the tumour ER-status distributions between *BRCA1* carriers and BCAC cases. Therefore, in the *BRCA1* analysis, SNPs were considered to be associated with mutation carrier status only if they were also associated in the ER-negative case-only analysis at a prior defined significance threshold of 10^−7^ for novel SNP modifiers (Fig. 4) and of 0.05 for the established BC susceptibility SNPs after a pre-selection at *P* < 2.7 × 10^−4^ in the *BRCA1* overall case-only analysis (Fig. 3). The second reason we applied this strategy was to identify novel SNP modifiers specific to *BRCA1*/ER-negative tumours that had not been detected in the overall analysis; for this we applied a significance threshold of 10^−8^.

To confirm that potentially novel associations in the case-only analysis were not driven by differences in the imputation accuracy between the CIMBA and BCAC data, each of the regions defined as ±500 kb around the associated SNP, were re-imputed for the combined CIMBA and BCAC samples. The more accurate one-stage imputation was carried out, using IMPUTE2 without pre-phasing. Associations with all the SNPs in the re-imputed regions were then re-evaluated using the control-only and case-only analytical approaches described above. Finally, we used a step-wise regression analysis using a significance threshold of 10^−8^ in order to determine whether associations with SNPs in the same region are independent and to define the conditionally independent SNPs (top SNPs).

Among the 179 established BC susceptibility SNPs, 107 were genotyped and 71 were imputed. As previously, although none of these 71 SNPs were excluded based on their Δ*r*², to exclude potentially spurious associations, regions around these 71 SNPs were re-imputed using the one-stage imputation applied to BCAC and CIMBA data combined, and before performing the control-only and case-only analyses.

### Determining the magnitude of association

For the potentially novel SNP modifiers the risk ratio of BC applicable to mutation carriers was assumed to be equal to the OR estimate from the case-only analysis (with the hypothesis that their relative risk equals 1 in the general population, given that none of them was found to be associated with BC in BCAC)^55^.

For the known BC susceptibility SNPs, a significant association in the case-only analysis implies that the magnitude of association is different for *BRCA1* or *BRCA2* mutation carriers than for the general population. Therefore, the risk ratio of BC for mutation carriers was computed as the product of OR × OR_BCAC_ where OR was obtained from the case-only analysis, and OR_BCAC_ was the odds ratio of association obtained from either Michailidou et al.^35^ for the SNPs associated with overall BC risk and from Milne et al.^48^ for the SNPs associated with ER-negative BC.

For all associated SNPs in case-only analyses, heterogeneity by country was assessed using likelihood ratio tests that compared models with and without an SNP by country interaction term. When the heterogeneity test was significant at *P* < 0.05, a leave-one-out analysis was performed, by excluding each country in turn to assess the influence of a data from a specific country on the overall association.

### Credible causal variants

For each novel region, we defined sets of credible causal variants (CCVs) to use in the prediction of the likely target genes. For this purpose, we defined a first set of CCVs including the top SNP of the region of interest and the SNPs with *p*-values of association within two orders of magnitude of the top SNP association. Then, we sequentially performed logistic regression analyses using all other SNPs in the region, adjusted for the top SNP. We defined a second set of CCVs which included the most significant SNP after adjusting for the top SNP and the SNPs with *p*-values within two orders of magnitude of the most significant SNP association. This was repeated (conditioning on the previously found most significant SNPs) to define additional sets of CCVs as long as at least one *p*-value remained <10^−6^.

### eQTL analysis

Data from BC tumours and adjacent normal breast tissue were accessed from The Cancer Genome Atlas^74^ (TCGA). Germline SNP genotypes (Affymetrix 6.0 arrays) from individuals of European ancestry were processed and imputed to the 1000 Genomes reference panel (October 2014)^35^. Tumour tissue copy number was estimated from the Affymetrix 6.0 and called using the GISTIC2 algorithm^75^. Complete genotype, RNA-seq and copy number data were available for 679 genetically European patients (78 with adjacent normal tissue). Further, RNA-seq for normal breast tissue and imputed germline genotype data were available from 80 females from the GTEx Consortium^76^. Genes with a median expression level of 0 RPKM across samples were removed, and RPKM values of each gene were log2 transformed. Expression values of samples were quantile normalized. Genetic variants were evaluated for association with the expression of genes located within ±2 Mb of the lead variant at each risk region using linear regression models, adjusting for *ESR1* expression. Tumour tissue was also adjusted for copy number variation^77^. eQTL analyses were performed using the MatrixEQTL program in R^78^.

### INQUISIT analyses

Candidate target genes were evaluated by assessing each CCV’s potential impact on regulatory or coding features using a computational pipeline, INtegrated expression QUantitative trait and In SIlico prediction of GWAS Targets (INQUISIT)^35,54^. Briefly, genes were considered as potential targets of candidate causal variants through effects on: (1) distal gene regulation, (2) proximal regulation, or (3) a gene’s coding sequence. We intersected CCV positions with multiple sources of genomic information chromatin interaction analysis by paired-end tag sequencing (ChIA-PET^79^) in MCF7 cells and genome-wide chromosome conformation capture (Hi-C) in HMECs. We used breast cell line computational enhancer–promoter correlations (PreSTIGE^80^, IM-PET^81^, FANTOM5^82^) breast cell super-enhancer^83^, breast tissue-specific expression variants (eQTL) from multiple independent studies (TCGA (normal breast and breast tumour) and GTEx breast—see eQTL methods), transcription factor and histone modification chromatin immunoprecipitation followed by sequencing (ChIP-seq) from the ENCODE and Roadmap Epigenomics Projects together with the genomic features found to be significantly enriched for all known breast cancer CCVs^54^, gene expression RNA-seq from several breast cancer lines and normal samples (ENCODE) and topologically associated domain (TAD) boundaries from T47D cells (ENCODE^84^). To assess the impact of intragenic variants, we evaluated their potential to alter primary protein coding sequence and splicing using Ensembl Variant Effect Predictor^85^ using MaxEntScan and dbscSNV modules for splicing alterations based on ada and rf scores. Nonsense and missense changes were assessed with the REVEL ensemble algorithm, with CCVs displaying REVEL scores >0.5 deemed deleterious.

Each target gene prediction category (distal, promoter or coding) was scored according to different criteria. Genes predicted to be distally regulated targets of CCVs were awarded points based on physical links (for example ChIA-PET), computational prediction methods, or eQTL associations. All CCVs were considered as potentially involved in distal regulation. Intersection of a putative distal enhancer with genomic features found to be significantly enriched^54^ were further upweighted. Multiple independent interactions were awarded an additional point. CCVs in gene proximal regulatory regions were intersected with histone ChIP-Seq peaks characteristic of promoters and assigned to the overlapping transcription start sites (defined as −1.0 kb – +0.1 kb). Further points were awarded to such genes if there was evidence for eQTL association, while a lack of expression resulted in down-weighting as potential targets. Potential coding changes including missense, nonsense and predicted splicing alterations resulted in addition of one point to the encoded gene for each type of change, while lack of expression reduced the score. We added an additional point for predicted target genes that were also breast cancer drivers (278 genes^35,54^). For each category, scores potentially ranged from 0 to 8 (distal); 0 to 4 (promoter) or 0 to 3 (coding). We converted these scores into ‘confidence levels’: Level 1 (highest confidence) when distal score > 4, promoter score ≥3 or coding score >1; Level 2 when distal score ≤ 4 and ≥1, promoter score = 1 or = 2, coding score = 1; and Level 3 when distal score <1 and >0, promoter score <1 and >0, and coding <1 and >0. For genes with multiple scores (for example, predicted as targets from multiple independent risk signals or predicted to be impacted in several categories), we recorded the highest score.

### Reporting summary

Further information on research design is available in the Nature Research Reporting Summary linked to this article.

## Supplementary information

Supplementary Information

Peer Review File

Description of Additional Supplementary Files

Supplementary Data 1-9

Reporting Summary

## Data Availability

Among BCAC data used in this study, data from 2SISTER, BREOGAN, CGPS, CPSII, EPIC, MEC, NBHS, MCCS, NHS, NHS2, PBCS, PLCO, SEARCH, SISTER, SMC, WAABCS and WHI are available in the dbGaP database under accession code phs001265.v1.p1. Among CIMBA data used in this study, data from KCONFAB, KUMC, MAYO, MSKCC, MUV, NCI, NNPIO, NORTHSHORE, OSU CCG, PBCS, SMC, SWE-BRCA, UCHICAGO, UCSF, UPENN, UPITT, UTMDACC, VFCTG and WCP studies are available in the dbGaP database under accession code phs001321.v1.p1. The complete dataset is not publicly available due to restraints imposed by the ethical committees of individual studies. Requests for the complete data can be made to the corresponding author or the Data Access Coordinating Committees (DACCs) of BCAC (BCAC@medschl.cam.ac.uk) and CIMBA (ljm26@medschl.cam.ac.uk). BCAC DACC approval is required to access data from the following studies ABCFS, ABCS, ABCTB, BBCC, BBCS, BCEES, BCFR-NY, BCFR-PA, BCFR-UTAH, BCINIS, BSUCH, CBCS, CECILE, CGPS, CTS, DIETCOMPLYF, ESTHER, GC-HBOC, GENICA, GEPARSIXTO, GESBC, HABCS, HCSC, HEBCS, HUBCS, KARBAC, KBCP, LMBC, MARIE, MBCSG, MCBCS, MISS, MMHS, MSKCC, MTLGEBCS, NC-BCFR, OFBCR, ORIGO, PBCS, pKARMA, POSH, PREFACE, RBCS, SKKDKFZS, SUCCESSB, SUCCESSC, SZBCS, TNBCC, UCIBCS, UKBGS and UKOPS (see Supplementary Table 2—for a list of all studies). CIMBA DACC approval is required to access data from studies BCFR-ON, BRICOH, CONSIT TEAM, DKFZ, EMBRACE, FPGMX, G-FAST, GC-HBOC, GEMO, HEBCS, HEBON, IHCC, INHERIT, IOVHBOCS, MCGILL, NRG_ONCOLOGY, OUH and UKGRFOCR (see Supplementary Table 1—for a list of all CIMBA studies). Case-control summary results from CIMBA and BCAC consortia are publicly available and can be downloaded at http://cimba.ccge.medschl.cam.ac.uk/oncoarray-complete-summary-results/ and at http://bcac.ccge.medschl.cam.ac.uk/bcacdata/oncoarray/oncoarray-and-combined-summary-result/gwas-summary-associations-breast-cancer-risk-2020/). The top 10 000 SNPs from the current BCAC-CIMBA case-only study can be found at http://cimba.ccge.medschl.cam.ac.uk/projects/BCAC-CIMBA_Case-only_analysis. The remaining data are available within the Article, Supplementary Information or available from the authors upon request. Source data are provided with this paper.

## Source data

Source Data
