# Author Correction: A case-only study to identify genetic modifiers of breast cancer risk for *BRCA1/BRCA2* mutation carriers

**DOI:** 10.1038/s41467-021-23162-4

**Published:** 2021-05-14

**Authors:** Juliette Coignard, Michael Lush, Jonathan Beesley, Tracy A. O’Mara, Joe Dennis, Jonathan P. Tyrer, Daniel R. Barnes, Lesley McGuffog, Goska Leslie, Manjeet K. Bolla, Muriel A. Adank, Simona Agata, Thomas Ahearn, Kristiina Aittomäki, Irene L. Andrulis, Hoda Anton-Culver, Volker Arndt, Norbert Arnold, Kristan J. Aronson, Banu K. Arun, Annelie Augustinsson, Jacopo Azzollini, Daniel Barrowdale, Caroline Baynes, Heko Becher, Marina Bermisheva, Leslie Bernstein, Katarzyna Białkowska, Carl Blomqvist, Stig E. Bojesen, Bernardo Bonanni, Ake Borg, Hiltrud Brauch, Hermann Brenner, Barbara Burwinkel, Saundra S. Buys, Trinidad Caldés, Maria A. Caligo, Daniele Campa, Brian D. Carter, Jose E. Castelao, Jenny Chang-Claude, Stephen J. Chanock, Wendy K. Chung, Kathleen B. M. Claes, Christine L. Clarke, GEMO Study Collaborators, Ophélie Bertrand, Sandrine Caputo, Anaïs Dupré, Marine Le Mentec, Muriel Belotti, Anne-Marie Birot, Bruno Buecher, Emmanuelle Fourme, Marion Gauthier-Villars, Lisa Golmard, Claude Houdayer, Virginie Moncoutier, Antoine de Pauw, Claire Saule, Olga Sinilnikova, Sylvie Mazoyer, Francesca Damiola, Laure Barjhoux, Carole Verny-Pierre, Mélanie Léone, Nadia Boutry-Kryza, Alain Calender, Sophie Giraud, Olivier Caron, Marine Guillaud-Bataille, Brigitte Bressac-de-Paillerets, Yves- Jean Bignon, Nancy Uhrhammer, Christine Lasset, Valérie Bonadona, Pascaline Berthet, Dominique Vaur, Laurent Castera, Tetsuro Noguchi, Cornel Popovici, Hagay Sobol, Violaine Bourdon, Tetsuro Noguchi, Audrey Remenieras, Catherine Noguès, Isabelle Coupier, Pascal Pujol, Aurélie Dumont, Françoise Révillion, Claude Adenis, Danièle Muller, Emmanuelle Barouk-Simonet, Françoise Bonnet, Virginie Bubien, Nicolas Sevenet, Michel Longy, Christine Toulas, Rosine Guimbaud, Laurence Gladieff, Viviane Feillel, Dominique Leroux, Hélène Dreyfus, Christine Rebischung, Magalie Peysselon, Fanny Coron, Laurence Faivre, Amandine Baurand, Caroline Jacquot, Geoffrey Bertolone, Sarab Lizard, Fabienne Prieur, Marine Lebrun, Caroline Kientz, Sandra Fert Ferrer, Véronique Mari, Laurence Vénat-Bouvet, Capucine Delnatte, Stéphane Bézieau, Isabelle Mortemousque, Florence Coulet, Chrystelle Colas, Florent Soubrier, Mathilde Warcoin, Johanna Sokolowska, Myriam Bronner, Marie-Agnès Collonge-Rame, Alexandre Damette, Paul Gesta, Hakima Lallaoui, Jean Chiesa, Denise Molina-Gomes, Olivier Ingster, EMBRACE Collaborators, Helen Gregory, Zosia Miedzybrodzka, Patrick J. Morrison, Kai-ren Ong, Alan Donaldson, Mark T. Rogers, M. John Kennedy, Mary E. Porteous, Carole Brewer, Rosemarie Davidson, Louise Izatt, Angela Brady, Julian Barwell, Julian Adlard, Claire Foo, Fiona Lalloo, Lucy E. Side, Jacqueline Eason, Alex Henderson, Lisa Walker, Rosalind A. Eeles, Jackie Cook, Katie Snape, Diana Eccles, Alex Murray, Emma McCann, J. Margriet Collée, Don M. Conroy, Kamila Czene, Mary B. Daly, Peter Devilee, Orland Diez, Yuan Chun Ding, Susan M. Domchek, Thilo Dörk, Isabel dos-Santos-Silva, Alison M. Dunning, Miriam Dwek, Diana M. Eccles, A. Heather Eliassen, Christoph Engel, Mikael Eriksson, D. Gareth Evans, Peter A. Fasching, Henrik Flyger, Florentia Fostira, Eitan Friedman, Lin Fritschi, Debra Frost, Manuela Gago-Dominguez, Susan M. Gapstur, Judy Garber, Vanesa Garcia-Barberan, Montserrat García-Closas, José A. García-Sáenz, Mia M. Gaudet, Simon A. Gayther, Andrea Gehrig, Vassilios Georgoulias, Graham G. Giles, Andrew K. Godwin, Mark S. Goldberg, David E. Goldgar, Anna González-Neira, Mark H. Greene, Pascal Guénel, Lothar Haeberle, Eric Hahnen, Christopher A. Haiman, Niclas Håkansson, Per Hall, Ute Hamann, Patricia A. Harrington, Steven N. Hart, Wei He, Frans B. L. Hogervorst, Antoinette Hollestelle, John L. Hopper, Darling J. Horcasitas, Peter J. Hulick, David J. Hunter, Evgeny N. Imyanitov, KConFab Investigators, Stephen Fox, Ian Campbell, Amanda Spurdle, Penny Webb, Anna de Fazio, Margaret Tassell, Judy Kirk, Geoff Lindeman, Melanie Price, Melissa Southey, Roger Milne, Sid Deb, David Bowtell, HEBON Investigators, Annemieke H. van der Hout, Ans M. W. van den Ouweland, Arjen R. Mensenkamp, Carolien H. M. van Deurzen, Carolien M. Kets, Caroline Seynaeve, Christi J. van Asperen, Cora M. Aalfs, Encarna B. Gómez Garcia, Flora E. van Leeuwen, G. H. de Bock, Hanne E. J. Meijers-Heijboer, Inge M. Obdeijn, J. Margriet Collée, J. J. P. Gille, Jan C. Oosterwijk, Juul T. Wijnen, Lizet E. van der Kolk, Maartje J. Hooning, Margreet G. E. M. Ausems, Marian J. E. Mourits, Marinus J. Blok, Matti A. Rookus, Muriel A. Adank, Rob B. van der Luijt, T. C. T. E. F. van Cronenburg, Carmen C. van der Pol, Nicola S. Russell, Sabine Siesling, Lucy Overbeek, R. Wijnands, Judith L. de Lange, ABCTB Investigators, Christine Clarke, Dinny Graham, Mythily Sachchithananthan, Deborah Marsh, Rodney Scott, Robert Baxter, Desmond Yip, Jane Carpenter, Alison Davis, Nirmala Pathmanathan, Peter Simpson, Agnes Jager, Anna Jakubowska, Paul A. James, Uffe Birk Jensen, Esther M. John, Michael E. Jones, Rudolf Kaaks, Pooja Middha Kapoor, Beth Y. Karlan, Renske Keeman, Elza Khusnutdinova, Johanna I. Kiiski, Yon-Dschun Ko, Veli-Matti Kosma, Peter Kraft, Allison W. Kurian, Yael Laitman, Diether Lambrechts, Loic Le Marchand, Jenny Lester, Fabienne Lesueur, Tricia Lindstrom, Adria Lopez-Fernández, Jennifer T. Loud, Craig Luccarini, Arto Mannermaa, Siranoush Manoukian, Sara Margolin, John W. M. Martens, Noura Mebirouk, Alfons Meindl, Austin Miller, Roger L. Milne, Marco Montagna, Katherine L. Nathanson, Susan L. Neuhausen, Heli Nevanlinna, Finn C. Nielsen, Katie M. O’Brien, Olufunmilayo I. Olopade, Janet E. Olson, Håkan Olsson, Ana Osorio, Laura Ottini, Tjoung-Won Park-Simon, Michael T. Parsons, Inge Sokilde Pedersen, Beth Peshkin, Paolo Peterlongo, Julian Peto, Paul D. P. Pharoah, Kelly-Anne Phillips, Eric C. Polley, Bruce Poppe, Nadege Presneau, Miquel Angel Pujana, Kevin Punie, Paolo Radice, Johanna Rantala, Muhammad U. Rashid, Gad Rennert, Hedy S. Rennert, Mark Robson, Atocha Romero, Maria Rossing, Emmanouil Saloustros, Dale P. Sandler, Regina Santella, Maren T. Scheuner, Marjanka K. Schmidt, Gunnar Schmidt, Christopher Scott, Priyanka Sharma, Penny Soucy, Melissa C. Southey, John J. Spinelli, Zoe Steinsnyder, Jennifer Stone, Dominique Stoppa-Lyonnet, Anthony Swerdlow, Rulla M. Tamimi, William J. Tapper, Jack A. Taylor, Mary Beth Terry, Alex Teulé, Darcy L. Thull, Marc Tischkowitz, Amanda E. Toland, Diana Torres, Alison H. Trainer, Thérèse Truong, Nadine Tung, Celine M. Vachon, Ana Vega, Joseph Vijai, Qin Wang, Barbara Wappenschmidt, Clarice R. Weinberg, Jeffrey N. Weitzel, Camilla Wendt, Alicja Wolk, Siddhartha Yadav, Xiaohong R. Yang, Drakoulis Yannoukakos, Wei Zheng, Argyrios Ziogas, Kristin K. Zorn, Sue K. Park, Mads Thomassen, Kenneth Offit, Rita K. Schmutzler, Fergus J. Couch, Jacques Simard, Georgia Chenevix-Trench, Douglas F. Easton, Nadine Andrieu, Antonis C. Antoniou

**Affiliations:** 1 Genetic Epidemiology of Cancer team, Inserm, U900, Paris, France; 2 Institut Curie Paris, Paris, France; 3 Mines ParisTech Fontainebleau, Paris, France; 4 Centre for Cancer Genetic Epidemiology, Department of Public Health and Primary Care, University of Cambridge, Cambridge, UK; 5 PSL University Paris, Paris, France; 6 Paris Sud University, Orsay, France; 7 Department of Genetics and Computational Biology QIMR Berghofer Medical Research Institute, Brisbane, QLD Australia; 8 Centre for Cancer Genetic Epidemiology, Department of Oncology University of Cambridge, Cambridge, UK; 9 Family Cancer Clinic, The Netherlands Cancer Institute, Antoni van Leeuwenhoek hospital, Amsterdam, The Netherlands; 10 Immunology and Molecular Oncology, Unit Veneto Institute of Oncology IOV – IRCCS, Padua, Italy; 11 Division of Cancer Epidemiology and Genetics National Cancer Institute, National Institutes of Health, Department of Health and Human Services, Bethesda, MD USA; 12 Department of Clinical Genetics, Helsinki University Hospital University of Helsinki, Helsinki, Finland; 13 Fred A. Litwin Center for Cancer Genetics Lunenfeld-Tanenbaum Research Institute of Mount Sinai Hospital, Toronto, ON Canada; 14 Department of Molecular Genetics University of Toronto, Toronto, ON Canada; 15 Department of Epidemiology, Genetic Epidemiology Research Institute University of California Irvine, Irvine, CA USA; 16 Division of Clinical Epidemiology and Aging Research German Cancer Research Center (DKFZ), Heidelberg, Germany; 17 Department of Gynaecology and Obstetrics University Hospital of Schleswig-Holstein, Campus Kiel, Christian-Albrechts University Kiel, Kiel, Germany; 18 Institute of Clinical Molecular Biology University Hospital of Schleswig-Holstein, Campus Kiel, Christian-Albrechts University Kiel, Kiel, Germany; 19 Department of Public Health Sciences, and Cancer Research Institute Queen’s University, Kingston, ON Canada; 20 Department of Breast Medical Oncology University of Texas MD Anderson Cancer Center, Houston, TX USA; 21 Department of Cancer Epidemiology, Clinical Sciences Lund University, Lund, 22242 Sweden; 22 Unit of Medical Genetics, Department of Medical Oncology and Hematology Fondazione IRCCS Istituto Nazionale dei Tumori di Milano, Milan, Italy; 23 Institute for Medical Biometrics and Epidemiology University Medical Center Hamburg-Eppendorf, Hamburg, Germany; 24 Institute of Biochemistry and Genetics Ufa Federal Research Centre of the Russian Academy of Sciences, Ufa, Russia; 25 Department of Population Sciences Beckman Research Institute of City of Hope, Duarte, CA USA; 26 Department of Genetics and Pathology Pomeranian Medical University Szczecin, Szczecin, Poland; 27 Department of Oncology, Helsinki University Hospital University of Helsinki, Helsinki, Finland; 28 Department of Oncology Örebro University Hospital, Örebro, Sweden; 29 Copenhagen General Population Study, Herlev and Gentofte Hospital Copenhagen University Hospital, Herlev, Denmark; 30 Department of Clinical Biochemistry, Herlev and Gentofte Hospital Copenhagen University Hospital, Herlev, Denmark; 31 Faculty of Health and Medical Sciences University of Copenhagen, Copenhagen, Denmark; 32 Division of Cancer Prevention and Genetics IEO, European Institute of Oncology IRCCS, Milan, Italy; 33 Department of Oncology Lund University and Skåne University Hospital, Lund, Sweden; 34 Dr. Margarete Fischer-Bosch-Institute of Clinical Pharmacology, Stuttgart, Germany; 35 iFIT-Cluster of Excellence University of Tübingen, Tübingen, Germany; 36 Division of Preventive Oncology, German Cancer Research Center (DKFZ) and National Center for Tumor Diseases (NCT), Heidelberg, Germany; 37 German Cancer Consortium (DKTK), German Cancer Research Center (DKFZ), Heidelberg, Germany; 39 Molecular Epidemiology Group, C080 German Cancer Research Center (DKFZ), Heidelberg, Germany; 40 Molecular Biology of Breast Cancer, University Womens Clinic Heidelberg University of Heidelberg, Heidelberg, Germany; 41 Department of Medicine Huntsman Cancer Institute, Salt Lake City, UT USA; 42 Molecular Oncology Laboratory CIBERONC, Hospital Clinico San Carlos, IdISSC (Instituto de Investigación Sanitaria del Hospital Clínico San Carlos), Madrid, Spain; 43 SOD Genetica Molecolare University Hospital, Pisa, Italy; 44 Department of Biology University of Pisa, Pisa, Italy; 45 Division of Cancer Epidemiology German Cancer Research Center (DKFZ), Heidelberg, Germany; 46 Behavioral and Epidemiology Research Group American Cancer Society Atlanta, Atlanta, GA USA; 47 Oncology and Genetics Unit Instituto de Investigacion Sanitaria Galicia Sur (IISGS), Xerencia de Xestion Integrada de Vigo-SERGAS, Vigo, Spain; 48 Cancer Epidemiology Group, University Cancer Center Hamburg (UCCH) University Medical Center Hamburg-Eppendorf, Hamburg, Germany; 49 Departments of Pediatrics and Medicine, Columbia University, New York, NY USA; 50 Centre for Medical Genetics Ghent University, Gent, Belgium; 51 Westmead Institute for Medical Research University of Sydney, Sydney, NSW Australia; 52 Department of Clinical Genetics Erasmus University Medical Center, Rotterdam, The Netherlands; 53 Department of Medical Epidemiology and Biostatistics, Karolinska Institutet, Stockholm, Sweden; 54 Department of Clinical Genetics Fox Chase Cancer Center Philadelphia, Philadelphia, PA USA; 55 Department of Pathology Leiden University Medical Center, Leiden, The Netherlands; 56 Department of Human Genetics Leiden University Medical Center, Leiden, The Netherlands; 57 Oncogenetics Group Vall d’Hebron Institute of Oncology (VHIO), Barcelona, Spain; 58 Clinical and Molecular Genetics Area University Hospital Vall d’Hebron, Barcelona, Spain; 59 Basser Center for BRCA, Abramson Cancer Center University of Pennsylvania, Philadelphia, PA USA; 60 Gynaecology Research Unit, Hannover Medical School, Hannover, Germany; 61 Department of Non-Communicable Disease Epidemiology London School of Hygiene and Tropical Medicine, London, UK; 62 School of Life Sciences University of Westminster, London, UK; 63 Faculty of Medicine University of Southampton, Southampton, UK; 64 Channing Division of Network Medicine, Department of Medicine, Brigham and Women’s Hospital, Harvard Medical School, Boston, MA USA; 65 Department of Epidemiology Harvard TH Chan School of Public Health, Boston, MA USA; 66 Institute for Medical Informatics, Statistics and Epidemiology University of Leipzig, Leipzig, Germany; 67 Genomic Medicine, Division of Evolution and Genomic Sciences The University of Manchester, Manchester Academic Health Science Centre, Manchester Universities Foundation Trust, St Mary’s Hospital, Manchester, UK; 68 Genomic Medicine, North West Genomics hub Manchester Academic Health Science Centre, Manchester Universities Foundation Trust, St Mary’s Hospital, Manchester, UK; 69 David Geffen School of Medicine, Department of Medicine Division of Hematology and Oncology University of California at Los Angeles, Los Angeles, CA USA; 70 Department of Gynecology and Obstetrics, Comprehensive Cancer Center ER-EMN University Hospital Erlangen, Friedrich-Alexander-University, Erlangen-Nuremberg, Erlangen, Germany; 71 Department of Breast Surgery, Herlev and Gentofte Hospital Copenhagen University Hospital, Herlev, Denmark; 72 Molecular Diagnostics Laboratory, INRASTES National Centre for Scientific Research íDemokritosí, Athens, Greece; 73 The Susanne Levy Gertner Oncogenetics Unit Chaim Sheba Medical Center, Ramat Gan, Israel; 74 Sackler Faculty of Medicine Tel Aviv University, Ramat Aviv, Israel; 75 School of Public Health Curtin University, Perth, Western Australia Australia; 76 Genomic Medicine Group, Galician Foundation of Genomic Medicine Instituto de Investigación Sanitaria de Santiago de Compostela (IDIS), Complejo Hospitalario Universitario de Santiago, SERGAS, Santiago de Compostela, Spain; 77 Moores Cancer Center University of California, San Diego La Jolla, CA USA; 78 Division of Cancer Genetics and Prevention, Dana-Farber Cancer Institute, Boston, MA USA; 79 Medical Oncology Department, Hospital Clínico San Carlos Instituto de Investigación Sanitaria San Carlos (IdISSC), Centro Investigación Biomédica en Red de Cáncer (CIBERONC), Madrid, Spain; 80 Center for Bioinformatics and Functional Genomics and the Cedars Sinai Genomics Core Cedars-Sinai Medical Center, Los Angeles, CA USA; 81 Department of Human Genetics University Würzburg, Würzburg, Germany; 82 Department of Medical Oncology University Hospital of Heraklion, Heraklion, Greece; 83 Cancer Epidemiology Division Cancer Council Victoria, Melbourne, VIC Australia; 84 Centre for Epidemiology and Biostatistics, Melbourne School of Population and Global Health, The University of Melbourne, Melbourne, VIC Australia; 85 Precision Medicine, School of Clinical Sciences at Monash Health Monash University, Clayton, VIC Australia; 86 Department of Pathology and Laboratory Medicine, University of Kansas Medical Center, Kansas City, KS USA; 87 Department of Medicine, McGill University, Montréal, QC Canada; 88 Division of Clinical Epidemiology, Royal Victoria Hospital McGill University Montréal, Montréal, QC Canada; 89 Huntsman Cancer Institute and Department of Dermatology, University of Utah School of Medicine, Salt Lake City, UT USA; 90 Human Cancer Genetics Programme Spanish National Cancer Research Centre (CNIO), Madrid, Spain; 91 Clinical Genetics Branch, Division of Cancer Epidemiology and Genetics National Cancer Institute, Bethesda, MD USA; 92 Cancer & Environment Group, Center for Research in Epidemiology and Population Health (CESP) INSERM, University Paris-Sud, University Paris-Saclay, Villejuif, France; 93 Department of Gynaecology and Obstetrics, University Hospital Erlangen Friedrich-Alexander University Erlangen-Nuremberg, Comprehensive Cancer Center Erlangen-EMN, Erlangen, Germany; 94 Center for Hereditary Breast and Ovarian Cancer Faculty of Medicine and University Hospital Cologne, University of Cologne, Cologne, Germany; 95 Center for Integrated Oncology (CIO) Faculty of Medicine and University Hospital Cologne, University of Cologne, Cologne, Germany; 96 Department of Preventive Medicine, Keck School of Medicine University of Southern California, Los Angeles, CA USA; 97 Institute of Environmental Medicine Karolinska Institutet, Stockholm, Sweden; 98 Department of Oncology, Södersjukhuset, Stockholm, Sweden; 99 Molecular Genetics of Breast Cancer German Cancer Research Center (DKFZ), Heidelberg, Germany; 100 Department of Health Sciences Research, Mayo Clinic, Rochester, MN USA; 101 Department of Medical Oncology, Family Cancer Clinic Erasmus MC Cancer Institute, Rotterdam, The Netherlands; 102 New Mexico Oncology Hematology Consultants, University of New Mexico, Albuquerque, NM USA; 103 Center for Medical Genetics NorthShore University HealthSystem, Evanston, IL USA; 104 The University of Chicago Pritzker School of Medicine Chicago, Chicago, IL USA; 105 Program in Genetic Epidemiology and Statistical Genetics Harvard TH Chan School of Public Health Boston, Boston, MA USA; 106 Nuffield Department of Population Health University of Oxford, Oxford, UK; 107 NN Petrov Institute of Oncology, St. Petersburg, Russia; 108 Independent Laboratory of Molecular Biology and Genetic Diagnostics Pomeranian Medical University, Szczecin, Poland; 109 Sir Peter MacCallum Department of Oncology The University of Melbourne, Melbourne, VIC Australia; 110 Parkville Familial Cancer Centre Peter MacCallum Cancer Center, Melbourne, VIC Australia; 111 Department of Clinical Genetics Aarhus, University Hospital, Aarhus, Denmark; 112 Department of Medicine, Division of Oncology, Stanford University School of Medicine, Stanford, CA USA; 114 Department of Epidemiology and Population Health, Stanford University School of Medicine, Stanford, CA USA; 115 Division of Genetics and Epidemiology The Institute of Cancer Research, London, UK; 116 Faculty of Medicine University of Heidelberg, Heidelberg, Germany; 117 David Geffen School of Medicine, Department of Obstetrics and Gynecology University of California at Los Angeles, Los Angeles, CA USA; 118 Womenís Cancer Program at the Samuel Oschin Comprehensive Cancer Institute Cedars-Sinai Medical Center, Los Angeles, CA USA; 119 Division of Molecular Pathology The Netherlands Cancer Institute - Antoni van Leeuwenhoek Hospital, Amsterdam, The Netherlands; 120 Department of Genetics and Fundamental Medicine Bashkir State Medical University, Ufa, Russia; 121 Department of Obstetrics and Gynecology, Helsinki University Hospital University of Helsinki, Helsinki, Finland; 122 Department of Internal Medicine, Evangelische Kliniken Bonn gGmbH Johanniter Krankenhaus, Bonn, Germany; 123 Translational Cancer Research Area University of Eastern Finland, Kuopio, Finland; 124 Institute of Clinical Medicine, Pathology and Forensic Medicine University of Eastern Finland, Kuopio, Finland; 125 VIB Center for Cancer Biology, Leuven, Belgium; 126 Laboratory for Translational Genetics, Department of Human Genetics University of Leuven, Leuven, Belgium; 127 Epidemiology Program University of Hawaii Cancer Center, Honolulu, HI USA; 128 High Risk and Cancer Prevention Group Vall d’Hebron Institute of Oncology, Barcelona, Spain; 129 Department of Clinical Science and Education, Södersjukhuset Karolinska Institutet, Stockholm, Sweden; 130 Department of Gynecology and Obstetrics University of Munich, Campus Grosshadern, Munich, Germany; 131 NRG Oncology, Statistics and Data Management Center Roswell Park Cancer Institute, Buffalo, NY USA; 132 Center for Genomic Medicine Rigshospitalet, Copenhagen University Hospital, Copenhagen, Denmark; 133 Epidemiology Branch National Institute of Environmental Health Sciences, NIH Research Triangle Park, Durham, NC USA; 134 Center for Clinical Cancer Genetics The University of Chicago, Chicago, IL USA; 135 Centro de Investigación en Red de Enfermedades Raras (CIBERER), Madrid, Spain; 136 Department of Molecular Medicine University La Sapienza, Rome, Italy; 137 Molecular Diagnostics Aalborg University Hospital, Aalborg, Denmark; 138 Clinical Cancer Research Center Aalborg University Hospital, Aalborg, Denmark; 139 Department of Clinical Medicine Aalborg University, Aalborg, Denmark; 140 Lombardi Comprehensive Cancer Center, Georgetown University, Washington, DC USA; 141 Genome Diagnostics Program IFOM - the FIRC (Italian Foundation for Cancer Research) Institute of Molecular Oncology, Milan, Italy; 142 Translational Research Laboratory IDIBELL (Bellvitge Biomedical Research Institute), Catalan Institute of Oncology, CIBERONC, Barcelona, Spain; 143 Leuven Multidisciplinary Breast Center, Department of Oncology Leuven Cancer Institute, University Hospitals Leuven, Leuven, Belgium; 144 Unit of Molecular Bases of Genetic Risk and Genetic Testing, Department of Research Fondazione IRCCS Istituto Nazionale dei Tumori (INT), Milan, Italy; 145 Clinical Genetics Karolinska Institutet, Stockholm, Sweden; 146 Department of Basic Sciences Shaukat Khanum Memorial Cancer Hospital and Research Centre (SKMCH & RC), Lahore, Pakistan; 147 Clalit National Cancer Control Center Carmel Medical Center and Technion Faculty of Medicine, Haifa, Israel; 148 Clinical Genetics Service, Department of Medicine Memorial Sloan-Kettering Cancer Center, New York, NY USA; 149 Medical Oncology Department Hospital Universitario Puerta de Hierro, Madrid, Spain; 150 Department of Oncology University Hospital of Larissa, Larissa, Greece; 151 Department of Epidemiology, Mailman School of Public Health Columbia University, New York, NY USA; 152 Cancer Genetics and Prevention Program University of California San Francisco, San Francisco, CA USA; 153 Division of Psychosocial Research and Epidemiology The Netherlands Cancer Institute - Antoni van Leeuwenhoek hospital, Amsterdam, The Netherlands; 154 Institute of Human Genetics Hannover Medical School, Hannover, Germany; 155 Department of Internal Medicine, Division of Medical Oncology University of Kansas Medical Center, Westwood, KS USA; 156 Genomics Center, Centre Hospitalier Universitaire de Québec - Université Laval Research Center, Québec City, QC Canada; 157 Department of Clinical Pathology The University of Melbourne, Melbourne, VIC Australia; 158 Population Oncology BC Cancer, Vancouver, BC Canada; 159 School of Population and Public Health University of British Columbia, Vancouver, BC Canada; 160 Clinical Genetics Research Lab, Department of Cancer Biology and Genetics Memorial Sloan-Kettering Cancer Center, New York, NY USA; 161 The Curtin UWA Centre for Genetic Origins of Health and Disease Curtin University and University of Western Australia, Perth, Western Australia Australia; 162 Service de Génétique Institut Curie, Paris, France; 163 Department of Tumour Biology INSERM U830, Paris, France; 164 Université Paris Descartes, Paris, France; 165 Division of Breast Cancer Research Institute of Cancer Research, London, UK; 167 Epigenetic and Stem Cell Biology Laboratory National Institute of Environmental Health Sciences, NIH Research Triangle Park, Triangle Park, NC USA; 168 Hereditary Cancer Program ONCOBELL-IDIBELL-IDIBGI-IGTP, Catalan Institute of Oncology, CIBERONC, Barcelona, Spain; 169 Department of Medicine Magee-Womens Hospital, University of Pittsburgh School of Medicine, Pittsburgh, PA USA; 170 Program in Cancer Genetics, Departments of Human Genetics and Oncology McGill University, Montréal, QC Canada; 171 Department of Medical Genetics, National Institute for Health Research Cambridge Biomedical Research Center, University of Cambridge, Cambridge, UK; 172 Department of Cancer Biology and Genetics The Ohio State University, Columbus, OH USA; 173 Institute of Human Genetics Pontificia Universidad Javeriana, Bogota, Colombia; 174 Department of medicine University Of Melbourne, Melbourne, VIC Australia; 175 Department of Medical Oncology Beth Israel Deaconess Medical Center, Boston, MA USA; 176 Department of Health Science Research, Division of Epidemiology Mayo Clinic, Rochester, MN USA; 177 Fundación Pública Galega Medicina Xenómica-SERGAS, Instituto de Investigación Sanitaria Santiago de Compostela (IDIS); CIBERER, Santiago de Compostela, Spain; 178 Biostatistics and Computational Biology Branch National Institute of Environmental Health Sciences, NIH Research Triangle Park, Triangle Park, NC USA; 179 Clinical Cancer Genomics City of Hope, Duarte, CA USA; 180 Department of Surgical Sciences Uppsala University, Uppsala, Sweden; 181 Department of Oncology Mayo Clinic, Rochester, MN USA; 182 Division of Epidemiology, Department of Medicine, Vanderbilt Epidemiology Center, Vanderbilt-Ingram Cancer Center Vanderbilt University School of Medicine, Nashville, TN USA; 183 Magee-Womens Hospital, University of Pittsburgh School of Medicine, Pittsburgh, PA USA; 184 Department of Preventive Medicine Seoul National University College of Medicine, Seoul, Korea; 185 Department of Biomedical Sciences Seoul National University Graduate School, Seoul, Korea; 186 Cancer Research Institute Seoul National University, Seoul, Korea; 187 Department of Clinical Genetics Odense University Hospital, Odence C, Denmark; 188 Department of Laboratory Medicine and Pathology Mayo Clinic, Rochester, MN USA; 189 Unité Mixte de Génétique Constitutionnelle des Cancers Fréquents, Hospices Civils de Lyon - Centre Léon Bérard, Lyon, France; 190 Institut Gustave Roussy, Villejuif, France; 191 Centre Jean Perrin, Clermont–Ferrand, France; 192 Centre Léon Bérard, Lyon, France; 193 Centre François Baclesse, Caen, France; 194 Institut Paoli Calmettes, Marseille, France; 195 CHU Arnaud-de-Villeneuve, Montpellier, France; 196 Centre Oscar Lambret, Lille, France; 197 Centre Paul Strauss, Strasbourg, France; 198 Institut Bergonié, Bordeaux, France; 199 Institut Claudius Regaud, Toulouse, France; 200 CHU, Grenoble, France; 201 CHU, Dijon, France; 202 CHU, St-Etienne, France; 203 Hôtel Dieu Centre Hospitalier, Chambéry, France; 204 Centre Antoine Lacassagne, Nice, France; 205 CHU, Limoges, France; 206 CHU, Nantes, France; 207 CHU Bretonneau, Tours, France; 208 Centre Hospitalier de, Bourges, France; 209 Groupe Hospitalier Pitié- Salpétrière, Paris, France; 210 CHU Vandoeuvre-, les-Nancy, France; 211 CHU, Besançon, France; 212 CHU Poitiers, Centre Hospitalier d’Angoulême, Poitiers, France; 213 Centre Hospitalier de Niort, Niort, France; 214 Centre Hospitalier de La Rochelle, La Rochelle, France; 215 CHU Nîmes Carémeau, Nîmes, France; 216 CHU, Poissy, France; 217 CHU, Angers, France; 218 North of Scotland Regional Genetics Service, NHS Grampian & University of Aberdeen, Foresterhill, Aberdeen, UK; 219 Northern Ireland Regional Genetics Centre, Belfast Health and Social Care Trust, and Department of Medical Genetics, Queens University Belfast, Belfast, UK; 220 West Midlands Regional Genetics Service, Birmingham Women’s Hospital Healthcare NHS Trust, Edgbaston, Birmingham, UK; 221 Clinical Genetics Department, St Michael’s Hospital, Bristol, UK; 222 All Wales Medical Genetics Services, University Hospital of Wales, Cardiff, UK; 223 Academic Unit of Clinical and Molecular Oncology, Trinity College Dublin and St James’s Hospital, Dublin, Eire; 224 South East of Scotland Regional Genetics Service, Western General Hospital, Edinburgh, UK; 225 Department of Clinical Genetics, Royal Devon & Exeter Hospital, Exeter, UK; 226 Clinical Genetics, Southern General Hospital, Glasgow, UK; 227 Clinical Genetics, Guy’s and St. Thomas’ NHS Foundation Trust, London, UK; 228 North West Thames Regional Genetics Service, Kennedy-Galton Centre, Harrow, UK; 229 Leicestershire Clinical Genetics Service, University Hospitals of Leicester NHS Trust, Leicester, UK; 230 Yorkshire Regional Genetics Service, Leeds, UK; 231 Department of Clinical Genetics, Alder Hey Hospital, Eaton Road, Liverpool, UK; 232 Genetic Medicine, Manchester Academic Health Sciences Centre, Central Manchester University Hospitals NHS Foundation Trust, Manchester, UK; 233 North East Thames Regional Genetics Service, Great Ormond Street Hospital for Children NHS Trust, London, UK; 234 Nottingham Clinical Genetics Service, Nottingham University Hospitals NHS Trust, Nottingham, UK; 235 Institute of Genetic Medicine, Centre for Life, Newcastle Upon Tyne Hospitals NHS Trust, Newcastle upon Tyne, UK; 236 Oxford Regional Genetics Service, Churchill Hospital, Oxford, UK; 237 Oncogenetics Team, The Institute of Cancer Research and Royal Marsden NHS Foundation Trust, London, UK; 238 Sheffield Clinical Genetics Service, Sheffield Children’s Hospital, Sheffield, UK; 239 South West Thames Regional Genetics Service, St.Georges Hospital, Cranmer Terrace, Tooting, London, UK; 240 All Wales Medical Genetics Services, Singleton Hospital, Swansea, UK; 241 All Wales Medical Genetics Service, Glan Clwyd Hospital, Rhyl, UK; 242 Peter MacCallum Cancer Centre, Melbourne, Australia; 243 Westmead Millenium Institute, Sydney, Australia; 244 BCNA delegate, Community Representative, Melbourne, Australia; 245 Westmead Hospital, Sydney, Australia; 246 Walter and Eliza Hall Institute, Melbourne, Australia; 247 University of Sydney, Sydney, Australia; 248 Melbourne Health, Melbourne, Australia; 249 Garvan Institute of Medical Research, Sydney, Australia; 250 Department of Genetics, University Medical Center Groningen, Groningen, The Netherlands; 251 Department of Clinical Genetics, Family Cancer Clinic, Erasmus University Medical Center, Rotterdam, The Netherlands; 252 Department of Human Genetics, Radboud University Medical Center, Nijmegen, The Netherlands; 253 Department of Pathology, Family Cancer Clinic, Erasmus University Medical Center, Rotterdam, The Netherlands; 254 Department of Clinical Genetics, Leiden University Medical Center, Leiden, The Netherlands; 255 Department of Clinical Genetics, Academic Medical Center, Amsterdam, The Netherlands; 256 Department of Clinical Genetics, MUMC, Maastricht, The Netherlands; 257 Department of Epidemiology, Netherlands Cancer Institute, Amsterdam, The Netherlands; 258 Department of Oncological Epidemiology, University Medical Center, Groningen University, Groningen, The Netherlands; 259 Department of Clinical Genetics, VU University Medical Centre, Amsterdam, The Netherlands; 260 Department of Radiology, Family Cancer Clinic, Erasmus University Medical Center, Rotterdam, The Netherlands; 261 Department of Medical Genetics, University Medical Center Utrecht, Utrecht, The Netherlands; 262 Department of Gynaecological Oncology, University Medical Center, Groningen University, Groningen, The Netherlands; 263 Department of Clinical Genetics, Maastricht University Medical Center, Maastricht, The Netherlands; 264 Department of Oncological and Endocrine Surgery, University Medical Center Utrecht, Utrecht, The Netherlands; 265 Department of Radiotherapy, Netherlands Cancer Institute, Amsterdam, The Netherlands; 266 The Netherlands Comprehensive Cancer Organization (IKNL), Utrecht, The Netherlands; 267 Foundation PALGA (The Nationwide Network and Registry of Histo- and Cytopathology in the Netherlands), Houten, The Netherlands; 268 University of Technology Sydney, Translational Oncology Group, School of Life Sciences, Faculty of Science, Ultimo, NSW Australia; 269 School of Biomedical Sciences, University of Newcastle, Newcastle; Hunter Medical Research Institute and NSW Health Pathology North, Newcastle, Australia; 270 Kolling Institute of Medical Research, University of Sydney, St Leonards, NSW Australia; 271 Department of Medical Oncology, The Canberra Hospital, Canberra, ACT Australia; 272 Scientific Platforms, The Westmead Institute for Medical Research, The University of Sydney, Sydney, NSW Australia; 273 The Canberra Hospital, Garran, ACT; The Australian National University, Canberra, ACT Australia; 274 Westmead Breast Cancer Institute, Western Sydney Local Health District, Westmead, New South Wales, Australia; 275 University of Sydney, Western Clinical School, Westmead, New South Wales, Australia; 276 UQ Centre for Clinical Research, Faculty of Medicine, The University of Queensland, Herston, QLD Australia

**Keywords:** Breast cancer, Cancer epidemiology, Cancer genetics, Risk factors, Breast cancer, Cancer epidemiology, Cancer genetics, Risk factors

## Abstract

A Correction to this paper has been published: https://doi.org/10.1038/s41467-021-23162-4

Correction to: *Nature Communications* 10.1038/s41467-020-20496-3, published online 17 February 2021.

The original version of this Article contained an error in the spelling of the author Heiko Becher, which was incorrectly given as Heko Becher. This has now been corrected in both the PDF and HTML versions of the Article.

