# Supplementary material for: A case-only study to identify genetic modifiers of breast cancer risk for BRCA1/BRCA2 mutation carriers
Source: Nat Commun. 2021 Feb 17;12:1078. doi: 10.1038/s41467-020-20496-3 (PMC7890067; doi:10.1038/s41467-020-20496-3)
Supplement: Supplementary file 1 — Supplementary Information [file 41467_2020_20496_MOESM1_ESM.pdf]

Supplementary Information PDF

« A case-only study to identify genetic modifiers of breast cancer risk for *BRCA1/BRCA2* mutation carriers » , Coignard *et al.*

Supplementary information

Supplementary tables

Supplementary table 1- Participating studies from CIMBA

Supplementary table 2- Participating studies from BCAC

Supplementary Table 3- Number of case subjects per country and study

Supplementary Table 4- Number of control subjects per country and study

Supplementary figures

Supplementary figure 1- Heterogeneity in the SNP associations by country for the SNPs found to be associated with *BRCA1* mutation status

Supplementary figure 2- Sensitivity analysis for the SNPs showing associations with *BRCA1* mutation status in the case only analysis

Supplementary figure 3- Heterogeneity in the SNP associations by country for the SNPs found to be associated with *BRCA2* mutation status

Supplementary figure 4- Sensitivity analysis for the SNPs showing associations with *BRCA2* mutation status in the case only analysis

Supplementary figure 5- Impact of the control-only analysis on the case-only analysis results

Supplementary table 1- Participating studies from CIMBA

Study Acronym	Study Name	Country
BCFR-AU	Australian site of the Breast Cancer Family Registry	Australia
KCONFAB	Kathleen Cuningham Consortium for Research into Familial Breast Cancer	Australia
VFCTG	Victorian Familial Cancer Trials Group	Australia
G-FAST	Ghent University Hospital	Belgium
BCFR-ON/OCGN	Ontario site of the Breast Cancer Family Registry/Ontario Cancer Genetics Network	Canada
INHERIT	INterdisciplinary HEalth Research Internal Team BReast CAncer susceptibility	Canada (Quebec)
MCGILL	McGill University	Canada (Quebec)
CBCS	Copenhagen Breast Cancer Study	Denmark
OUH	Odense University Hospital	Denmark
HEBCS	Helsinki Breast Cancer Study	Finland
GEMO	Genetic Modifiers of cancer risk in BRCA1/2 mutation carriers	France/USA
GC-HBOC	German Familial Breast Group	Germany
DKFZ	German Cancer Research Center	Germany/Pakistan/Colombia
DEMOKRITOS	National Centre for Scientific Research Demokritos	Greece
SMC	Sheba Medical Centre	Israel
CONSIT TEAM	CONsorzio Studi ITaliani sui Tumori Ereditari Alla Mammella	Italy
IOVHBOCS	Istituto Oncologico Veneto	Italy
PBCS	Università di Pisa	Italy
HEBON	Hereditary Breast and Ovarian cancer study the Netherlands	Netherlands
IHCC	International Hereditary Cancer Centre	Poland
NNPIO	N.N. Petrov Institute of Oncology	Russia
CNIO	Spanish National Cancer Centre	Spain
FPGMX	Fundación Pública Galega de Medicina Xenómica	Spain
HCSC	Hospital Clinico San Carlos	Spain
HVH	University Hospital Vall d'Hebron	Spain
ICO	Institut Català d'Oncologia	Spain
SWE-BRCA	Swedish Breast Cancer Study	Sweden
EMBRACE	Epidemiological Study of Familial Breast Cancer	UK
UKGRFOCR	UK and Gilda Radner Familial Ovarian Cancer Registries	UK/USA

BCFR-NC	Northern California site of the Breast Cancer Family Registry	USA
BCFR-NY	New York site of the Breast Cancer Family Registry	USA
BCFR-PA	Philadelphia site of the Breast Cancer Family Registry	USA
BCFR-UT	Utah site of the Breast Cancer Family Registry	USA
BIDMC	Beth Israel Deaconess Medical Center	USA
BRICOH	Beckman Research Institute of the City of Hope	USA
COH	City of Hope Cancer Center	USA
DFCI	Dana Farber Cancer Institute	USA
FCCC	Fox Chase Cancer Center	USA
GEORGETOWN	Georgetown University	USA
KUMC	University of Kansas Medical Center	USA
MAYO	Mayo Clinic	USA
MSKCC	Memorial Sloane Kettering Cancer Center	USA
NCI	National Cancer Institute	USA
NORTHSHORE	NorthShore University HealthSystem	USA
OSU CCG	The Ohio State University Comprehensive Cancer Center	USA
UCHICAGO	University of Chicago	USA
UCSF	University of California San Francisco	USA
UPENN	University of Pennsylvania	USA
UPITT	Cancer Family Registry University of Pittsburg	USA
UTMDACC	University of Texas MD Anderson Cancer Center	USA
WCP	Women’s Cancer Program at Cedars-Sinai Medical Center	USA
NRG_ONCOLOGY	NRG Oncology	USA/Australia

Supplementary table 2- Participating studies from BCAC

			Included in the analysis	
Study Acronym	***Study Name***	***Country***	***Case-only***	***Control-only***
ABCFS	*Australian Breast Cancer Family Study*	*Australia*	*yes*	*yes*
ABCTB	*Australian Breast Cancer Tissue Bank*	*Australia*	*yes*	*yes*
BCEES	*Breast Cancer Employment and Environment Study*	*Australia*	*yes*	*yes*
MCCS	*Melbourne Collaborative Cohort Study*	*Australia*	*yes*	*yes*
LMBC	*Leuven Multidisciplinary Breast Centre*	*Belgium*	*yes*	*yes*
CBCS	*Canadian Breast Cancer Study*	*Canada*	*yes*	*yes*
MTLGEBCS	*Montreal Gene-Environment Breast Cancer Study*	*Canada*	*yes*	*yes*
OFBCR	*Ontario Familial Breast Cancer Registry*	*Canada*	*yes*	*yes*
CGPS	*Copenhagen General Population Study*	*Denmark*	*yes*	*yes*
HEBCS	*Helsinki Breast Cancer Study*	*Finland*	*yes*	*yes*
KBCP	*Kuopio Breast Cancer Project*	*Finland*	*yes*	*yes*
CECILE	*CECILE Breast Cancer Study*	*France*	*yes*	*yes*
BBCC	*Bavarian Breast Cancer Cases and Controls*	*Germany*	*yes*	*yes*
BSUCH	*Breast Cancer Study of the University of Heidelberg*	*Germany*	*yes*	*yes*
ESTHER	*ESTHER Breast Cancer Study*	*Germany*	*yes*	*yes*
GC-HBOC	*German Consortium for Hereditary Breast & Ovarian Cancer*	*Germany*	*yes*	*yes*
GENICA	*Gene Environment Interaction and Breast Cancer in Germany*	*Germany*	*yes*	*yes*
GEPARSIXTO	*A randomized phase II trial investigating the addition of carboplatin to neoadjuvant therapy for triple-negative and HER2-positive early breast cancer*	*Germany*	*no*	*no*
GESBC	*Genetic Epidemiology Study of Breast Cancer by Age 50*	*Germany*	*yes*	*yes*
HABCS	*Hannover Breast Cancer Study*	*Germany*	*yes*	*yes*
MARIE	*Mammary Carcinoma Risk Factor Investigation*	*Germany*	*yes*	*yes*
PREFACE	*Evaluation of Predictive Factors regarding the Effectivity of Aromatase Inhibitor Therapy*	*Germany*	*no*	*no*
SKKDKFZS	*Städtisches Klinikum Karlsruhe Deutsches Krebsforschungszentrum Study*	*Germany*	*yes*	*no*
SUCCESSB	*Simultaneous Study of Gemcitabine-Docetaxel Combination adjuvant treatment*	*Germany*	*no*	*no*
SUCCESSC	*Simultaneous Study of Docetaxel Based Anthracycline Free Adjuvant Treatment Evaluation*	*Germany*	*no*	*no*
CCGP	*Crete Cancer Genetics Program*	*Greece*	*yes*	*yes*
BCINIS	*Breast Cancer In Northern Israel Study*	*Israel*	*yes*	*yes*

MBCSG	*Milan Breast Cancer Study Group*	*Italy*	*yes*	*yes*
ABCS	*Amsterdam Breast Cancer Study*	*Netherlands*	*yes*	*yes*
ORIGO	*Leiden University Medical Centre Breast Cancer Study*	*Netherlands*	*yes*	*yes*
RBCS	*Rotterdam Breast Cancer Study*	*Netherlands*	*yes*	*yes*
PBCS	*NCI Polish Breast Cancer Study*	*Poland*	*yes*	*yes*
SZBCS	*IHCC-Szczecin Breast Cancer Study*	*Poland*	*yes*	*yes*
HUBCS	*Hannover-Ufa Breast Cancer Study*	*Russia*	*yes*	*yes*
BREOGAN	*Breast Oncology Galicia Network*	*Spain*	*yes*	*yes*
HCSC	*Hospital Clinico San Carlos*	*Spain*	*yes*	*no*
KARBAC	*Karolinska Breast Cancer Study*	*Sweden*	*yes*	*no*
MISS	*Melanoma Inquiry of Southern Sweden*	*Sweden*	*yes*	*yes*
pKARMA	*Karolinska Mammography Project for Risk Prediction of Breast Cancer – Case-Control Study*	*Sweden*	*yes*	*yes*
SMC	*Swedish Mammography Cohort*	*Sweden*	*yes*	*yes*
BBCS	*British Breast Cancer Study*	*UK*	*yes*	*yes*
DIETCOMPLYF	*DietCompLyf Breast Cancer Survival Study*	*UK*	*yes*	*no*
POSH	*Prospective Study of Outcomes in Sporadic Versus Hereditary Breast Cancer*	*UK*	*yes*	*no*
SEARCH	*Study of Epidemiology and Risk factors in Cancer Heredity*	*UK*	*yes*	*yes*
UKBGS	*UK Breakthrough Generations Study*	*UK*	*yes*	*yes*
UKOPS	*UK Ovarian Cancer Population Study*	*UK*	*no*	*yes*
2SISTER	*The Two Sister Study*	*USA*	*yes*	*no*
BCFR-NY	*New York Breast Cancer Family Registry*	*USA*	*yes*	*yes*
BCFR-PA	*Philadelphia Breast Cancer Family Registry*	*USA*	*yes*	*no*
BCFR-UTAH	*Utah Breast Cancer Family Registry*	*USA*	*yes*	*no*
CPSII	*Cancer Prevention Study-II Nutrition Cohort*	*USA*	*yes*	*yes*
CTS	*California Teachers Study*	*USA*	*yes*	*yes*
MCBCS	*Mayo Clinic Breast Cancer Study*	*USA*	*yes*	*yes*
MEC	*Multiethnic Cohort*	*USA*	*yes*	*yes*
MMHS	*Mayo Mammography Health Study*	*USA*	*yes*	*yes*
MSKCC	*Memorial Sloan-Kettering Cancer Center Study*	*USA*	*yes*	*no*
NBHS	*Nashville Breast Health Study*	*USA*	*yes*	*yes*
NC-BCFR	*Northern California Breast Cancer Family Registry*	*USA*	*yes*	*yes*
NHS	*Nurses Health Study*	*USA*	*yes*	*yes*

NHS2	*Nurses Health Study 2*	*USA*	*yes*	*yes*
PLCO	*The Prostate, Lung, Colorectal and Ovarian (PLCO) Cancer Screening Trial*	*USA*	*yes*	*yes*
SISTER	*The Sister Study*	*USA*	*yes*	*yes*
UCIBCS	*UCI Breast Cancer Study*	*USA*	*yes*	*yes*
EPIC	*European Prospective Investigation Into Cancer and Nutrition (BPC3)*	*Various*	*yes*	*yes*
TNBCC	*Triple Negative Breast Cancer Consortium Study*	*Various*	*yes*	*no*

Supplementary Table 3- Number of case subjects per country and study

Country	BCAC Study	Number of cases		CIMBA study	Number of BRCA1 cases	Number of BRCA2 cases
Australia	MCCS	1051		NRG_ONCOLOGY	1	4
	ABCTB	951		KCONFAB	368	295
	ABCFS	1087		BCFR-AU	25	28
	BCEES	783		VFCTG	103	70
		**Total : 3872 (6,42%)**			**Total : 497 (6,85%)**	**Total : 397 (7,79%)**
Belgium	LMBC	789		G-FAST	121	76
		**Total : 789 (1,31%)**			**Total : 121 (1,67%)**	**Total : 76 (1,49%)**
Canada	CBCS	676		MCGILL	24	14
	OFBCR	1658		BCFR-ON	88	60
	MTLGEBCS	341		OCGN	71	64
				INHERIT	37	34
		**Total : 2675 (4,43%)**			**Total : 220 (3,03%)**	**Total : 172 (3,37%)**
Denmark	CGPS	1411		CBCS	76	64
				OUH	191	167
		**Total : 1411 (2,34%)**			**Total : 267 (3,68%)**	**Total : 231 (4,53%)**
Finland	HEBCS	281		HEBCS	53	67
	KBCP	556				
		**Total : 837 (1,39%)**			**Total : 53 (0,73%)**	**Total : 67 (1,31%)**
France	CECILE	306		GEMO	758	563
	EPIC	433				
		**Total : 739 (1,23%)**			**Total : 758 (10,45%)**	**Total : 563 (11,04%)**
Germany	ESTHER	296		GC-HBOC	1168	646
	SKKDKFZS	1091		DKFZ	36	14

	GESBC	351				
	GENICA	460				
	BBCC	441				
	MARIE	512				
	BSUCH	269				
	EPIC	661				
	GC-HBOC	3634				
	HABCS	929				
		**Total : 8644 (14,33%)**			**Total : 1204 (16,59%)**	**Total : 660 (12,95%)**
Greece	EPIC	182		DEMOKRITOS	132	23
	CCGP	670				
		**Total : 852 (1,41%)**			**Total : 132 (1,82%)**	**Total : 23 (0,45%)**
Israel	BCINIS	1330		SMC	66	33
		**Total : 1330 (2,2%)**			**Total : 66 (0,91%)**	**Total : 33 (0,65%)**
Italy	EPIC	822		CONSIT TEAM	271	187
	MBCSG	787		IOVHBOCS	109	113
				PBCS	49	6
		**Total : 1609 (2,67%)**			**Total : 429 (5,91%)**	**Total : 306 (6%)**
Netherlands	RBCS	473		HEBON	374	199
	EPIC	709				
	ORIGO	1055				
	ABCS	267				
		**Total : 2504 (4,15%)**			**Total : 374 (5,15%)**	**Total : 199 (3,9%)**
Poland	PBCS	1931		IHCC	77	0
	SZBCS	379				
		**Total : 2310 (3,83%)**			**Total : 77 (1,06%)**	**Total : 0 (0%)**

Russia	HUBCS	211		BIDMC	1	0
				NNPIO	44	2
		**Total : 211 (0,35%)**			**Total : 45 (0,62%)**	**Total : 2 (0,04%)**
Spain	BREOGAN	1376		HCSC	56	76
	EPIC	337		ICO	130	185
	HCSC	426		HVH	62	63
				FPGMX	67	44
				CNIO	31	33
				iovhbocs	1	0
		**Total : 2139 (3,55%)**			**Total : 347 (4,78%)**	**Total : 401 (7,87%)**
Sweden	SMC	1504		SWE-BRCA	190	25
	KARBAC	497				
	MISS	697				
	PKARMA	2991				
		**Total : 5689 (9,43%)**			**Total : 190 (2,62%)**	**Total : 25 (0,49%)**
UK	UKBGS	1632		OUH	1	0
	SEARCH	4057		EMBRACE	795	768
	POSH	1088		UKGRFOCR	13	4
	DIETCOMPLYF	711				
	BBCS	122				
	EPIC	703				
		**Total : 8313 (13,78%)**			**Total : 809 (11,15%)**	**Total : 772 (15,14%)**
USA	UCIBCS	490		BIDMC	43	24
	SISTER	2016		FCCC	26	11
	MEC	672		UTMDACC	25	39
	CTS	1156		NORTHSHORE	40	19
	NHS2	1606		DFCI	65	46
	NBHS	677		BRICOH	52	48
	MCBCS	925		WCP	51	18

	PLCO	868		NRG_ONCOLOGY	165	141
	CPSII	3054		OSU CCG	50	56
	BCFR-PA	132		KUMC	24	12
	MSKCC	120		UCSF	33	28
	2SISTER	1071		GEMO	84	25
	NHS	1590		BCFR-NC	33	22
	BCFR-UTAH	102		GEORGETOWN	5	0
	NC-BCFR	712		MAYO	122	74
	BCFR-NY	454		BCFR-PA	18	3
	TNBCC	373		NCI	42	21
	MMHS	384		COH	141	98
				BCFR-NY	37	25
				UPENN	240	168
				MSKCC	185	167
				BCFR-UT	67	54
				UCHICAGO	43	29
				UPITT	77	43
		**Total : 16402 (27,19%)**			**Total : 1668 (22,98%)**	**Total : 1171 (22,97%)**
		Total : 60326			Total : 7258	Total : 5100

Supplementary Table 4- Number of control subjects per country and study

Country	BCAC Study	Number of controls		CIMBA study	Number of BRCA1 controls	Number of BRCA2 controls
Australia	MCCS	978		NRG_ONCOLOGY	3	6
	ABCTB	374		KCONFAB	356	273
	ABCFS	189		BCFR-AU	14	11
	BCEES	835		VFCTG	104	130
		**Total : 2376 (5,18%)**			**Total : 477 (6,64%)**	**Total : 420 (8,32%)**
Belgium	LMBC	1268		G-FAST	69	87
		**Total : 1268 (2,76%)**			**Total : 69 (0,96%)**	**Total : 87 (1,72%)**
Canada	CBCS	817		MCGILL	30	20
	OFBCR	375		BCFR-ON	34	24
	MTLGEBCS	169		OCGN	133	107
				INHERIT	52	46
		**Total : 1361 (2,97%)**			**Total : 249 (3,47%)**	**Total : 197 (3,9%)**
Denmark	CGPS	716		CBCS	111	65
				OUH	357	258
		**Total : 716 (1,56%)**			**Total : 468 (6,51%)**	**Total : 323 (6,4%)**
Finland	HEBCS	177		HEBCS	67	63
	KBCP	245		OUH	1	0
		**Total : 422 (0,92%)**			**Total : 68 (0,95%)**	**Total : 63 (1,25%)**
France	CECILE	159		GEMO	558	314
	EPIC	370				
		**Total : 529 (1,15%)**			**Total : 558 (7,77%)**	**Total : 314 (6,22%)**
Germany	ESTHER	187		GC-HBOC	675	407
	SKKDKFZS	0		DKFZ	19	10
	GESBC	181				

	GENICA	284				
	BBCC	253				
	MARIE	289				
	BSUCH	168				
	EPIC	650				
	GC-HBOC	1593				
	HABCS	866				
		**Total : 4471 (9,74%)**			**Total : 694 (9,66%)**	**Total : 417 (8,26%)**
Greece	EPIC	180		DEMOKRITOS	85	9
	CCGP	332				
		**Total : 512 (1,12%)**			**Total : 85 (1,18%)**	**Total : 9 (0,18%)**
Israel	BCINIS	713		SMC	99	47
		**Total : 713 (1,55%)**			**Total : 99 (1,38%)**	**Total : 47 (0,93%)**
Italy	EPIC	788		CONSIT TEAM	265	127
	MBCSG	366		IOVHBOCS	92	53
				PBCS	39	1
		**Total : 1154 (2,51%)**			**Total : 396 (5,51%)**	**Total : 181 (3,59%)**
Netherlands	RBCS	240		HEBON	491	401
	EPIC	676				
	ORIGO	660				
	ABCS	189				
		**Total : 1765 (3,85%)**			**Total : 491 (6,83%)**	**Total : 401 (7,95%)**
Poland	PBCS	2045		IHCC	121	0
	SZBCS	174				
		**Total : 2219 (4,84%)**			**Total : 121 (1,68%)**	**Total : 0 (0%)**
Russia	HUBCS	119		NNPIO	22	0
		**Total : 119 (0,26%)**			**Total : 22 (0,31%)**	**Total : 0 (0%)**

Spain	BREOGAN	725		HCSC	85	77
	EPIC	311		ICO	150	163
	HCSC	0		HVH	56	65
				FPGMX	41	0
				CNIO	32	31
				IOVHBOCS	0	26
		**Total : 1036 (2,26%)**			**Total : 364 (5,07%)**	**Total : 362 (7,17%)**
Sweden	SMC	709		SWE-BRCA	237	39
	KARBAC	0				
	MISS	1545				
	PKARMA	6084				
		**Total : 8338 (18,17%)**			**Total : 237 (3,3%)**	**Total : 39 (0,77%)**
UK	UKBGS	705		EMBRACE	908	867
	SEARCH	2670		UKGRFOCR	40	12
	POSH	0		VFCTG	0	1
	DIETCOMPLY F	0				
	BBCS	442				
	EPIC	669				
	UKOPS	974				
		**Total : 5460 (11,9%)**			**Total : 948 (13,19%)**	**Total : 880 (17,44%)**
USA	UCIBCS	258		BIDMC	40	28
	SISTER	1558		FCCC	49	31
	MEC	724		UTMDACC	18	28
	CTS	610		NORTHSHORE	40	36
	NHS2	1905		DFCI	80	81
	NBHS	652		BRICOH	98	76
	MCBCS	221		WCP	137	51
	PLCO	858		NRG_ONCOLOGY	150	141

	CPSII	3029		OSU CCG	34	43
	BCFR-PA	0		KUMC	3	0
	MSKCC	0		UCSF	60	35
	2SISTER	0		GEMO	72	14
	NHS	1804		BCFR-NC	4	5
	BCFR-UTAH	0		GEORGETOWN	6	0
	NC-BCFR	148		MAYO	127	54
	BCFR-NY	27		BCFR-PA	26	3
	TNBCC	0		NCI	109	62
	MMHS	1635		COH	84	43
				BCFR-NY	25	27
				UPENN	220	178
				MSKCC	194	189
				BCFR-UT	135	97
				UCHICAGO	51	28
				UPITT	77	56
		**Total : 13429 (29,26%)**			**Total : 1839 (25,59%)**	**Total : 1306 (25,88%)**
		Total : 45888			Total : 7185	Total : 5046

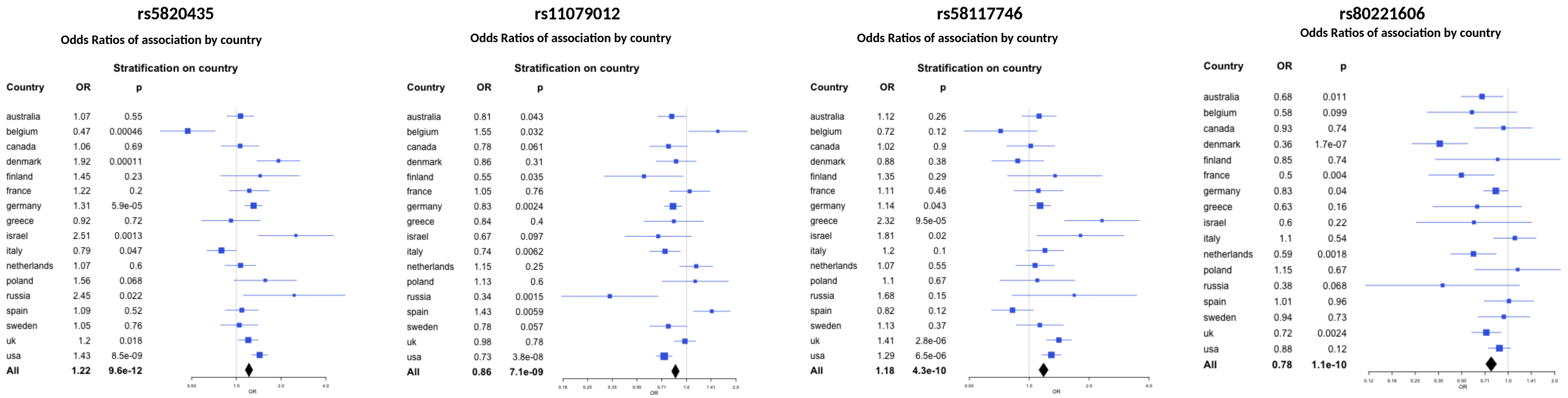

Supplementary figure 1- Heterogeneity in the SNP associations by country for the SNPs found to be associated with *BRCA1* mutation status. Forest plots show the OR estimates in the case-only regression analysis by country. The Likelihood ratio test for heterogeneity between countries was significant at $p<0.05$ for all SNPs. OR values were computed from a two sided logistic regression using a 1df lrtest adjusted for age at BC diagnosis and the first four principal components. Data are presented as punctual OR values and confidence interval. Number of individuals included for each country is detailed in Supplementary table 3. Source data are provided as a Source Data file.

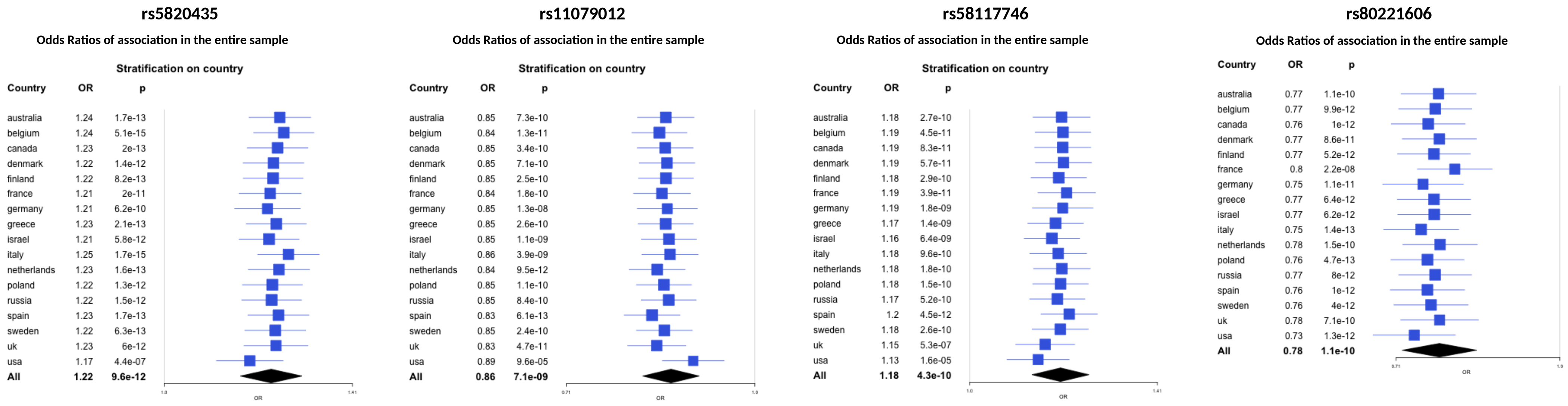

Supplementary figure 2- Sensitivity analysis for the SNPs showing associations with *BRCA1* mutation status in the case only analysis. Forest plots show the OR estimates of association in the entire sample after excluding each country in turn. "Country" indicates the country excluded in the analysis. OR values were computed from a two sided logistic regression using a 1df lrtest adjusted for age at BC diagnosis and the first four principal components. Data are presented as punctual OR values and confidence interval. Source data are provided as a Source Data file.

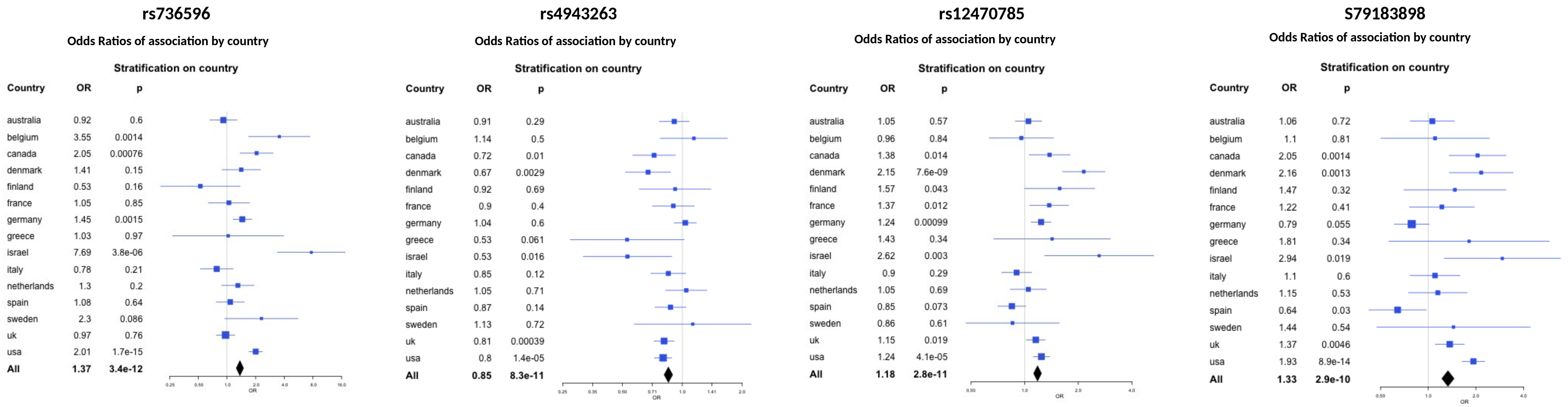

Supplementary figure 3- Heterogeneity in the SNP associations by country for the SNPs found to be associated with *BRCA2* mutation status. Forest plots show the OR estimates in the case-only regression analysis by country. The Likelihood ratio test for heterogeneity between countries was significant at p<0.05 for all SNPs. OR values were computed from a two sided logistic regression using a 1df lrtest adjusted for age at BC diagnosis and the first four principal components. Data are presented as punctual OR values and confidence interval. Source data are provided as a Source Data file.

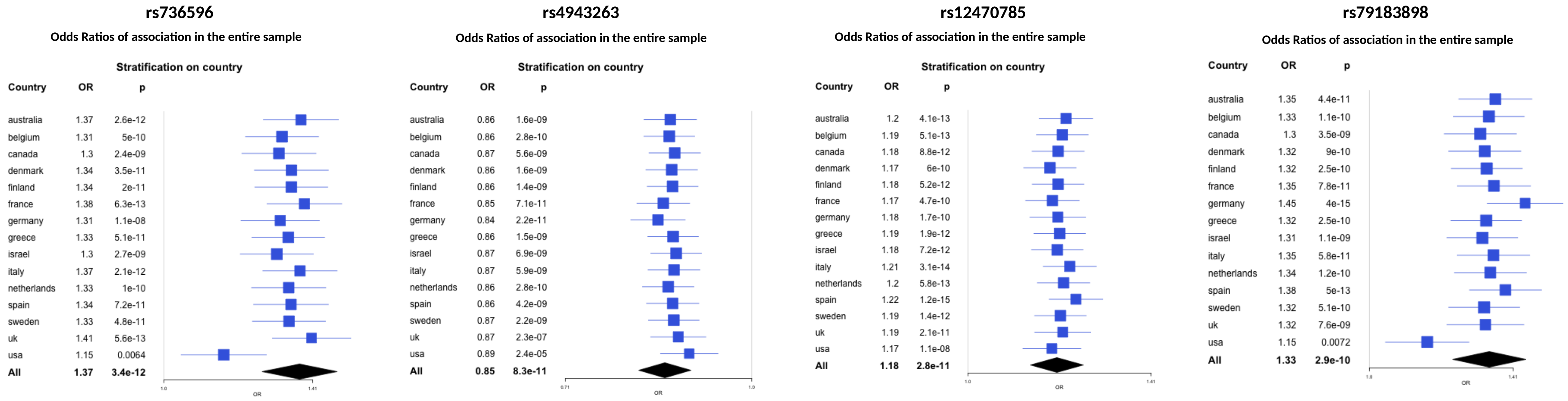

Supplementary figure 4- Sensitivity analysis for the SNPs showing associations with *BRCA2* mutation status in the case only analysis. Forest plots show the OR estimates of association in the entire sample after excluding each country in turn. “Country” indicates the country excluded in the analysis. OR values were computed from a two sided logistic regression using a 1df lrtest adjusted for age at BC diagnosis and the first four principal components. Data are presented as punctual OR values and confidence interval. Source data are provided as a Source Data file.

a.

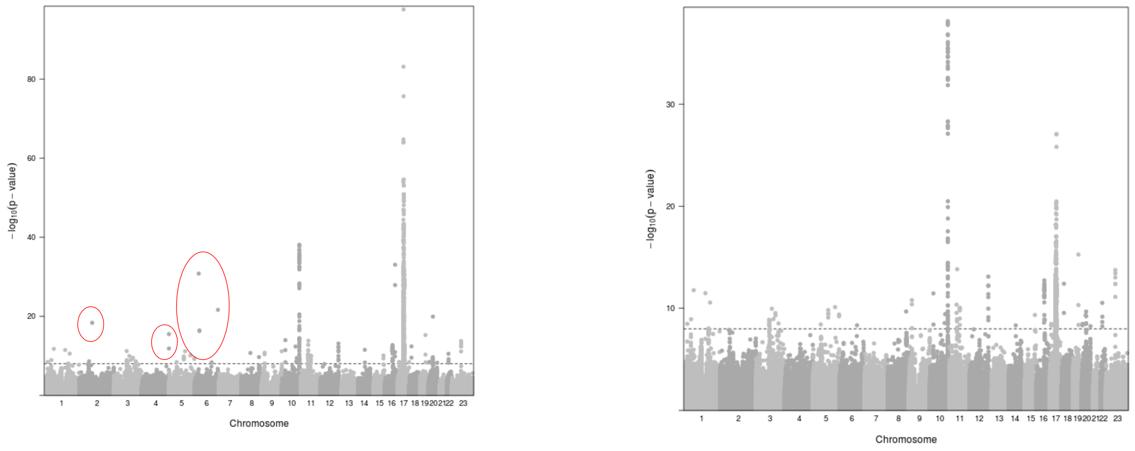

b.

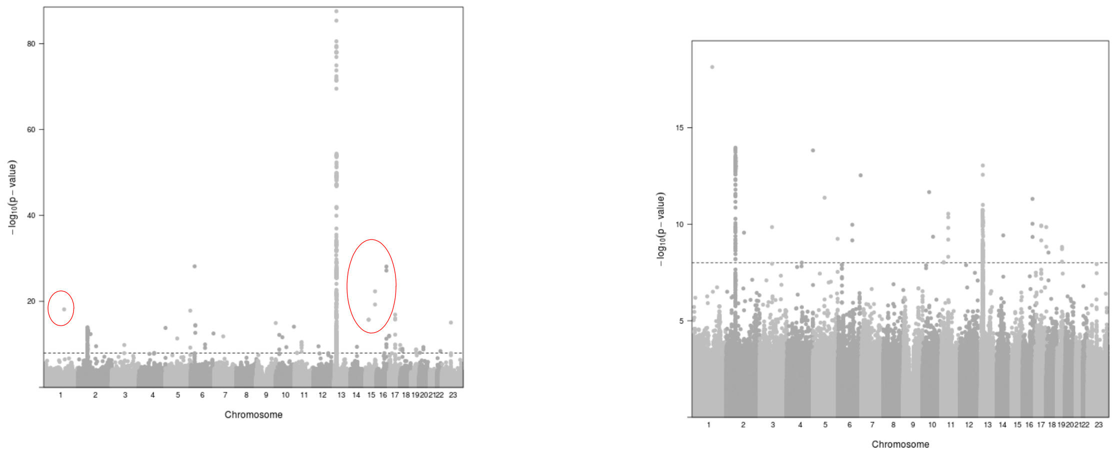

Supplementary figure 5- Impact of the control-only analysis on the case-only analysis results

Manhattan plot showing -log10(P-values) for the case-only analysis of SNPs before exclusion of the significant SNPs (i.e. in LD or ILD) in the control-only analysis (left) and after exclusions of significant SNPs in the control-only analysis (right) for **a.** *BRCA1* mutation carriers, N = 67,469 breast cancer cases (60,212 BCAC cases and 7,257 BRCA1 mutation carrier cases) and **b.** *BRCA2* mutation carriers, N = 62,822 breast cancer cases (57,725 BCAC cases and 5,097 BRCA2 mutation carrier cases). In red circles, example of SNPs excluded based on control analysis and significantly associated in case-only analysis. Grey dotted line represents the multiple testing threshold, $\alpha^*=10^{-8}$. OR values were computed from a two sided logistic regression using a 1df lrtest adjusted for age at BC diagnosis, country and the first four principal components. Source data are provided as a Source Data file.
