# Supplementary material for: A case-only study to identify genetic modifiers of breast cancer risk for BRCA1/BRCA2 mutation carriers
Source: Nat Commun. 2021 Feb 17;12:1078. doi: 10.1038/s41467-020-20496-3 (PMC7890067; doi:10.1038/s41467-020-20496-3)
Supplement: Supplementary file 3 — Description of Additional Supplementary Files [file 41467_2020_20496_MOESM3_ESM.pdf]

Description of Additional Supplementary Files

File name: Supplementary data 1
Description: SNPs previously found to be associated with breast cancer risk in Michailidou et Al. (2017): results from the case-only analyses comparing all BCAC cases or BCAC ER-negative breast cancer cases with BRCA1 breast cancer cases.

File name: Supplementary data 2
Description: SNPs previously found to be associated with breast cancer risk in Milne et Al. (2017): results from the case-only analyses comparing all BCAC cases or BCAC ER-negative breast cancer cases with BRCA1 breast cancer cases. (2017)

File name: Supplementary data 3
Description: SNPs previously found to be associated with breast cancer risk in Michailidou et Al. (2017): results from the case-only analyses comparing all BCAC cases or BCAC ER-negative breast cancer cases with BRCA2 breast cancer cases.

File name: Supplementary data 4
Description: 71 SNPs associated in the case-only analysis for BRCA1 mutation carriers (after re-imputation and before step-wise regression).

File name: Supplementary data 5
Description: 102 SNPs associated in the case-only analysis for BRCA2 mutation carriers (after re-imputation and before step-wise regression).

File name: Supplementary data 6
Description: Credible causal variants in the case-only analysis for BRCA1 mutation carriers.

File name: Supplementary data 7
Description: INQUISIT results for CCV found in the case-only analysis for BRCA1 mutation carriers.

File name: Supplementary data 8
Description: 395 credible causal variants in the case-only analysis for BRCA2 mutation carriers.

File name: Supplementary data 9
Description: INQUISIT results for CCV found in the case-only analysis for BRCA2 mutation carriers.
