# Supplementary material for: A case-only study to identify genetic modifiers of breast cancer risk for BRCA1/BRCA2 mutation carriers
Source: Nat Commun. 2021 Feb 17;12:1078. doi: 10.1038/s41467-020-20496-3 (PMC7890067; doi:10.1038/s41467-020-20496-3)
Supplement: Supplementary file 5 — Reporting Summary [file 41467_2020_20496_MOESM5_ESM.pdf]

nature research

Corresponding author(s): DBPR NCOMMS-19-33934

Last updated by author(s): 14/08/2020

Reporting Summary

Nature Research wishes to improve the reproducibility of the work that we publish. This form provides structure for consistency and transparency in reporting. For further information on Nature Research policies, see Authors & Referees and the Editorial Policy Checklist .

Statistics

For all statistical analyses, confirm that the following items are present in the figure legend, table legend, main text, or Methods section.

n/a	Confirmed	
☐	☒	The exact sample size (*n*) for each experimental group/condition, given as a discrete number and unit of measurement
☐	☒	A statement on whether measurements were taken from distinct samples or whether the same sample was measured repeatedly
☐	☒	The statistical test(s) used AND whether they are one- or two-sided<br>*Only common tests should be described solely by name; describe more complex techniques in the Methods section.*
☐	☒	A description of all covariates tested
☐	☒	A description of any assumptions or corrections, such as tests of normality and adjustment for multiple comparisons
☐	☒	A full description of the statistical parameters including central tendency (e.g. means) or other basic estimates (e.g. regression coefficient) AND variation (e.g. standard deviation) or associated estimates of uncertainty (e.g. confidence intervals)
☐	☒	For null hypothesis testing, the test statistic (e.g. *F*, *t*, *r*) with confidence intervals, effect sizes, degrees of freedom and *P* value noted<br>*Give P values as exact values whenever suitable.*
☒	☐	For Bayesian analysis, information on the choice of priors and Markov chain Monte Carlo settings
☒	☐	For hierarchical and complex designs, identification of the appropriate level for tests and full reporting of outcomes
☒	☐	Estimates of effect sizes (e.g. Cohen's *d*, Pearson's *r*), indicating how they were calculated

Our web collection on statistics for biologists contains articles on many of the points above.

Software and code

Policy information about availability of computer code

Data collection	Since data collection has been perfomed by more tahn 115 studies, we can not provide an exhaustive list of softwares used for data collection.
Data analysis	Genotypes for ~21 Million SNPs were imputed for all subjects using the 1000 Genomes Phase III data (released October 2014) as reference panel, as described in Material and Methods section page 34 . A two-stage imputation approach was used: phasing with SHAPEIT and imputation with IMPUTE2.<br>The purpose-written software PCCALC was used to compute the principal components (http://ccge.medschl.cam.ac.uk/software/pccalc/) and LOGITREGRESS to perform the association analyses (not publicly available).

For manuscripts utilizing custom algorithms or software that are central to the research but not yet described in published literature, software must be made available to editors/reviewers. We strongly encourage code deposition in a community repository (e.g. GitHub). See the Nature Research guidelines for submitting code & software for further information.

Data

Policy information about availability of data

All manuscripts must include a data availability statement. This statement should provide the following information, where applicable:

- Accession codes, unique identifiers, or web links for publicly available datasets
- A list of figures that have associated raw data
- A description of any restrictions on data availability

A subset of the data that support the findings of this study is publically available via dbGaP (see https://www.ncbi.nlm.nih.gov/gap; accessions phs001265.v1.p1 for BCAC data and phs001321.v1.p1 for CIMBA data). Requests for data can be made to the corresponding author or the Data Access Coordination Committees (DACCs) of BCAC (see http://bcac.ccge.medschl.cam.ac.uk/) and CIMBA (see http://cimba.ccge.medschl.cam.ac.uk/). BCAC DACC approval is required to access data from the ABCFS, ABCS, ABCTB, BBCC, BBCS, BCEES, BCFR-NY, BCFR-PA, BCFR-UT, BCINIS, BSUCH, CBCS, CECILE, CGPS, CTS, DIETCOMPLYF, ESTHER, GC-HBOC, GENICA, GEPARSIXTO, GESBC, HABCS, HCSC, HEBCS, HMBCS, HUBCS, KARBAC, KBCP, LMBC, MABCS, MARIE, MBCSG, MCBCS, MISS, MMHS, MTLGEBCS, NC-BCFR, OFBCR,

ORIGO, pKARMA, POSH, PREFACE, RBCS, SKKDKFZS, SUCCESSB, SUCCESSC, SZBCS, TNBCC, UCIBCS, UKBGS and UKOPS studies (Supplementary Table 1). CIMBA DACC approval is required to access data from the BCFR-ON, CONSIT TEAM, DKFZ, EMBRACE, FPGMX, GC-HBOC, GEMO, G-FAST, HEBCS, HEBON, IHCC, INHERIT, IOVHBOCS, IPOBCS, MCGILL, MODSQUAD, NAROD, OCGN, OUH and UKGRFOCR studies (Supplementary Table 2). The case-control summary results from CIMBA and BCAC consortia are publicly available and can be dowloaded at http://cimba.ccge.medschl.cam.ac.uk/projects/ and at http://bcac.ccge.medschl.cam.ac.uk/bcacdata/).

Field-specific reporting

Please select the one below that is the best fit for your research. If you are not sure, read the appropriate sections before making your selection.

[x] Life sciences [ ] Behavioural & social sciences [ ] Ecological, evolutionary & environmental sciences

For a reference copy of the document with all sections, see nature.com/documents/nr-reporting-summary-flat.pdf

Life sciences study design

All studies must disclose on these points even when the disclosure is negative.

Sample size	All the data available of BCAC and CIMBA consortia where used to performed the analyses.
Data exclusions	To minimise the chance of observing spurious associations due to differences in the distribution of BC cases in the population by tumour characteristics (defined as unselected BC cases), 3,478 BCAC cases from 4 studies were excluded because they were included in clinical trials based on breast tumour characteristics as HER-2 receptor status (see Supplementary Table 2). Because all the analyses were adjusted for country, to ensure that the number of subjects in each country stratum was large enough, we excluded the CIMBA data from any country for which there were less than ten BC cases with BRCA1 or BRCA2 mutation. Consequently, data from Poland and Russia were excluded from the BRCA2 analyses (Supplemental Table 3). Finally, duplicate subjects between BCAC and CIMBA were excluded from the BCAC data (114 and 80 subjects from the BRCA1 and BRCA2 case-only analyses, respectively; eight subjects from control-only analyses).
Replication	Data used in this analysis represent the largest currently available datasets, but it is important to replicate these observations in independent samples. This should be possible through the ongoing CONFLUENCE large-scale genotyping experiment.
Randomization	This is a observational study and constitutional SNPs can not be randomized.
Blinding	This is not a clinical trial but an observational study on constitutional genetic variants.

Reporting for specific materials, systems and methods

We require information from authors about some types of materials, experimental systems and methods used in many studies. Here, indicate whether each material, system or method listed is relevant to your study. If you are not sure if a list item applies to your research, read the appropriate section before selecting a response.

Materials & experimental systems

n/a	Involved in the study	
[x]	[ ]	Antibodies
[x]	[ ]	Eukaryotic cell lines
[x]	[ ]	Palaeontology
[x]	[ ]	Animals and other organisms
[ ]	[x]	Human research participants
[x]	[ ]	Clinical data

Methods

n/a	Involved in the study	
[x]	[ ]	ChIP-seq
[x]	[ ]	Flow cytometry
[x]	[ ]	MRI-based neuroimaging

Human research participants

Policy information about studies involving human research participants

Population characteristics	We used data from two international consortia, BCAC and CIMBA. BCAC included data from 108 studies of BC from 33 countries in North America, Europe and Australia, the majority (88%) of which were case-control studies. Data were available on 30,500 BRCA1 mutation carriers and 20,500 BRCA2 mutation carriers from 77 studies in 32 countries. A total of 188,320 BC cases and 161,669 controls were available from both consortia. All studies provided information on disease status, age at diagnosis or at interview. Oestrogen receptor status was available for 72% of BCAC cases and 71% of CIMBA cases.<br>All individuals included in these analyses are women. The mean age at BC diagnosis for mutation carrier cases in CIMBA was 42.5 years (40.9 for BRCA1 mutation carriers; 44.1 for BRCA2 mutation carriers) and 58.4 years for cases in BCAC.
Recruitment	Data analysed in this manuscript come from two international consortia, BCAC and CIMBA. The majority of BCAC cases/controls were not tested for BRCA1/2 mutations at the time of enrolment. CIMBA participants were women with pathogenic mutations in BRCA1 or BRCA2. All participants were at least 18 years old. The majority of mutation carriers were recruited through cancer genetics clinics and enrolled into national or regional studies. All subjects provided written informed consent and participated in studies with protocols approved by ethics committees at each participating institution.
Ethics oversight	The organizations that approved the protocol of the different studies used in this analysis are listed in the excel files "BCAC_ethicCommittee" and "CIMBA_ethicCommittee".

Note that full information on the approval of the study protocol must also be provided in the manuscript.
