# Supplementary material for: A case-only study to identify genetic modifiers of breast cancer risk for BRCA1/BRCA2 mutation carriers
Source: Nat Commun. 2021 Feb 17;12:1078. doi: 10.1038/s41467-020-20496-3 (PMC7890067; doi:10.1038/s41467-020-20496-3)
Supplement: Supplementary file 2 — Peer Review File [file 41467_2020_20496_MOESM2_ESM.pdf]

Reviewers' comments:

Reviewer #1 (Remarks to the Author):

The authors present the results of an interesting study which aims to identify variants that are associated with different risks of breast cancer depending no whether or not an individual carries a risk mutation in BRCA1/2. The study is sensibly-designed and clearly presented, though in places the results are somewhat overstated.

I have a number of specific comments about the manuscript:

(1) p.16 Even the BRCA1/2-carrying controls are presumably ascertained because they are related to a case, so aren't they at increased chance of carrying breast cancer-associated SNPs? Is there any evidence to suggest whether the 58+60 SNPs excluded from further analysis on the basis of the control-control analysis (but not on chr 17) are simply at increased frequency amongst BRCA1/2 carriers as their exclusion assumes. E.g. were they at similar frequencies in both mutation-carrying cases and controls - if not then they may be associated with breast cancer risk?

(2) p.17 Where a SNP has previously been shown to be associated with breast cancer risk amongst non-carriers, the authors show that, for a number of SNPs, there is a significant difference between the frequency of such SNPs in cases with and without BRCA1/2 mutations (their case-case analysis). For most of these, the estimated overall effect on breast cancer risk in BRCA1/2 carriers is of smaller magnitude than in non-carriers (sometimes in the opposite direction). It is quite possible then, that these variants have no effect on risk in BRCA1/2 mutation carriers. I suspect that the researchers lack the power (in a case-control analysis of BRCA1/2 carriers) to distinguish between a lesser effect and no effect. But they should be much clearer about this limitation - i.e. that such interaction may simply reflect variants that have no effect in BRCA1/2 mutation carriers. This is noted in the Discussion (p.28) but it states, for example, that amongst the SNPs "one leads to an association which is in the opposite direction to that observed in the general population". Presumably this may also have no effect in the mutation carriers.

(3) p.21 It's not clear in the results to what extent the 'novel' SNP associations that were found by comparing the cases from CIMBA and BCAC give different results when the two datasets were re-imputed at the same time - it is just stated that when reimputed many were then associated in the control-control analysis? I.e. how much of a problem was the separate imputation of these two datasets (despite the authors ensuring that imputation quality was cmparable between the two)? It is noted on p.30 that 28 out of 33 regions were no longer associated with risk following re-imputation, but more details of what had gone wrong here would be useful (were, for instance, the well-imputed SNPs more reliable in this context?).

(4) p.28 "This suggests that, while most SNPs associated with risk in the general population are also associated with risk in carriers, the average effects size is smaller." Why does a smaller point estimate for effect size in carriers suggest that most SNPs are also associated with risk in carriers - surely they could simply be estimates of a true OR of 1 (i.e no effect)?

(5) p.28-29 "For five of these SNPs the estimated ORs from the case-only analysis results were in the same direction... For the remaining three variants... the associations in BRCA1 or BRCA2 mutation carriers in the CIMBA data were not consistent with the observed interactions and might be artefactual" So it is an exaggeration to say in the abstract that 8 novel associated variants have been found when 3 are likely to be artifactual.

(6) p.35 I'm not sure about the logic of doing the ER- analysis for established SNPs with a threshold of 0.05 - what about a multiple-testing correction? I suspect this is a post-hoc decision as two of the reported SNPs have p-values of 0.04.

(7) p.36 For the 179 'known' breast cancer risk SNPs, the possiblity of differential effect in BRCA1/2 mutation carriers was tested in this study, using a Bonferroni correction. Can the same logic not be applied to the 'new' susceptibility loci found in the case-case analysis - i.e. test for an effect in a case-control analysis in the BCAC samples with a Bonferroni correction? It's quite possible that these variants simply have a much bigger effect in BRCA1/2 mutation carriers than in non-carriers (rather than no effect).

(8) p.58 (Fig1/2) The majority of samples (e.g. 80% of cases) appear to be non-European and are excluded (given concerns about stratification, presumably). Is it not possible to conduct a similar study on non-European populations and then compare/combine results across ethnicities? This obviously depends on the sizes of the various European populations in BCAC and CIMBA, but it would help with some of the LD issues.

Reviewer #2 (Remarks to the Author):

This is a striking team science effort to identify modifiers of BRCA1/2 breast cancer risk. The goal is to be able to provide stronger risk estimates and facilitate counselling for patients with BRCA1/2. The goals of the study were to: (1) to identify novel SNPs that modify BC risk for BRCA1 or BRCA2 mutation carriers but are not associated with risk in the general population and (2) for the known 179 BC susceptibility SNPs, assess whether there is evidence of an 478 interaction between the SNPs and BRCA1 or BRCA2 mutations and therefore evaluate whether the SNP effect size estimates applicable to mutation carriers are different. The goals are laudable and of importance in determining modifiers of risk in BRCA1/2 families. The study used an approach focusing on BRCA1/2 cases to increase power. One of the variants is at a site on 11p11.2 that is near a set of genes implicated in breast cancer function providing support for plausibility. The authors identify 8 different variants associated with risk in BRCA1/2 but not with risk in the general population.

Previous GWAS studies have suggested the 50 different SNPs are associated with risk in families with BRCA1/2 mutations. The current study that started with 179 mutations associated with breast cancer overall did not appear to confirm most of the SNPs previously associated with risk in BRCA1/2 mutation carriers. (the introduction indicates that 50 had been associated previously). The authors should discuss the apparent discrepancy. Further of the 179 SNPs associated with breast cancer risk in the general population, of the small number that were associated with risk, some were in the opposite direction. The authors should discuss and attempt to explain the discrepancy. This is mentioned in the discussion but not discussed.

The authors analysis of 179 SNPS associated with breast cancer risk previously provides interesting information. However, one key question that is not presented is whether the samples and data in this analysis were used in the identification of these SNPs in previous studies. This will help to inform the reader.

In the introduction, the authors state: Similarly, more than 50 of the common genetic BC susceptibility variants have also been shown to be associated with BC for BRCA1 and BRCA2 mutation carriers5,6,15,18,20,39–48 and their joint effects, summarised as polygenic risk scores (PRS), result in large differences in the absolute risks of developing BC for mutation carriers at the extremes of the PRS distribution49.
In the results and the data, and from the discussion.
Of the 179 known BC susceptibility SNPs identified through GWAS in the general population5–35, only 10 showed evidence of interaction with BRCA1 or BRCA2 mutation carrier status after taking the

tumour ER-status into account.
The current data seems to question the PRS derived from other studies. Can the authors clarify this apparent discrepancy.

The authors do not create a model based on the 8 novel BRCA1/2 risk alone variants and the 10 variants from the 179 SNPs previously associated to breast cancer risk for the combinatorial effect on risk with BRCA1/2 families. This was one of the concepts in the introduction for the importance of the study.

Answers to Reviewers' comments

Reviewer #1 (Remarks to the Author):

Question 1. p.16 Even the BRCA1/2-carrying controls are presumably ascertained because they are related to a case, so aren't they at increased chance of carrying breast cancer-associated SNPs? Is there any evidence to suggest whether the 58+60 SNPs excluded from further analysis on the basis of the control-control analysis (but not on chr 17) are simply at increased frequency amongst BRCA1/2 carriers as their exclusion assumes. E.g. were they at similar frequencies in both mutation-carrying cases and controls - if not then they may be associated with breast cancer risk?

Answer : *We agree that the SNPs excluded from further analysis on the basis of the control-control analysis may be associated with breast cancer risk. As we mentioned in the discussion paragraph page 29 « … based on the control-only analyses, we excluded approximately 2,000 SNPs because of their association with BRCA1 or BRCA2 mutation carrier status, which also showed an association with risk in the case-only analyses (Supplementary Figure 5). » However, the validity of the case-only analysis relies on the assumption of independence between the mutation status and the SNPs under investigation so this exclusion is necessary. Thus, the estimation of the association may be biased because of an interaction term estimate biased. We have revised the following sentence in the discussion to clarify this further:*

« While most of these associations are probably spurious, due to (intra- or inter-chromosomal) linkage disequilibrium with BRCA1 or BRCA2 mutations, it is possible that some may reflect true associations and that the higher frequency in unaffected BRCA1/2 may be because they are relatives of breast cancer cases. » (page 29).

Furthermore, , regarding the 58+60 SNPs excluded from further analysis on the basis of the control-control analysis, the results in CIMBA alone show that none of them were significantly associated with breast cancer ($p>0.01$; see tables below). Thus, to the extent to which we can evaluate in these data, these SNPs do not appear to be associated with risk, and hence the control-control associations probably reflect confounding to linkage disequilibrium or confounding due to population structure..

CIMBA results for SNPs associated (p<10-8) with BRCA1 mutation in control-only analysis.

					CIMBA results			
							Fréquence A1	
**SNP**	**Chro**	**Position**	**A1**	**A2**	**HR**	**P-value**	**controls**	**cases**
rs111325776	2	58790019	C	A	1.11	0.08	0.03781	0.04171
rs73939047	2	68023815	T	C	0.96	0.23	0.11841	0.11577
rs202195844	2	89419061	G	A	1.10	0.03	0.08595	0.09269
rs367695389	2	89551381	C	CAT	1.04	0.32	0.83631	0.84032
rs138171634	3	90502283	A	G	0.99	0.81	0.06402	0.06388
rs115633794	3	154594134	T	C	0.94	0.56	0.02077	0.01983
rs79067825	3	154623225	A	G	0.95	0.62	0.02095	0.02003
rs4145513	4	190862103	T	C	1.17	0.01	0.05215	0.05638
rs201592193	4	190889588	T	C	1.14	0.36	0.00924	0.00978
5:114255518:C:T	5	114255518	T	C	0.92	0.52	0.00913	0.00879
rs6939317	6	29766782	T	G	0.96	0.52	0.03743	0.03630
rs76632902	6	34875102	C	A	1.03	0.87	0.00540	0.00567
rs141565878	6	34886561	C	T	1.03	0.86	0.00539	0.00567
rs138376680	6	165835530	T	TGACAG	1.09	0.31	0.02511	0.02722
rs79420835	8	65062900	A	G	1.07	0.63	0.00850	0.00898
rs10961489	9	14368513	G	A	1.01	0.81	0.12154	0.12326
rs10961490	9	14370172	C	T	1.02	0.59	0.15755	0.15962
rs62532824	9	14372749	T	C	0.99	0.89	0.11202	0.11207
rs11545612	9	140135784	A	C	0.99	0.86	0.02203	0.02172
rs2484873	10	24634956	G	A	1.02	0.67	0.93684	0.93853
rs7080357	10	24693315	T	G	0.94	0.06	0.82256	0.81645
rs367959377	10	96273810	C	A	0.92	0.27	0.02420	0.02190
rs61894387	11	50537583	A	G	1.02	0.40	0.34800	0.35646
rs4963116	11	50558662	A	G	1.02	0.43	0.34076	0.34897
rs12362357	11	50569649	A	G	1.02	0.39	0.34015	0.34856
rs61894418	11	50577312	G	A	1.02	0.40	0.34290	0.35124
rs4980497	11	50594888	C	G	1.02	0.42	0.34449	0.35290
rs12361986	11	50685561	T	C	1.02	0.41	0.34038	0.34898
rs61174189	11	50703404	T	C	1.02	0.37	0.33699	0.34583
rs12361987	11	50715697	T	C	0.95	0.02	0.32335	0.31101
rs61546135	11	50717748	G	A	1.02	0.32	0.34024	0.34964
rs2193331	11	50718850	T	C	1.02	0.37	0.33906	0.34780
rs11245850	11	50719236	A	G	1.02	0.33	0.33896	0.34816
rs12360791	11	50721073	A	G	1.02	0.33	0.33889	0.34803
rs11245867	11	50749743	A	G	1.02	0.33	0.33926	0.34861
rs2160610	11	50757582	T	C	1.03	0.29	0.34239	0.35209
rs4980492	11	50758270	C	G	1.02	0.37	0.34080	0.34996
rs4980462	11	50773997	A	G	1.03	0.28	0.34118	0.35087
rs143711331	11	50776473	T	G	1.02	0.32	0.34051	0.34992
rs145046308	11	50781399	G	A	1.03	0.27	0.34544	0.35558
rs12366163	11	51319427	T	C	1.01	0.81	0.19564	0.19562
rs4282966	11	51558960	G	A	1.02	0.51	0.33630	0.34401
12:37864657:A:G	12	37864657	G	A	0.97	0.52	0.07680	0.07561
rs56272330	14	66527785	TAGGTAGGTAGGTAGGTA	G	1.00	0.96	0.01800	0.01784
rs180674352	14	66669319	T	A	0.96	0.66	0.01915	0.01935
rs113707297	15	29887141	G	A	0.93	0.26	0.03553	0.03309
rs11636010	15	90890122	G	A	1.02	0.62	0.08357	0.08642
rs11647186	16	70191223	A	G	1.01	0.87	0.04165	0.04086
16:70329353:A:T	16	70329353	T	A	1.10	0.59	0.00531	0.00587
rs936300	16	70727469	T	C	1.00	0.97	0.03391	0.03377
rs79946690	16	70735658	G	T	1.19	0.11	0.01070	0.01264
rs117446058	16	70749167	A	G	0.90	0.46	0.00878	0.00816
rs75074962	16	70771194	T	C	1.19	0.10	0.01146	0.01338
rs139361400	19	21472034	C	T	0.95	0.58	0.01602	0.01496
rs143871568	19	21583249	A	G	0.94	0.49	0.01864	0.01698
rs116958661	20	4993685	A	G	1.08	0.53	0.01345	0.01424
rs142001430	20	17950337	G	T	1.16	0.08	0.02289	0.02508
rs151202652	20	29535777	A	G	0.82	0.14	0.00970	0.00905

CIMBA results for SNPs associated (p<10-8) with BRCA2 mutation in control-only analysis.								
					**CIMBA results**			
							**Fréquence A1**	
**SNP**	**Chro**	**Position**	**A1**	**A2**	**HR**	**P-value**	**controls**	**cases**
rs41269399	1	245004623	A	G	0.90	0.1066	0.08	0.07
rs75722092	2	59360374	T	C	1.04	0.1355	0.51	0.52
rs367695389	2	89551381	C	CAT	1.02	0.7255	0.84	0.84
rs28714592	2	242879137	C	T	1.27	0.0423	0.02	0.02
rs11547065	5	179233740	A	G	1.00	0.9693	0.02	0.02
rs374043761	6	29655445	ATTTT	ATT	1.06	0.2738	0.08	0.08
rs6939317	6	29766782	T	G	1.05	0.5454	0.04	0.04
rs114525245	6	34687136	G	A	1.22	0.4216	0.00	0.00
rs76632902	6	34875102	C	A	1.06	0.7965	0.01	0.00
rs141565878	6	34886561	C	T	1.07	0.7774	0.01	0.00
rs960746	7	7863773	G	C	1.10	0.0007	0.29	0.31
7:65621488:AT:A	7	65621488	A	AT	0.99	0.8444	0.16	0.16
rs73229565	7	125134266	G	A	0.81	0.0125	0.04	0.03
rs73229580	7	125150607	C	T	0.81	0.0148	0.04	0.03
rs140195723	7	135104621	G	A	1.88	0.0072	0.01	0.01
rs10961489	9	14368513	G	A	1.04	0.4768	0.12	0.12
rs62532824	9	14372749	T	C	1.04	0.4669	0.11	0.11
rs11545612	9	140135784	A	C	1.04	0.7433	0.02	0.02
rs7080357	10	24693315	T	G	0.97	0.5595	0.82	0.82
rs67650055	10	24706788	AT	A	0.99	0.7975	0.56	0.56
rs76949305	10	24729568	C	T	0.94	0.3254	0.07	0.06
rs117325868	10	76079558	T	C	0.78	0.0281	0.02	0.02
rs367959377	10	96273810	C	A	0.85	0.0949	0.02	0.02
rs4750991	10	130488265	T	C	1.08	0.4339	0.04	0.04
rs12366163	11	51319427	T	C	0.98	0.6577	0.20	0.20
12:37932651:AG:A	12	37932651	A	AG	1.03	0.6402	0.05	0.05
12:73489053:C:T	12	73489053	T	C	0.75	0.0004	0.03	0.02
12:73490605:CAA:C	12	73490605	C	CAA	0.75	0.0004	0.03	0.02
12:108636173:G:T	12	108636173	T	G	1.19	0.1458	0.02	0.02
12:108636901:G:A	12	108636901	A	G	1.20	0.1319	0.02	0.02
rs112836370	14	70482181	C	A	1.25	0.1550	0.01	0.01
rs35618801	15	44537483	A	G	1.05	0.6398	0.02	0.02
rs34249440	15	44541581	G	C	1.05	0.6367	0.02	0.02
rs6496631	15	90670057	T	C	1.00	0.8733	0.30	0.31
rs11630423	15	90674366	C	T	1.00	0.9557	0.76	0.75
rs12443713	16	55880026	T	G	0.97	0.6481	0.05	0.05
rs11647186	16	70191223	A	G	0.99	0.8913	0.04	0.04
rs138271182	16	70478272	A	G	1.13	0.5451	0.00	0.01
rs140338721	16	70505167	A	G	1.17	0.4257	0.00	0.01
rs200277915	16	70574287	G	T	1.08	0.0309	0.21	0.22
rs11075801	16	70906720	T	G	1.08	0.1810	0.08	0.08
rs8052922	16	90157687	T	C	0.98	0.9394	0.01	0.01
rs8063327	16	90158354	C	T	1.02	0.9189	0.01	0.01
rs138116575	17	20019256	G	A	0.82	0.0434	0.02	0.02
17:30290188:C:CAG	17	30290188	CAG	C	1.25	0.0066	0.05	0.05
17:43492428:CA:C	17	43492428	C	CA	1.07	0.0806	0.14	0.15
rs2684618	17	43587576	G	A	0.83	0.0004	0.11	0.11
rs201499461	17	43589982	A	G	1.08	0.1295	0.07	0.07
rs200083999	17	43597662	T	C	1.09	0.0806	0.08	0.09
rs7502381	17	43606150	G	C	1.09	0.0740	0.08	0.08
rs7211561	17	43712623	A	T	0.99	0.8508	0.16	0.16
rs113100008	17	43789710	G	C	0.93	0.1387	0.09	0.08
rs182092771	18	15226253	A	G	0.87	0.2028	0.03	0.02
rs72642435	18	43583314	T	C	0.86	0.1333	0.02	0.02
rs6139435	20	4461931	C	G	0.97	0.6852	0.08	0.08
rs78254377	20	4686352	A	G	1.10	0.2246	0.05	0.05
rs376664737	20	29449657	T	TAA	1.06	0.0981	0.39	0.40
rs375771633	20	29449678	A	C	1.07	0.0558	0.37	0.38
22:41775915:A:G	22	41775915	G	A	1.11	0.3804	0.02	0.02
X:66431826:T:TTA	23	66431826	TTA	T	0.98	0.8639	0.01	0.01

<u>Question 2</u>. p.17 Where a SNP has previously been shown to be associated with breast cancer risk amongst non-carriers, the authors show that, for a number of SNPs, there is a significant difference between the frequency of such SNPs in cases with and without BRCA1/2 mutations (their case-case analysis). For most of these, the estimated overall effect on breast cancer risk in BRCA1/2 carriers is of smaller magnitude than in non-carriers (sometimes in the opposite direction). It is quite possible then, that these variants have no effect on risk in BRCA1/2 mutation carriers. I suspect that the researchers lack the power (in a case-control analysis of BRCA1/2 carriers) to distinguish between a

lesser effect and no effect. But they should be much clearer about this limitation - i.e. that such interaction may simply reflect variants that have no effect in BRCA1/2 mutation carriers. This is noted in the Discussion (p.28) but it states, for example, that amongst the SNPs "one leads to an association which is in the opposite direction to that observed in the general population". Presumably this may also have no effect in the mutation carriers.

Answer : We agree with reviewer's comment that the SNPs showing a smaller magnitude of association in carriers compared to non-carriers may not be associated with breast cancer risk for mutation carriers and that a "case-control" analysis in BRCA1/2 carriers alone may not have sufficient power to detect such smaller effect sizes. This is unavoidable: for the SNPs where the effect sizes are consistent with no effect, it is impossible to determine whether this reflects a weaker association in carriers or no association, because all estimates have uncertainty. We have added a sentence in the discussion page 28 to emphasize this point:

« However, distinguishing between a smaller effect size for mutation carriers compared to the general population OR estimates and no association for mutation carriers is very challenging since, even with the large sample size here, it is not possible to estimate the effect sizes for individual variants precisely."

Question 3. p.21 It's not clear in the results to what extent the 'novel' SNP associations that were found by comparing the cases from CIMBA and BCAC give different results when the two datasets were re-imputed at the same time - it is just stated that when reimputed many were then associated in the control-control analysis? I.e. how much of a problem was the separate imputation of these two datasets (despite the authors ensuring that imputation quality was cmparable between the two)? It is noted on p.30 that 28 out of 33 regions were no longer associated with risk following re-imputation, but more details of what had gone wrong here would be useful (were, for instance, the well-imputed SNPs more reliable in this context?).

Answer : As requested by the reviewer, here are more details on the impact of the re-imputation on the results.

When case-only analyses were first performed on the independently imputed BCAC (population) and CIMBA (BRCA1/2 mutation carriers) data, 492 SNPs were significantly associated ($p < 10^{-8}$) with BRCA1 / 2 status, 219 in the BRCA1 analysis and 273 in the BRCA2 analysis. Among the SNPs associated with BRCA1 / 2 status, 16 SNPs were genotyped and 476 were imputed. The quality of imputation as captured by the r^2 statistic was greater than 0.5, with an average r2 equal to 0.93 (Table below) and the frequency of the minor allele varied between 0.01 and 0.49. Only 59 imputed SNPs (12%) had a frequency less than 0.05.

Table : Quality of imputation of SNPs
***Before :** number of SNPs significantly associated with BRCA1 or BRCA2 status in the case-only analyses by r^2 performed on CIMBA and BCAC data imputed separately*
***After :** number of SNP still significantly associated with BRCA1 or BRCA2 status in the case-only analyses by r^2 performed on CIMBA and BCAC reimputed data*

		**imputed SNPs – r^2**						
		**[0.5–0.6[**	**[0.6–0.7[**	**[0.7–0.8[**	**[0.8–0.9[**	**[0.90–0.95[**	**[0.95–1[**	**Genotyped**
**BRCA1**	**Before**	7 (1.5 %)	0 (0.0 %)	2 (0.4 %)	12 (2.5 %)	36 (7.6 %)	155 (32.6 %)	7
	**After**	0 (0.0 %)	0 (0.0 %)	1 (1.6 %)	2 (3.1 %)	7 (10.9 %)	47 (84.3 %)	7
**BRCA2**	**Before**	19 (4.1 %)	5 (1.1 %)	9 (1.9 %)	17 (3.6 %)	40 (8.4 %)	174 (36.6 %)	9
	**After**	0 (0.0 %)	0 (0.0 %)	1 (0.01 %)	0 (0.0 %)	1 (0.01 %)	78 (97.5 %)	9

These 476 imputed SNPs are located in 33 different regions. All these regions (+/- 500 kb of the most significant SNP in the region) were re-imputed. 92% of these 476 imputed SNPs were found to have a larger p-value after imputation (Figure below) and only 28% (137 SNPs) remained significant at p <10-8 . These 137 SNPs (57 SNPs for BRCA1 analysis and 80 for BRCA2 analysis) are located in 6 regions.
These 137 SNPs have a quality of imputation higher than 0.7 and more than 91% of them have a r2> 0.95.

15.2% of the SNPs with MAF< 5% and 32.6% of SNPs with MAF>5% remained significant after re-imputation . As expected, the biggest change was observed for rare SNPs

Table : Distribution of SNPs after re-imputation according to p-value and allelic frequency (MAF).

**MAF**	***p-value* after re-imputation**	
	**≤ 10^{-8}**	**> 10^{-8}**
**< 0.05**	9 (15.2 %)	50 (84.7 %)
**≥ 0.05**	136 (32.6 %)	281 (67.4 %)

Figure below shows the distribution of the differences between the log(p-value)s obtained before and after re-imputation with, on the abscissa, the difference between the 2 p-values. For example, for 2 p-values equal to 10^{-10} and 10^{-6} before and after re-imputation, the difference will be 4. Only 24 of 476 SNPs have a lower p-value after re-imputation. Among the remaining 452 SNPs, 329 were no longer significant after re-imputation.

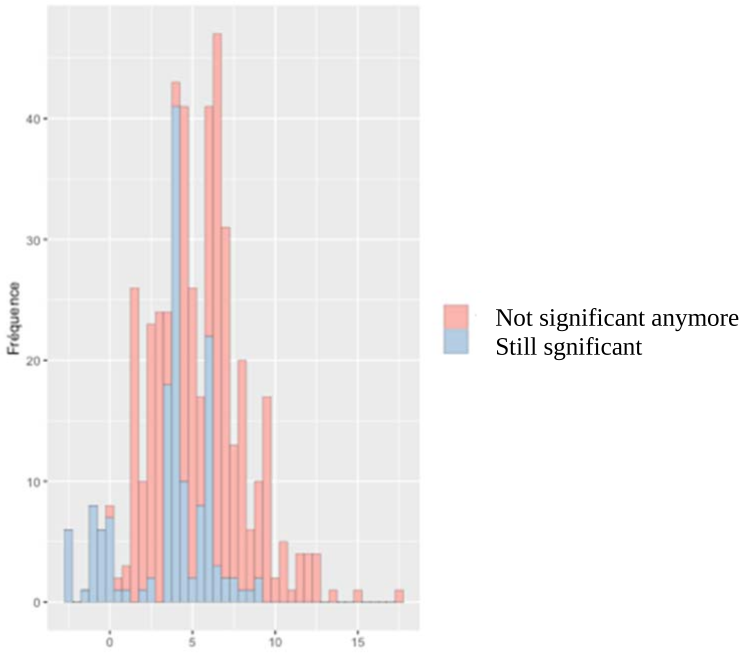

Difference between – Log10(p) before and after reimputation

Moreover, as shown in the Figure below, although the differences between the ORs before and after re-imputation are not large, 90% of the SNPs are associated with a lower OR after re-imputation. Only 16 SNPs had an identical OR (defined as an absolute difference of less than 10^{-2} between the ORs from the two analyses).

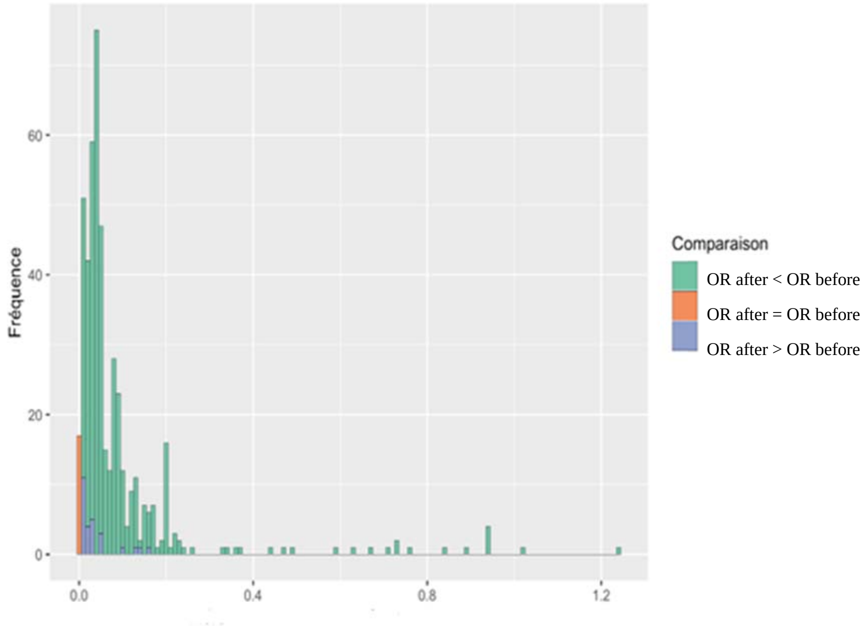

In addition to the 153 SNPs remaining significant after re-imputation (137 imputed and 16 genotyped SNPs), 92 new SNPs had p<10-8 after analyzing the re-imputed data.

These results highlight the importance of imputing cases and controls jointly in GWAS. Our manuscript focuses on reporting the associations only for SNPs of the 5 regions which were significant after joint re-imputation.

Re-imputation was carried out only for the regions found to be significant in the analysis based on data from the separate imputation in the BCAC and CIMBA subjects. Re-imputation was not performed genome-wide due to computational constraints. This may have led to some false negatives. However, our strategy should have eliminated false associations due to poor imputation ***and that the associations that we report as significant in the present manuscript are robust to the imputation process.***

These details could not be added in the manuscript because of the text length limitations and to ensure the text is not overly complicated. However, we would be happy to add any aspects of these results at the discretion of the editor.
We already include a summary of these issues in the discussion on page 30 which we highlight below for the reviewer's attention:

« Our findings highlight the importance of imputation in GWAS. The imputed genome-wide genotype data used in the main case-only association analyses were based on carrying out the imputation separately for the BC cases from BCAC and CIMBA. We found that 28 out of the 33 regions associated with BRCA1 or BRCA2 mutation carrier status were no longer associated with risk after re-imputing all samples together. By re-imputing all the data together ***we ensured that the associations observed for the remaining regions are robust to potential differences in the imputation accuracy between the BCAC and CIMBA samples****.*
Under our analytical strategy, only the regions for which evidence of associated with BC risk was observed were re-imputed using all BCAC and CIMBA samples combined. This re-imputation was not done at

genome-wide level due to computational constraints and this may have led to false-negative associations being excluded for further evaluation as potential novel modifiers. Future analyses should aim to analyse the genome-wide associations after the genome-wide re-imputation across the combined BCAC and CIMBA dataset. However, our approach using joint one-step imputation should have ensured that associations we report (all of which are common SNPs with imputation scores>0.5) are not driven by inaccuracies in imputation.»

Question 4. p.28 "This suggests that. while most SNPs associated with risk in the general population are also associated with risk in carriers. the average effects size is smaller." Why does a smaller point estimate for effect size in carriers suggest that most SNPs are also associated with risk in carriers - surely they could simply be estimates of a true OR of 1 (i.e no effect)?

Answer : *While the effect sizes are, on average, smaller in the carriers, they are still generally in the same direction. For example, for BRCA2 carriers 125/157 SNPs have a predicted effect size in the same direction as in non-carriers. This is also consistent with the fact that PRS in developed in the general population is also predictive in carriers. We have clarified this section by re-writing as follows:*

« Taken together, these results suggest while most SNPs associated with risk in the general population are associated with risk for mutation carriers, the average effect sizes for mutation carriers are smaller. These findings are in line with the previous results of Kuchenbaecker et al.[49] and suggest that a PRS built using data from the general population will have a smaller effect size for BRCA1/2 mutation carriers.»

Question 5. p.28-29 "For five of these SNPs the estimated ORs from the case-only analysis results were in the same direction... For the remaining three variants... the associations in BRCA1 or BRCA2 mutation carriers in the CIMBA data were not consistent with the observed interactions and might be artefactual" So it is an exaggeration to say in the abstract that 8 novel associated variants have been found when 3 are likely to be artifactual.

Answer : *We agree with the reviewer's comment and we modify the abstract :*

"We identified robust novel associations with BC for 2 variants for BRCA1 and 3 for BRCA2 mutation carriers, $P<10^{-8}$, at 5 loci, which are not associated with risk in the general population."

Question 6. p.35 I'm not sure about the logic of doing the ER- analysis for established SNPs with a threshold of 0.05 - what about a multiple-testing correction? I suspect this is a post-hoc decision as two of the reported SNPs have p-values of 0.04.

Answer : *We had in fact first adjusted for multiple-testing in the analysis of established SNPs for BRCA1 in the overall case-only analysis. After selecting the SNPs passing the multiple testing significance threshold in this analysis, a significance threshold of 5% was used only for the last step in the ER-negative case-only analysis (figure 3). This was to ensure that some evidence of association remained after restricting the analysis to ER-negative cases in BCAC. The paragraph was probably confusing and has now been rephrased on pages 35-36.*

« … in the BRCA1 analysis, SNPs were considered to be associated with mutation carrier status only if they were also associated in the ER-negative case-only analysis at a prior defined significance threshold of 10-7

for novel SNP modifiers (cf. figure 4) and of 0.05 for the established BC susceptibility SNPs after a pre-selection at P<2.9 10-4 of the BRCA1 case-only analysis (cf. Figure 3) ».

Question 7. p.36 For the 179 'known' breast cancer risk SNPs. the possibility of differential effect in BRCA1/2 mutation carriers was tested in this study. using a Bonferroni correction. Can the same logic not be applied to the 'new' susceptibility loci found in the case-case analysis - i.e. test for an effect in a case-control analysis in the BCAC samples with a Bonferroni correction? It's quite possible that these variants simply have a much bigger effect in BRCA1/2 mutation carriers than in non-carriers (rather than no effect).

Answer : BCAC results for the 8 new SNPs are available on table 3 and 4. Although we did not explicitly apply a multiple testing correction, none would be significant on this basis (smallest p=0.01; Bonferoni correction p=0.05/8=0.006).

Question 8. p.58 (Fig1/2) The majority of samples (e.g. 80% of cases) appear to be non-European and are excluded (given concerns about stratification. presumably). Is it not possible to conduct a similar study on non-European populations and then compare/combine results across ethnicities? This obviously depends on the sizes of the various European populations in BCAC and CIMBA. but it would help with some of the LD issues.

Answer : Unfortunately it was not possible to carry out any analyses for non-European populations. The CIMBA genotyping was targeted to European ancestry mutation carriers, with a small number of carriers of Asian ancestry (N=729 BRCA1/2 in total, and only 500 with breast cancer). The number of African ancestry mutation carriers in CIMBA is extremely small and do not have GWAS genotyping. Therefore it was not possible to perform any meaningful analyses in samples of non-European ancestry. Large genotyping efforts in the future (e.g. as part planned CONFLUENCE experiment (https://dceg.cancer.gov/research/cancer-types/breast-cancer/confluence-project) may allow these analyses to be extended to non-European populations..

Reviewer #2 (Remarks to the Author):

Question 1. Previous GWAS studies have suggested the 50 different SNPs are associated with risk in families with BRCA1/2 mutations. The current study that started with 179 mutations associated with breast cancer overall did not appear to confirm most of the SNPs previously associated with risk in BRCA1/2 mutation carriers. (the introduction indicates that 50 had been associated previously). The authors should discuss the apparent discrepancy.

Answer : *We believe there is some misunderstanding. This analysis did not investigate the associations of known SNPs specifically in BRCA1 and BRCA2 mutation carriers. Instead we investigated the evidence of interaction between the known susceptibility SNPs with BRCA1/2 mutation status. The 50 SNPs previously found to be associated with breast cancer risk in BRCA1/2 mutation carriers are all included among the 179 SNPs found associated with breast cancer risk in the general population. For these 50 SNPs, which were previously shown to be associated with risk for BRCA1/2 carriers, the current case-case analysis showed no significant evidence of interaction. Thus, this study suggests that these 50 SNPs confer the same relative effect on breast cancer risk in mutation carriers and in the general population.*

Question 2. Further of the 179 SNPs associated with breast cancer risk in the general population of the small number that were associated with risk some were in the opposite direction. The authors should discuss and attempt to explain the discrepancy. This is mentioned in the discussion but not discussed.

Answer : *Following the clarification in the previous comment, for two of the 10 SNPs (on 1p22.3 and on 6q14.1) for which an interaction was observed with BRCA1 or BRCA2 mutation carrier status, the results suggest that there is association in the opposite direction to that observed in the general population. This is not necessarily a discrepancy, but a consequence of the observed interaction. The biological basis for the observed statistical interactions is an interesting question for future research; however, since the causal variants and target genes underlying these signals are unknown, and given the lack of space, we believe it not helpful to speculate here.*

Question 3. The authors analysis of 179 SNPS associated with breast cancer risk previously provides interesting information. However one key question that is not presented is whether the samples and data in this analysis were used in the identification of these SNPs in previous studies. This will help to inform the reader.

Answer : *The reviewer is right. The BCAC samples in the present analysis were used in the identification of these SNPs in previous studies and this has now been clarified in the discussion on page 28 : « The effect sizes in the general population may be somewhat exaggerated as the BCAC dataset used here contributed to the discovery of most of the loci, although this effect is likely to be small as most loci are highly significant and the effects have been replicated in independent datasets».*

Question 4. In the introduction the authors state: Similarly more than 50 of the common genetic BC susceptibility variants have also been shown to be associated with BC for BRCA1 and BRCA2 mutation carriers5.6.15.18.20.39–48 and their joint effects summarised as polygenic risk scores (PRS) result in large differences in the absolute risks of developing BC for mutation carriers at the extremes of the PRS distribution49. In the results and the data and from the discussion. Of the 179 known BC susceptibility SNPs identified through GWAS in the general population5–35. only 10 showed evidence of interaction with BRCA1 or BRCA2 mutation carrier status after taking the

tumour ER-status into account. The current data seems to question the PRS derived from other studies. Can the authors clarify this apparent discrepancy.

Answer : *As indicated above, the previous and present analyses are addressing different questions. The present analysis has only looked at interactions between BRCA1/2 mutations and known breast cancer susceptibility SNPs. It has not investigated the evidence of association specifically in BRCA1/2 mutation carriers. In fact, as clarified in response to comments above, the present analysis suggests that the majority of the known susceptibility SNPs also may have similar associations with risk for BRCA1/2 carriers. The present results therefore do not contradict the observation that the PRS derived in population-based studies is also predictive of risk in carriers. The present findings, i.e. that 10 SNPs showed evidence of interaction and the observation that 82% of the 179 SNPs showed a predicted OR of association for mutation carriers which is closer to 1 than the one estimated in the general population, suggest that a PRS built using data from the general population will have a smaller effect size for BRCA1/2 mutation carriers. This is fact completely consistent with the analyses of the PRS reported by Kuchenbaecker et al JNCI 2017.*

Question 5. The authors do not create a model based on the 8 novel BRCA1/2 risk alone variants and the 10 variants from the 179 SNPs previously associated to breast cancer risk for the combinatorial effect on risk with BRCA1/2 families. This was one of the concepts in the introduction for the importance of the study.

Answer *: We agree that this is an important issue, building BRCA1/2-specific PRS. However, such a PRS will require a validation in independent samples and this was not possible in the present analysis, which utilize all the available data in the SNP discovery. We expect that this will be possible in the future as larger datasets become available.*

REVIEWERS' COMMENTS:

Reviewer #1 (Remarks to the Author):

The authors have answered all my queries throughly and to my satisfaction, so I'm happy to recommend this important piece of research for publication.

Reviewer #2 (Remarks to the Author):

I thank the authors for their clarifications. However, many of the points raised while discussed in the response to reviews have not been clarified in the text. This includes requests for interpretation of data, potential mechanisms underlying the data and a need for a confirmation set.

Answers to Reviewers' comments

Reviewer #1 (Remarks to the Author):

The authors have answered all my queries throughly and to my satisfaction, so I'm happy to recommend this important piece of research for publication.

Answer: We thank again Reviewer #1 for his/her valuable comments.

Reviewer #2 (Remarks to the Author):

I thank the authors for their clarifications. However, many of the points raised while discussed in the response to reviews have not been clarified in the text. This includes requests for interpretation of data, potential mechanisms underlying the data and a need for a confirmation set.

Answer: Paragraphs in the discussion has been added to comment on interpretation of data, potential mechanisms the data and a need for a confirmation set on page 20 :

« However, distinguishing between a smaller effect size for mutation carriers compared to the general population OR estimates and no association for mutation carriers is very challenging since, even with the large sample size here, it is not possible to estimate precisely the effect sizes for individual variants. Larger sample sizes will be required for this purpose. Determining the precise effects of the SNPs in BRCA1 and BRCA2 mutation carriers will provide insights for understanding the biological basis of cancer development associated with BRCA1 and BRCA2 mutations"

And on page 24:

« …. These represent the largest currently available datasets, but it is important to replicate these observations in independent samples. This should be possible through the ongoing CONFLUENCE large-scale genotyping experiment (https://dceg.cancer.gov/research/cancer-types/breast-cancer/confluence-project). More detailed fine mapping and functional analysis will be required to elucidate the role of the novel variants identified in BC development for BRCA1 and BRCA2 mutation carriers. »
